# Nicotinamide Nucleotide Transhydrogenase as a Novel Treatment Target in Adrenocortical Carcinoma

**DOI:** 10.1210/en.2018-00014

**Published:** 2018-04-20

**Authors:** Vasileios Chortis, Angela E Taylor, Craig L Doig, Mark D Walsh, Eirini Meimaridou, Carl Jenkinson, Giovanny Rodriguez-Blanco, Cristina L Ronchi, Alisha Jafri, Louise A Metherell, Daniel Hebenstreit, Warwick B Dunn, Wiebke Arlt, Paul A Foster

**Affiliations:** 1 Institute of Metabolism and Systems Research, University of Birmingham, Birmingham, United Kingdom; 2 Centre for Endocrinology, Diabetes and Metabolism, Birmingham Health Partners, Birmingham, United Kingdom; 3 School of Life Sciences, University of Warwick, Warwick, United Kingdom; 4 Centre for Endocrinology, Queen Mary University of London, William Harvey Research Institute, Barts and the London School of Medicine and Dentistry, London, United Kingdom; 5 School of Biosciences, University of Birmingham, Birmingham, United Kingdom; 6 Phenome Centre Birmingham, University of Birmingham, Birmingham, United Kingdom

## Abstract

Adrenocortical carcinoma (ACC) is an aggressive malignancy with poor response to chemotherapy. In this study, we evaluated a potential new treatment target for ACC, focusing on the mitochondrial reduced form of NAD phosphate (NADPH) generator nicotinamide nucleotide transhydrogenase (NNT). NNT has a central role within mitochondrial antioxidant pathways, protecting cells from oxidative stress. Inactivating human NNT mutations result in congenital adrenal insufficiency. We hypothesized that NNT silencing in ACC cells will induce toxic levels of oxidative stress. To explore this, we transiently knocked down NNT in NCI-H295R ACC cells. As predicted, this manipulation increased intracellular levels of oxidative stress; this resulted in a pronounced suppression of cell proliferation and higher apoptotic rates, as well as sensitization of cells to chemically induced oxidative stress. Steroidogenesis was paradoxically stimulated by NNT loss, as demonstrated by mass spectrometry–based steroid profiling. Next, we generated a stable NNT knockdown model in the same cell line to investigate the longer lasting effects of NNT silencing. After long-term culture, cells adapted metabolically to chronic NNT knockdown, restoring their redox balance and resilience to oxidative stress, although their proliferation remained suppressed. This was associated with higher rates of oxygen consumption. The molecular pathways underpinning these responses were explored in detail by RNA sequencing and nontargeted metabolome analysis, revealing major alterations in nucleotide synthesis, protein folding, and polyamine metabolism. This study provides preclinical evidence of the therapeutic merit of antioxidant targeting in ACC as well as illuminating the long-term adaptive response of cells to oxidative stress.

## Teaser

We explore antioxidant targeting as a new therapeutic approach in adrenocortical carcinoma through a combination of transient and stable gene knockdown models *in vitro*.

Adrenocortical carcinoma (ACC) is a rare but aggressive malignancy. Most patients present with, or eventually develop, metastatic disease, which shows limited or no responsiveness to cytotoxic chemotherapy (1, 2). A recent randomized trial revealed a median survival of <15 months for patients with disseminated disease receiving combination chemotherapy (3). Glucocorticoid or androgen excess often constitutes an additional clinical burden on ACC patients, undermining their quality of life (1). Unfortunately, the obvious need for more effective medical treatment options in ACC patients remains unmet, despite the remarkable progress in our understanding of the molecular biology of ACC in the last two decades (1).

Recent genetic studies have provided new insights into adrenal pathophysiology, revealing that inactivating mutations in the gene encoding the antioxidant enzyme nicotinamide nucleotide transhydrogenase (NNT) underlie a rare, hereditary form of primary adrenal insufficiency (4). Affected patients present in early childhood with failure to thrive, hypotension, and hypoglycemia, due to the inability of adrenal glands to produce sufficient cortisol (4). Intriguingly, despite the key role of NNT in preserving cellular redox balance and its ubiquitous expression, the adrenal glands are the only affected organ in most patients; this observation suggests a selective sensitivity of the adrenal glands to NNT loss (4, 5). Supportive of this, NNT-deficient mice harbor adrenal glands with disorganized cortical architecture and high apoptotic rates in their adrenal zona fasciculata, the location of glucocorticoid synthesis, but no other abnormality (4).

**Figure 1.**
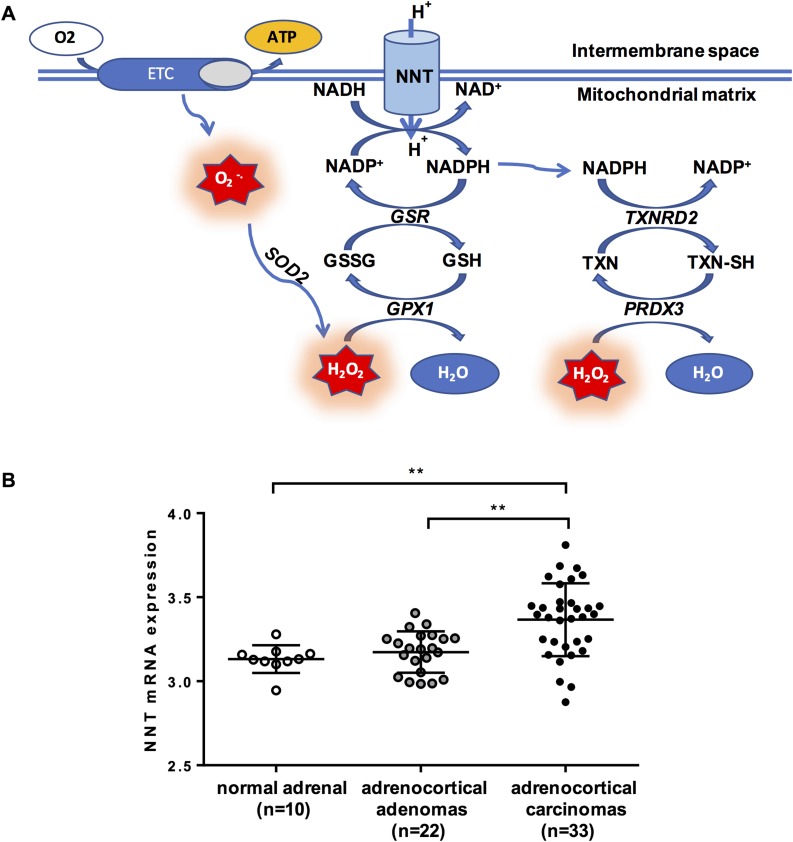
(A) Interaction between NNT and the mitochondrial antioxidant pathways. (B) NNT expression in patients with ACC (n = 33), compared with patients with adrenocortical adenomas (n = 22) and healthy adrenals (n = 10). Gene expression was quantile normalized and log transformed as described (12); bars represent median and interquartile range. Gene expression across groups was compared by applying the Kruskal–Wallis test, followed by a *post hoc* Bonferroni test. **P < 0.01. ATP, adenosine triphosphate; ETC, electron transfer chain; GPX1, glutathione peroxidase 1; GSR, glutathione reductase; H_2_O_2_, hydrogen peroxide; O_2_^·−^, superoxide; SOD2, superoxide dismutase 2; PRDX3, peroxiredoxin 3; TXN, oxidized thioredoxin; TXNRD2, thioredoxin reductase 2; TXN-SH, reduced thioredoxin.

NNT is a dimeric proton pump that resides in the inner mitochondrial membrane of eukaryotic cells and uses the transmembrane proton gradient to catalyze the transfer of reducing equivalents from reduced NAD (NADH) to NAD phosphate (NADP)^+^, according to the reaction: $\text{NADH}+{\text{NADP}}^{+}+{\text{H}}_{\text{Intermembrane}}^{+}↔{\text{NAD}}^{+}+\text{NADPH}+{\text{H}}_{\text{matrix}}^{+}$ (6, 7). The reduced form of NADP (NADPH) is an essential donor of reducing power to the two main mitochondrial antioxidant pathways, the glutathione and the thioredoxin pathways, which protect the mitochondria from the deleterious effects of oxidative stress with their capacity to detoxify reactive oxygen species (ROS; *e.g.*, hydrogen peroxide). ROS, the molecular mediators of oxidative stress, are continuously produced within the mitochondria by electron leakage along the respiratory chain complexes and the tricarboxylic acid (TCA) cycle; in adrenocortical mitochondria, steroidogenesis represents an important additional source of ROS (8–11). Excessive levels of oxidative stress lead to irreversible DNA, protein, and lipid damage, which can culminate in apoptotic cell death (Fig. 1A) (13).

Drawing on these data, which indicate a distinct metabolic vulnerability of the adrenal cortex to oxidative stress, we explored the value of antioxidant targeting as a novel therapeutic approach in ACC, focusing on NNT as a putative treatment target. Interrogating a publicly available whole-genome gene expression database (12), we observed that NNT is upregulated in ACCs in comparison with benign adrenocortical adenomas and healthy adrenals (Fig. 1B). Therefore, we hypothesized that NNT silencing in ACC cells will impair their antioxidant capacity and lead to progressive accumulation of ROS, inducing unsustainable oxidative toxicity within the mitochondria and eventually triggering cellular apoptosis. We also postulated that steroidogenesis will be suppressed as a result of NADPH depletion and/or increased oxidative stress.

## Materials and Methods

### Cell culture protocol and cell line validation

NCI-H295R (RRID: CVCL_0458) ACC cells (passage 10 to 25) were cultured under standard conditions using DMEM/Ham F-12 medium (Gibco/Thermo Fisher, Waltham, MA) supplemented with 2.5% Nu serum (Corning, New York, NY), 1% penicillin-streptomycin (Gibco/Thermo Fisher), and 1% ITS+ universal cell culture premix (Corning). Cell line identity was confirmed through short tandem repeat genetic analysis performed by the DNA Diagnostics Company (London, United Kingdom) followed by comparison with genetic profiles provided by the American Tissue Culture Collection (http://www.lgcstandards-atcc.org) (Supplemental Table 1).

### Small interfering RNA transfection

Transient NNT gene silencing was achieved through transfection of NCI-H295R cells with small interfering RNA (siRNA), using Viromer Blue (Lipocalyx, Halle, Germany) molecules as transfection vehicles. Three alternative siRNAs targeting different areas of the NNT gene were tried (HSS118900, HSS118901, and HSS118902; Life Technologies/Thermo Fisher, Waltham, MA), and the one exhibiting the most consistent efficiency in knocking down NNT (HSS118902) was selected for subsequent experiments. HSS118901 was used to corroborate results in proliferation and apoptosis assays, whose results are open to confounding by off-target effects. A scrambled (SCR), nonsense siRNA (Silencer Select 1 negative control; Life Technologies) was used as negative control (SCR siRNA). Viromer-siRNA transfection was performed according to the manufacturer’s instructions in six-well plates (300,000 cells per well) and 96-well plates (6000 to 8000 cells per well).

### Short hairpin RNA transfection

Lentiviral vectors were obtained from Dharmacon (Lafayette, CO) in a p.GIPZ backbone and contained five short hairpin RNAs (shRNAs) specific for human NNT (RHS4430-98851990, RHS4430-98913600, RHS4430-98524425, RHS4430-101033169, and RHS4430-101025114) under the control of the cytomegalovirus promoter, as well as the puromycin resistance and green fluorescence protein genes. Vectors expressing nonsense, SCR shRNA were used as negative controls. HEK293T cells (packaging cells; RRID: CVCL_0063) were transfected with the shRNA particles by Lipofectamine transfection (Thermo Fisher), according to the manufacturer’s instructions. Cell media containing the viral particles were collected 48 to 72 hours posttransfection and used to transduce NCI-H295R cells. Four days after transfection, green fluorescence protein–positive cells were selected in 4 μg/mL puromycin. Transduction efficiency was determined by fluorescence microscopy and Western blotting for NNT expression.

### Gene expression

Gene expression for NNT and steroidogenic enzymes was evaluated at a transcriptional level by quantitative real-time PCR (qRT-PCR). RNA extraction was performed using the RNeasy Mini kit (Qiagen, Hilden, Germany) following the manufacturer’s instructions. Reverse transcription to generate cDNA was carried out using the Tetro cDNA synthesis kit (Bioline, London, United Kingdom), following the manufacturer’s instructions (500 to 2000 ng of RNA per reaction was used). cDNA concentration was determined by use of a fluorescent DNA dye (Quant-iT PicoGreen double-stranded DNA reagent, Thermo Fisher), comparing sample fluorescence to the fluorescence exhibited by a dilution series of samples of known concentrations (Wallac Victor 1420 multilabel counter). Gene expression was then quantified by qRT-PCR using the the TaqMan gene expression system (Thermo Fisher). Reactions were run in a ABI 7500 qRT-PCR analyzer [50°C incubation for 2 minutes, 95°C for 10 minutes, followed by 40 cycles of 95°C for 15 seconds (denaturation) and then 60°C for 1 minute (annealing–extension); Applied Biosystems/Thermo Fisher, Foster City, CA]. All reactions were normalized against the housekeeping gene RPLPO (large ribosomal protein). Data are expressed as *Δ*Ct values [*Δ*Ct = (Ct of the target gene) – (Ct of the housekeeping gene)] or fold change to control cells (2^−ΔΔCt^), where *ΔΔ*Ct = *Δ*Ct [NNT knockdown (KD) cells] – *Δ*Ct (control cells).

### Protein expression

Protein lysate generation was performed by applying radioimmunoprecipitation assay buffer (Sigma-Aldrich, St. Louis, MO) with protease inhibitor cocktail (Sigma-Aldrich) to adherent cells grown in six-well plates and subsequent collection by scraping. Total protein concentration was estimated colorimetrically using the BCA protein assay kit (Thermo Fisher) as per the manufacturer’s instructions, measuring absorbance at 560 nm (1420 multilabel counter; Wallac Victor). NNT protein expression level was assessed by Western blotting. Samples were run in 10% SDS-PAGE gels (Thermo Fisher) and transferred to a nitrocellulose membrane using the iBlot dry transfer system (Thermo Fisher). Membranes were subsequently probed with anti-NNT antibody produced in rabbit (HPA004829; Sigma-Aldrich; RRID: AB_1079495) at a 1:500 dilution and secondary anti-rabbit antibody (sc-2030; Santa Cruz Biotechnology, Dallas, TX; RRID: AB_631747) at a 1:2000 dilution. *β*-Actin was used as control protein (primary antibody A5441; RRID: AB_476744) from Sigma-Aldrich and secondary anti-mouse antibody from Santa Cruz Biotechnology (sc-2005; RRID: AB_631736) at dilutions of 1:10,000 and 1:20,000, respectively.

### Reduced to oxidized glutathione ratio

Total cell glutathione [reduced glutathione (GSH) plus oxidized glutathione (GSSG)] and GSSG were measured by luminescence in cells growing in opaque-walled 96-well plates, using the GSH/GSSG-Glo assay (Promega, Madison, WI) according to the manufacturer’s instructions. The resulting luminescent signal was measured in a Wallac Victor 1420 multilabel counter, using triplicate samples per treatment group and subtracting blank measurements to produce net results. GSH/GSSG ratios were calculated directly from net relative luminescence unit (RLU) measurements using the equation: GSH/GSSG ratio = (Net total glutathione RLUs − Net GSSG RLUs)/(Net GSSG RLUs/2).

### Metabolic flux analysis (Seahorse XF)

Metabolic flux analysis in a Seahorse XF 24 analyzer was used to assess the effect of NNT KD on mitochondrial bioenergetics, applying the Seahorse XF Cell Mito Stress kit (Agilent, Santa Clara, CA). Cells were plated in Seahorse XF microplates the day before the experiment at a density of 100,000 cells per well. Changes in oxygen concentration provide the oxygen consumption rate (OCR), which is a measure of mitochondrial respiration. Changes in proton concentration (or pH) provide the extracellular acidification rate (ECAR), reflective of the rate of glycolysis. Measurements were taken at baseline and after successive application of compounds interfering with oxidative phosphorylation: oligomycin (complex V inhibitor, 2 μM), carbonyl cyanide-*p*-trifluoromethoxyphenylhydrazone (mitochondrial uncoupler, 1 μΜ), and antimycin A plus rotenone (complex I and III inhibition, 1 μΜ). Results were normalized to protein concentration, measured by the BCA protein assay kit (Thermo Fisher).

### Cell proliferation and apoptosis

Cell proliferation was assessed in 96-well plates (loading concentration of 6000 to 8000 cells per well) using the CyQuant proliferation assay kit (Thermo Fisher) and following the manufacturer’s instructions. Cell DNA fluorescence was measured at the end of the time course, that is, 166 hours after siRNA transfection and/or 96 hours after treatment. The beginning of treatment was used as the baseline time point (*t* = 0) for each proliferation series; for siRNA KD experiments, 72 hours posttransfection was taken as the baseline time point. Proliferation rates were provided by the following ratio: (end cell number − baseline cell number)/baseline cell number.

Cellular apoptosis was assessed using the Caspase-Glo 3/7 assay kit (Promega), a luminescence-based assay measuring caspase-3 and caspase-7 activity in cell lysates, and following the manufacturer’s instructions. Luminescent signals were quantified using the Wallac Victor 1420 multilabel counter. At the end of the assay, media and reagents were removed from all wells and stored at −80°C. The next day, relative quantification of cell number was performed by use of the CyQuant proliferation assay kit, as described above. Luminescence values obtained in the caspase assay were normalized to the fluorescence results of the proliferation assay.

Paraquat and auranofin were purchased from Sigma-Aldrich. Buthionine sulfoximine (BSO) was purchased from Cayman Chemical (Ann Arbor, MI).

### *In vitro* steroid profiling by liquid chromatography–tandem mass spectrometry

Steroid synthesis by NCI-H295R cells was assessed by comprehensive multisteroid profiling employing liquid chromatography–tandem mass spectrometry (LC-MS/MS), as described previously (14, 15). Steroid extraction and analysis by LC-MS/MS are discussed in Supplemental Methods.

### RNA sequencing

RNA was prepared in triplicate from NCI-H295R KD siRNA, SCR siRNA (72 hours posttransfection), KD shRNA, and SCR shRNA cells using the RNeasy Mini kit (Qiagen). Libraries were generated using the TruSeq stranded mRNA library prep kit (Illumina, San Diego, CA). A 4 nM library (containing the 16 pooled libraries) was sequenced on a NextSeq 500 system (Illumina). Pathway analyses on sequencing data were completed using the GAGE v2.22 package from Bioconductor release 3.2 and referencing the KEGG pathways. Differentially expressed genes were considered significant applying a false discovery rate of <5% (*q* < 0.05). Differentially regulated pathways were called at a *P* value of <0.01. A detailed description of the methodology for RNA sequencing and pathway analysis can be found in Supplemental Methods.

Additionally, RNA sequencing data from recently published work on three different mouse strains [*Nnt* inactivating mutation, C57BL/6J (RRID: MGI:3702942); wild-type, C57BL/6NHsd (RRID: MGI:2161078); and transgenic *Nnt* overexpressor, C57BL/6J^BAC^] (16) were reanalyzed employing the same pathway analysis as for the human cell–based model; detailed information on this dataset can be found in Supplemental Methods.

### Metabolome analysis

Cell and media samples were prepared for nontargeted metabolome analysis through quenching cell metabolism with a mix of acetonitrile, methanol, and water (Sigma-Aldrich). The process of sample generation and analysis is described in more detail in Supplemental Methods.

### Statistical analysis

Statistical analysis was performed using GraphPad Prism 7 software (RRID: SCR_002798). Data are represented as means ± SEM values, unless otherwise stated. Comparisons were made using a Student paired *t* test for normally distributed data or Wilcoxon signed-rank test for data not following a Gaussian distribution. Multiple comparisons (BSO and auranofin treatments) were performed by one-way ANOVA followed by *post hoc* multiple comparison testing. Statistical methods for the RNA sequencing and untargeted metabolome analysis are detailed in Supplemental Methods.

## Results

### Transient and stable NNT KD

Transient NNT silencing by siRNA KD was employed to explore the acute effects of NNT loss on ACC cells. NNT siRNA transfection in NCI-H295R cells yielded efficient gene silencing for at least 166 hours posttransfection with two different siRNAs (Supplemental Fig. 1A and 1B). All subsequent experiments were performed with the siRNA that gave the best KD results on real-time PCR and Western blotting (referred to here as KD siRNA). The second siRNA (KD siRNA2) was used to corroborate the results of proliferation and apoptosis assays, whose results are most likely to be distorted by off-target effects.

Stable NNT silencing by shRNA KD was used to delineate the long-term effects of NNT loss on ACC cells. Stable NNT KD in NCI-H295R cells was achieved by lentiviral transfection with shRNA-expressing plasmids and selection with puromycin, and resulted in permanent NNT silencing (Supplemental Fig. 1C and 1D).

### NNT siRNA KD increases cellular oxidative stress

**Figure 2.**
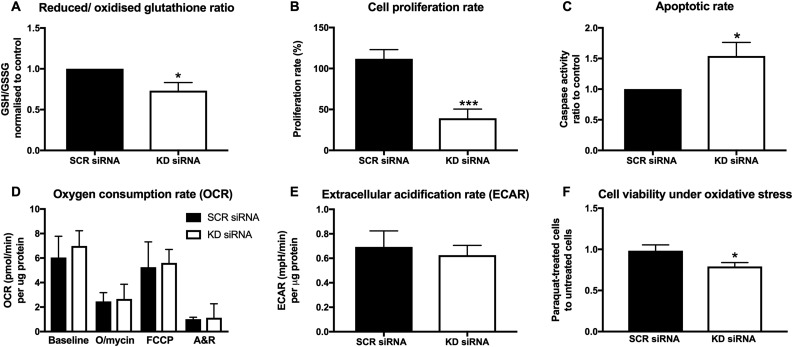
Effects of transient (siRNA-mediated) NNT silencing on NCI-H295R cell redox balance, respiration, proliferation, and viability. Bars represent means ± SEM values, unless stated otherwise. (A) GSH/GSSG ratio in NCI-H295R cells transfected with KD siRNA (96 hours posttransfection), normalized to the corresponding ratio of SCR siRNA-transfected cells. Significant suppression of the GSH/GSSG ratio in KD siRNA cells suggests higher intracellular oxidative stress. Bars represent medians ± interquartile range values. *P < 0.05 (n = 8 independent experiments). (B) Proliferation rates observed in siRNA-transfected NCI-H295R cells, 72 to 166 hours posttransfection. ***P < 0.001 (n = 14). (C) Caspase-3/caspase-7 activity ratio in KD siRNA cells to SCR siRNA-transfected cells, after standardization to cell numbers (120 hours posttransfection). *P < 0.05 (n = 8). (D) Seahorse XF24 analysis of cellular OCR at baseline and after successive application of three mitochondrial respiration inhibitors (166 hours posttransfection). Results were standardized to protein concentration. Bars represent medians ± interquartile range values. *P* > 0.05 (n = 4). (E) ECAR, surrogate marker of anaerobic glycolysis, standardized for protein concentration. *P* > 0.05 (n = 4). (F) Proliferation under low-dose chemically induced oxidative stress (paraquat 10 μM) in KD siRNA and SCR siRNA-transfected cells, normalized to corresponding cell proliferation without paraquat treatment. *P < 0.05 (n = 6). A&R, antimycin A plus rotenone; FCCP, carbonyl cyanide-*p*-trifluoromethoxyphenylhydrazone; O/mycin, oligomycin.

Given the central role of NNT within the mitochondrial ROS scavenging network, we hypothesized that NNT KD will increase oxidative stress in NCI-H295R cells. To test this, we measured the intracellular GSH/GSSG ratio, an established marker of oxidative stress; a decrease in the GSH/GSSG ratio indicates that the proportion of oxidized intracellular glutathione is increased as a result of higher intracellular ROS levels. Indeed, we observed a statistically significant (*P* < 0.05) decrease in the GSH/GSSG ratio in NNT KD siRNA-transfected cells 96 hours posttransfection (Fig. 2A).

### NNT siRNA KD suppresses cell proliferation and induces apoptotic cell death

Cell proliferation rates were assessed during the time window from 72 to 166 hours posttransfection, a period with consistent NNT KD confirmed at the protein level (Supplemental Fig. 1). NNT KD by KD siRNA transfection led to a marked decrease in cellular proliferation rates (Fig. 2B). These results were corroborated by use of a second siRNA against NNT, which completely obliterated cell proliferation (Supplemental Fig. 2).

To establish whether the increased oxidative stress observed with NNT KD leads to higher rates of apoptosis, as predicted by ROS physiology, we measured intracellular caspase-3 and caspase-7 activity 120 hours posttransfection. We also quantified relative cell numbers by DNA fluorescence at the same time point to standardize results to cell number. NNT KD siRNA cells exhibited significantly higher caspase-3/caspase-7 activity than did SCR siRNA cells (*P* < 0.05), confirming our hypothesis that NNT KD triggers cell death by apoptosis (Fig. 2C). The effect was even more marked with the alternative siRNA against NNT (Supplemental Fig. 2).

### NNT siRNA KD sensitizes cells to oxidative stress

Next, we evaluated changes in mitochondrial respiration by direct measurement of the cellular OCR using extracellular flux analysis. Despite the location of NNT in the inner mitochondrial membrane, we observed no statistically significant difference between NNT KD siRNA- and SCR siRNA-transfected cells, either at baseline or in response to mitochondrial respiration disruptors (Fig. 2D). Baseline ECAR, representative of the glycolytic rate, was also similar between the two groups (Fig. 2E).

Considering the integral role of NNT in mitochondrial antioxidant defense and the detrimental impact of NNT inhibition on redox balance, we further hypothesized that NNT loss will render NCI-H295R cells more sensitive to chemically induced oxidative stress. To assess this assumption, we treated NCI-H295R cells with a subtoxic dose of paraquat, a pesticide that induces oxidative stress *in vitro*, generating superoxide. Treatment with 10 μM paraquat for 96 hours led to a statistically significant decrease in cell proliferation in cells transfected with KD siRNA, but not in their counterparts that had been transfected with SCR siRNA (Fig. 2F).

### Redox adaptation develops with stable NNT KD

**Figure 3.**
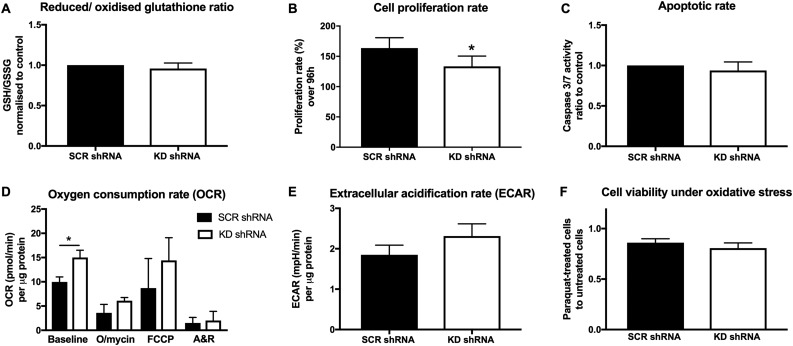
Effects of stable (shRNA-mediated) NNT silencing on NCI-H295R cell redox balance, respiration, proliferation, and viability. Bars represent means ± SEM values, unless stated otherwise. (A) GSH/GSSG ratio in NCI-H295R cells transfected with KD shRNA, normalized to the corresponding ratio cells transfected with SCR shRNA. Bars represent medians ± interquartile range values. *P* > 0.05 (n = 10). (B) Proliferation rates during a 96-hour period. *P < 0.05 (n = 13). (C) Caspase-3/caspase-7 activity ratio in KD shRNA cells to SCR shRNA-transfected cells, after standardization to cell numbers. *P* > 0.05 (n = 4). (D) Seahorse XF24 analysis of cellular OCR at baseline and after successive application of three mitochondrial respiration inhibitors. Results were standardized to protein concentration. Bars represent medians ± interquartile range values. *P < 0.05 (n = 7). (E) ECAR standardized for protein concentration. *P* > 0.05 (n = 7). (F) Proliferation under chemically induced oxidative stress (paraquat) in KD shRNA and SCR shRNA cells, normalized to corresponding cell proliferation without paraquat treatment. *P* > 0.05 (n = 13). A&R, antimycin A plus rotenone; FCCP, carbonyl cyanide-*p*-trifluoromethoxyphenylhydrazone; O/mycin, oligomycin.

To explore the long-term metabolic consequences of NNT silencing in NCI-H295R cells, we employed a different model involving stable transfection with shRNA against NNT. With long-term culture under persistent NNT silencing (4 to 12 weeks posttransfection), cells managed to restore their redox balance to the levels of their SCR shRNA-transfected counterparts (Fig. 3A).

### ACC proliferation remains suppressed with stable NNT KD

The distinct metabolic consequences of NNT silencing in the stable KD setting, in comparison with acute KD by NNT siRNA, translated into an attenuated response with respect to cellular proliferation and viability. Proliferation rates remained significantly lower in KD shRNA-transfected cells compared with the SCR shRNA-transfected controls; however, this effect was less pronounced than the decrease in proliferation we observed with siRNA-mediated KD (Fig. 3B). Apoptotic rates did not differ between SCR shRNA and KD shRNA cells (Fig. 3C), in keeping with the restoration of redox homeostasis we had ascertained based on the GSH/GSSG ratio.

Interestingly, NNT KD shRNA cells consumed more oxygen than did SCR shRNA cells at baseline (Fig. 3D). This finding potentially reflects higher energy needs in NNT-deficient cells. The same trend was observed in ECAR, a surrogate marker of glycolysis, but without reaching statistical significance (Fig. 3E). Finally, stable NNT KD did not enhance cell sensitivity to oxidative stress induced by paraquat (Fig. 3F).

### Transient, but not stable, NNT KD paradoxically stimulates steroidogenesis

**Figure 4.**
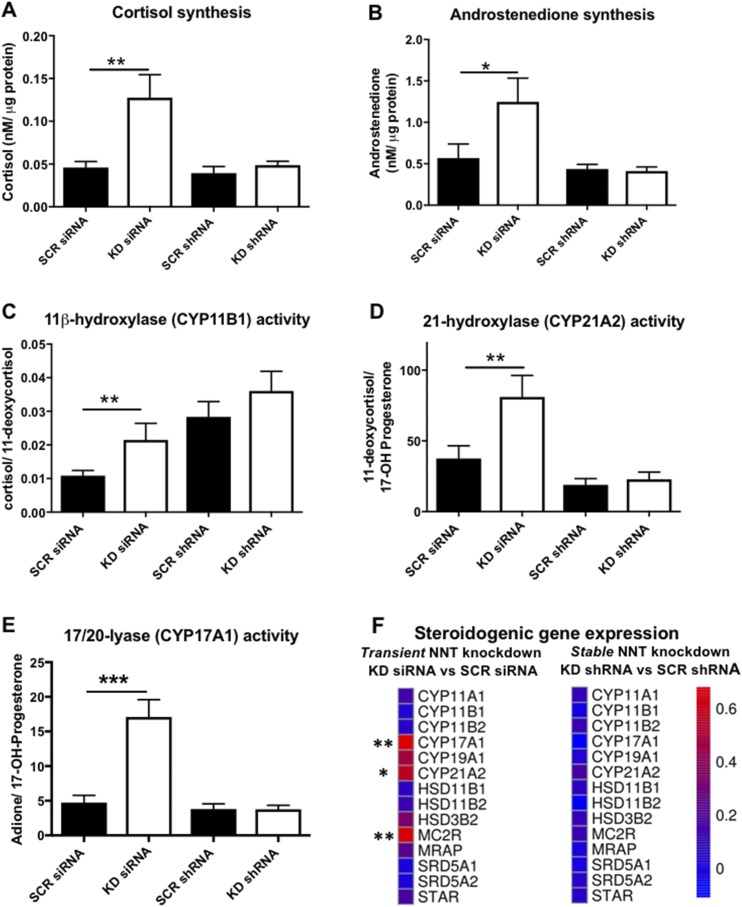
Effects of NNT silencing on NCI-H295R steroidogenesis, delineated by LC-MS/MS steroid profiling in serum-free cell media and RNA sequencing. (A) Cortisol production and (B) androstenedione production during a 48-hour period in NCI-H295R cells transfected with siRNA or shRNA are shown. A significant stimulation of cortisol and androstenedione synthesis was observed 72 to 120 hours posttransfection with KD siRNA. *P < 0.05; **P < 0.01 (n ≥ 5). (C–E) Specific enzyme activity derived from product-to-substrate ratios for (C) 11*β*-hydoxylase (CYP11B1), (D) 21-hydroxylase (CYP21A2), and (E) 17,20-lyase (CYP17A1) in siRNA and shRNA-transfected cells. **P < 0.01; ***P < 0.001 (n ≥ 5). (F) Heat map representation of steroidogenic gene expression changes induced by transient and stable NNT KD, as revealed by RNA sequencing. Scale represents log_2_ fold changes in NNT KD cells compared with their respective (siRNA or shRNA) SCR controls. *q < 0.05; **q < 0.01 (n = 3).

The effects of NNT silencing on steroidogenesis were evaluated by comprehensive multisteroid profiling in cell media by LC-MS/MS, as well as gene expression analysis by qRT-PCR. We postulated that NNT silencing will disrupt steroidogenesis, either depriving mitochondrial steroidogenic monooxygenases [cholesterol side-chain cleaving enzyme (CYP11A1), 11*β*-hydroxylase (CYP11B1), aldosterone synthase (CYP11B2)] of their essential electron donor NADPH, or due to oxidative stress–induced downregulation of key steroidogenic enzymes. Surprisingly, NNT KD siRNA-transfected cells actually produced significantly more glucocorticoids (cortisol) and androgens (androstenedione) than did controls (Fig. 4A and 4B). Individual enzyme activities were determined as product-to-substrate ratios for three key steroidogenic enzymes, 11*β*-hydroxylase (CYP11B1), 21-hydroxylase (CY21A2), and CYP17A1 17/20-lyase activity; all three displayed higher activity in NNT KD siRNA-transfected cells, in keeping with a paradoxical generalized stimulation of steroidogenesis by acute NNT loss (Fig. 4C–4E).

In contrast, in the shRNA-transfected cells with chronic NNT silencing we observed no significant impact on steroidogenesis, with similar rates of cortisol or androstenedione synthesis between KD shRNA and SCR shRNA cells (Fig. 4A–4E).

We also explored the gene expression alterations underpinning the enhanced steroid production of cells transfected with KD siRNA, comparing the expression of core steroidogenic genes (StAR, CYP11A1, CYP21A2, CYP17A1, 3*β*HSD2) between KD siRNA and SCR siRNA cells by qRT-PCR. There was a statistically significant increase in the expression of cytochrome P450 (CYP) type 2 steroidogenic enzymes located in the endoplasmic reticulum (ER) CYP21A2 (*P* < 0.05), CYP17A1 (*P* < 0.05), as well as the ER dehydrogenase HSD3B2 (*P* < 0.01) in NNT KD siRNA cells (Supplemental Table 2). CYP11B1 and CYP11B2 expression levels were too low to be quantified by qRT-PCR in our cells.

Analysis of gene expression by RNA sequencing in an extended panel of 14 steroidogenic genes indicated a significant upregulation of CYP21A2 (*q* < 0.05) and CYP17A1 (*q* < 0.01), as well as the ACTH receptor MC2R (*q* < 0.01), in the transient NNT KD model; no significant changes were observed in the stable KD model (Fig. 4F).

### NCI-H295R cells are sensitive to glutathione depletion and thioredoxin reductase inhibition

Given the effects of NNT inhibition on NCI-H295R cell proliferation, we went on to evaluate the sensitivity of ACC cells to isolated inhibition of each of the two pillars of mitochondrial antioxidant defense: the glutathione pathway and the thioredoxin pathway.

**Figure 5.**
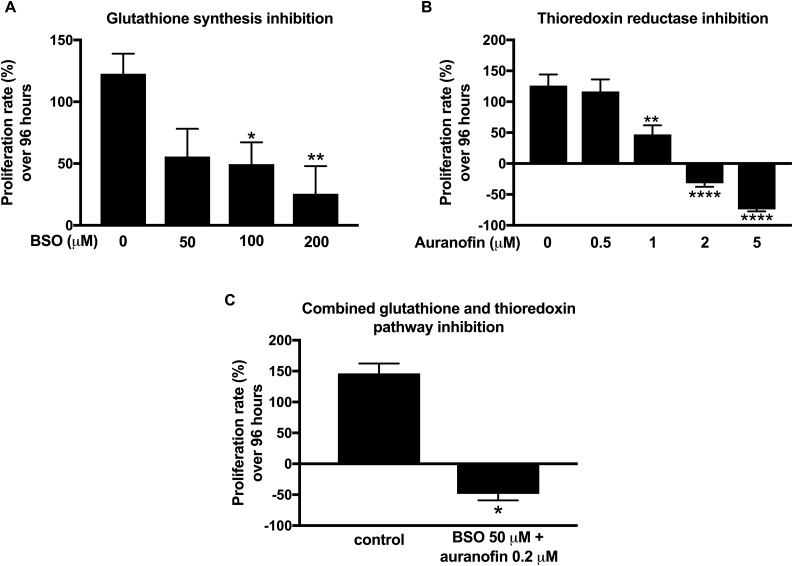
Effect of glutathione and thioredoxin pathway inhibition on NCI-H295R cell proliferation. (A) Ninety-six–hour treatment with incremental doses of BSO (0 to 200 μM), an inhibitor of glutathione synthesis. Control cells were treated with vehicle only. *P < 0.05 (n = 9). (B) Ninety-six–hour treatment with incremental doses of auranofin (0 to 5 μM), a thioredoxin reductase inhibitor. Control cells were treated with vehicle only. Negative proliferation rates indicate net decrease in cell number after 96 hours of treatment. **P < 0.01; ****P < 0.0001 (n = 9). (C) Combined glutathione and thioredoxin inhibition by use of low-dose BSO (50 μM) and auranofin (0.2 μM). *P < 0.05 (n = 3).

We used BSO, a potent inhibitor of the glutathione-producing enzyme c-glutamylcysteine ligase, to deplete intracellular glutathione. We observed a decline in cell proliferation with a BSO dose of ≥100 μM after 96 hours of treatment (Fig. 5A).

Pharmacological manipulation of the alternative mitochondrial antioxidant pathway, the thioredoxin pathway, was achieved by auranofin, a gold complex agent with a well-established capacity to inhibit thioredoxin reductase. NCI-H295R treatment with doses of ≥1 μM was associated with major cytotoxicity (Fig. 5B).

Finally, dual inhibition of the glutathione and thioredoxin pathways by coadministration of low-dose BSO (50 μM) and auranofin (0.2 μM) resulted in marked cytotoxicity, suggesting that a potent synergistic effect can be achieved by dual pathway targeting (Fig. 5C).

### Whole-transcriptome and metabolome analyses reveal extensive metabolic perturbations with transient NNT KD, as well as changes in protein processing and polyamine metabolism with stable NNT KD

To uncover the molecular mechanisms that underpin the effects we observed in the two models and the discrepancies between them, we applied whole-transcriptome analysis in RNA extracted from four groups of NCI-H295R cells (NNT KD siRNA vs SCR siRNA, NNT KD shRNA vs SCR shRNA). Eight hundred forty-two genes were differentially regulated between NNT KD siRNA and SCR siRNA cells; 247 genes were differentially regulated between NNT KD shRNA and SCR shRNA cells (*q* < 0.05). Of note, only 17 of the genes regulated differentially between KD and SCR cells were identical in the two models, NNT KD siRNA and shRNA (Supplemental Table 3).

**Figure 6.**
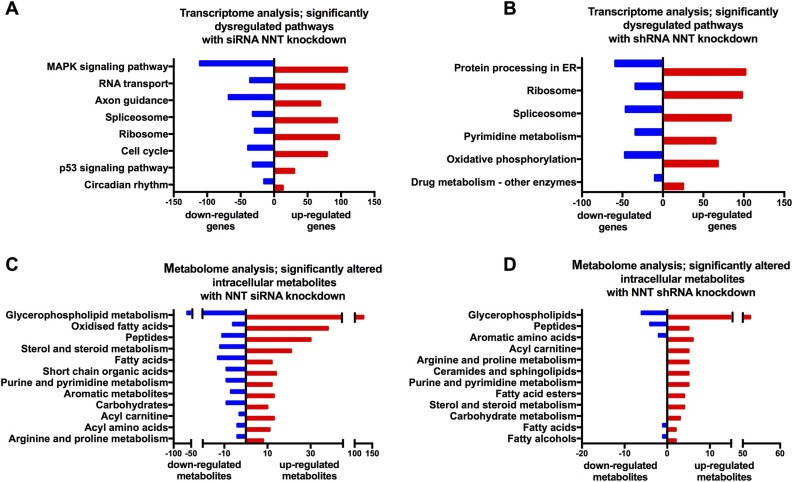
Whole-transcriptome sequencing and nontargeted metabolome analysis of KD siRNA, SCR siRNA, KD shRNA, and SCR shRNA-transfected NCI-H295R cells. (A and B) Whole-transcriptome sequencing in siRNA and shRNA-transfected NCI-H295R cells. (A) Significantly altered molecular pathways (*P* < 0.01, *q* < 0.05) between KD siRNA and SCR siRNA cells and number of associated genes that are upregulated/downregulated in KD siRNA cells. (B) Significantly altered molecular pathways (*P* < 0.01) between KD shRNA and SCR shRNA cells and number of associated genes that are upregulated/downregulated in KD shRNA cells. (C and D) Nontargeted metabolome analysis in siRNA and shRNA-transfected NCI-H295R cells. (C) Significantly upregulated and downregulated intracellular metabolites (*q* < 0.01) in KD siRNA-transfected cells, as compared with SCR siRNA-transfected cells. (D) Significantly upregulated and downregulated intracellular metabolites (*P* < 0.05) in KD shRNA-transfected cells, as compared with SCR shRNA-transfected cells (no metabolites with *q* < 0.05 in this comparison).

Differentially regulated pathways are shown in Fig. 6A and 6B (*P* < 0.01) and tabulated in Supplemental Table 4. In NNT KD siRNA cells, significant changes were observed in crucial pathways affecting cellular proliferation and viability (p53 pathway, mitogen-activated signaling kinase pathway, checkpoint kinases). Interestingly, in KD shRNA cells one of the borderline significantly altered pathways (*P* < 0.01, *q* = 0.11) controlled protein processing in the ER, with upregulation of genes encoding heat shock proteins (predominantly in the HSP40 family), chaperone proteins that facilitate correct protein folding, and transfer of misfolded proteins to proteasomes for degradation (Supplemental Table 4) (17, 18). Other significantly upregulated pathways with stable NNT KD included ribosomal genes (*P* < 0.01, *q* < 0.05) and pyrimidine metabolism (*P* < 0.01, *q* = 0.11), including an upregulation of RNA II polymerases. Taken together, these findings hint at a higher protein turnover that may allow cells to swiftly replace proteins that have sustained irreversible oxidative damage.

Of note, recent rodent-based work by Meimaridou *et al.* (16) performed RNA sequencing on mouse adrenal glands derived from three different mouse strains: a strain that carries an inactivating mutation of *Nnt* (C57BL/6J), a strain with wild-type *Nnt* expression (C57BL/6N), and transgenic mice overexpressing *Nnt* on the background of the NNT-deficient mouse strain (rescue model; C57BL/6J^BAC^). We have extended this work in the present study, carrying out additional pathway analyses on these data. Detailed information on significantly dysregulated pathways in C57BL/6J vs C57BL/6N mice and C57BL/6J vs C57BL/6J^BAC^ mice are presented in Supplemental Table 5. Significant dysregulation of the major cell signaling pathway of mitogen-activated signaling kinases was one of the salient molecular changes in both comparisons; the same pathway was also dysregulated with transient NNT KD in NCI-H295R cells. Oxidative phosphorylation was upregulated in both comparisons, but changes were much more pronounced in the C57BL/6J vs C57BL/6N comparison and likely to reflect a strain, rather than gene, effect.

RNA sequencing in NCI-H295R cells was complemented by whole-metabolome analysis performed separately in cells and corresponding cell culture supernatant. In the siRNA KD model, NNT silencing was associated with a significant metabolic perturbation when compared with the SCR siRNA cells. An increase in the presence of 44 oxidized fatty acids supports a shift to a more oxidized intracellular microenvironment (Fig. 6C; Supplemental Tables 6 and 7). This was not observed in the stable NNT KD model. Pathway enrichment analysis demonstrated that six important metabolic pathways were enriched (*q* < 0.05): TCA cycle, arginine and proline metabolism, pyrimidine metabolism, nicotinate and nicotinamide metabolism, and glutathione metabolism. Beyond this, we observed statistically significant changes (*q* < 0.01) for 16 acyl carnitines, 25 fatty acids, 15 acyl amino acids, 20 purine and pyrimidine metabolites, and 5 metabolites present in the nicotinate and nicotinamide metabolic pathways. Taken together, these findings indicate a perturbation in mitochondrial fatty acid *β*-oxidation (as shown by changes in fatty acids, acyl carnitines, and TCA metabolites), changes in nucleotide synthesis, and a potential overload of acetyl units.

In the stable NNT shRNA KD model, pathway enrichment analysis highlighted changes in purine metabolism (*q* < 0.05), and NNT KD cells exhibited a significant (*P* < 0.05) rise in several purine and pyrimidine metabolites (Fig. 6D; Supplemental Tables 6 and 7), mirroring the results of the transcriptome analysis. Polyamine (spermine, spermidine) metabolism was also significantly modified in both models: KD siRNA cells displayed a dramatic increase in polyamine catabolism (accumulation of diacetyl-spermine, diacetyl-spermidine, spermine dialdehyde), leading to depletion of spermine and spermidine, a response that has been associated with arrest of cell growth (Supplemental Tables 6 and 7) (19). Conversely, KD shRNA cells exhibited a significant rise in spermine, a polyamine that can act as an ROS scavenger (20, 21).

## Discussion

In this study, we have explored the immediate and longer term impact of NNT silencing on ACC cells with respect to redox balance, mitochondrial bioenergetics, cell proliferation and viability, and steroidogenesis, using two distinct *in vitro* KD models in the human ACC cell line NCI-H295R. Our aim was to establish whether NNT inhibition can have therapeutically beneficial effects with respect to control of tumor growth and steroid excess. We hypothesized that NNT inhibition would compromise the ability of adrenocortical mitochondria to deal with oxidative stress, leading to progressive accumulation of ROS. ROS excess has multiple toxic sequelae and can directly impair cell viability, triggering apoptosis (8, 13). Importantly, the adrenal-specific clinical phenotype in humans and the reported increased rate of adrenocortical cell apoptosis in otherwise healthy NNT mutant mice suggest that this manipulation may selectively target ACC cells, sparing other organs. This susceptibility of adrenocortical cells to mitochondrial antioxidant pathway disruption can be explained by the fact that enzymes involved in the rapid adrenal steroid response to stress represent a major additional source of ROS in the adrenals, increasing their dependence on efficient ROS scavenging (10, 11).

In keeping with our hypothesis, we found that in the acute setting (siRNA-mediated transient KD), NNT loss increased intracellular oxidative stress. Redox balance perturbations in response to NNT loss have been previously demonstrated in a limited number of cell lines *in vitro*, as well as in lymphocytes derived from NNT mutant patients *ex vivo* (4, 22–25). These findings are in line with the biological role of NNT as a major mitochondrial generator of NADPH, the essential provider of reducing equivalents to the two main antioxidant pathways (26).

Importantly, in our study we observed that NNT silencing led to an immediate and marked inhibition of cell proliferation accompanied by increased apoptotic rates. This antitumor effect was even more pronounced when using a second anti-NNT siRNA (KD siRNA2). This apparent difference in degree (but not in direction) of cell response raised the possibility of additional, off-target effects triggered by KD siRNA2; on interrogation of the National Center for Biotechnology Information Basic Local Assignment Search Tool, however, neither of the two siRNAs shares substantial homology with any genes that would be expected to impact cell proliferation and viability. The association between excessive oxidative stress and mitochondrial apoptosis has been well established in the literature (8, 13), but data on the effects of NNT loss on cellular proliferation and viability are limited. Transient NNT silencing was previously shown to increase rates of apoptosis in PC12 (rat pheochromocytoma) cells (22); stable NNT KD in human melanoma cells was associated with reduced viability and high apoptotic rates *in vitro*, as well as slower growth of melanoma xenografts in mice (27). Meimaridou *et al.* (4) reported high levels of apoptosis in the zona fasciculata of the adrenal cortex from NNT mutant mice, as well as NCI-H295R cells stably transfected with shRNA against NNT *in vitro*. Although ROS have typically been associated with a stimulation of cellular proliferation, a number of *in vitro* models have demonstrated the opposite effect (suppression of cell division), in a complex relationship that may depend on the magnitude of ROS excess and/or tissue type (28, 29). NNT inhibition may also interfere with cellular proliferation in a ROS-independent way, curtailing the amount of NADPH available to fuel the pressing anabolic needs of malignant cells. In keeping with the major impact on cellular viability and proliferation, we observed far-reaching metabolic effects of NNT KD implicating several areas of cell metabolism, including mitochondrial fatty acid oxidation, polyamine metabolism, and nucleotide synthesis. The enhanced cellular sensitivity to oxidative stress in the aftermath of NNT silencing (paraquat treatment) is translationally important, as oxidative stress is induced by a number of classic chemotherapy agents, contributing to their cytotoxic effect (8, 30). NNT inhibition could represent a feasible strategy to sensitize ACC to such drugs.

The longer term effects of NNT loss on ACC cells, as delineated in the stable KD model, were disparate from the ones encountered in the acute setting. Importantly, with long-term culture under constant NNT silencing, NCI-H295R ACC cells managed to restore their redox balance. This compensation abrogated the proapoptotic early impact of NNT loss. Interestingly, a persistent proliferative handicap was demonstrated, although this was less marked than the one observed in the acute setting. This may be attributable to the limited supply of NADPH in the absence of NNT. Extracellular flux analysis revealed higher rates of oxygen consumption in KD shRNA cells, a response that may reflect higher energy needs or be driven by the spare NADH that fails to be converted to NADPH in NNT-deficient cells. Previous studies on the effect of NNT silencing on oxygen consumption have shown mixed results, which may be cell type–dependent (22, 25, 31).

Redox adaptation to oxidative stress has been previously described in tumor models *in vitro*; this process is driven by the strong selective pressure applied by oxidative toxicity and promoted by the genomic instability that characterizes the oxidized intracellular microenvironment (32). We obtained insights into how this adaptation was facilitated in our model by comprehensive transcriptome and metabolome analysis. In NNT KD shRNA cells, we observed an upregulation of genes that are involved in protein folding in the ER, as well as in the identification and degradation of damaged proteins. Purine and pyrimidine metabolism was activated in these cells, and ribosomal genes were upregulated. Taken together, these findings hint at increased protein turnover, involving degradation of damaged protein and acceleration of new protein synthesis. This may represent a key compensatory mechanism against oxidative stress, achieving the timely removal and replacement of irrevocably damaged (oxidized) proteins. The observed increase in oxygen consumption could provide additional energy to fuel this process. Of note, we recently described upregulation of chaperone proteins in the adrenals of NNT-deficient mice (16). The additional pathway analysis we performed on the same RNA sequencing data from that rodent work displayed otherwise limited overlap with our *in vitro* model, likely reflecting the expected biological differences between a healthy mouse adrenal and a malignant human adrenal cell line.

Interestingly, polyamine metabolism exhibited dramatic shifts in opposite directions in the two models. Polyamines (spermine, spermidine) are versatile cationic molecules involved in a number of cell processes, including ROS scavenging and cell proliferation (19, 33). High endogenous polyamine levels have been found in a number of cancer types (34). Acute NNT loss was accompanied by a rapid accumulation of acetylated catabolic products of polyamines, leading to depletion of spermine and spermidine. Polyamine catabolism can both be triggered by oxidative stress and generate hydrogen peroxide, creating a vicious cycle that propagates ROS accumulation (33, 35). Indeed, stimulated polyamine catabolism has been associated with growth arrest and cell death in various *in vitro* models (19, 36). Conversely, in the chronic setting, stable NNT KD cells demonstrated increased spermine concentrations and no evidence of accelerated polyamine catabolism. This response is likely to represent a major facilitator of the successful redox adaptation in this model. Our findings underscore the importance of polyamine homeostasis in ACC cells.

Within the same framework, we also explored alternative antioxidant targets focusing on the glutathione and thioredoxin pathways. Pertinently, human mutations in thioredoxin reductase 2 have also been shown to result in isolated glucocorticoid deficiency (37). We used BSO to inhibit glutathione synthesis. BSO has shown antiproliferative effects against a number of cell lines *in vitro* (38–43). We observed a significant suppression of cell growth with doses of ≥100 μM, that is, at doses that are clinically attainable in plasma with no serious toxicity (38). Auranofin, a gold complex–based agent able to inhibit thioredoxin reductase, also suppressed cell proliferation at doses ≥1 μM and was associated with marked cytotoxicity at doses of ≥2 μM. Auranofin has also displayed antitumor activity against a number of cell lines *in vitro* and is currently being investigated in clinical trials against leukemia (44–47). Applying combined treatment with low doses of both agents, we observed a dramatic cytotoxic impact, suggesting that dual antioxidant targeting can achieve potent synergistic results.

The observed effects of NNT silencing on NCI-H295R steroidogenesis were surprising. In the acute setting, that is, NNT siRNA KD, we observed a generalized stimulation of steroidogenesis, leading to increased glucocorticoid and adrenal androgen output by the cells. This was corroborated by a significant upregulation of a number of steroidogenic enzymes. This response is contrary to what one might have anticipated considering that mitochondrial NADPH is an essential cofactor to the steroidogenic cytochrome P450 enzymes CYP11A1, CYP11B1, and CYP11B2. Elucidating the mechanisms that drive this transient effect will require additional studies. The few studies exploring the relationship between ROS and steroidogenesis (mostly on testicular Leydig cell tumor cells) have reported a downregulation of steroidogenic enzymes with oxidative stress (14, 48–50). Human patients with inactivating NNT mutations (4) and a murine *Nnt* deletion model (16) have been shown to have disrupted steroidogenesis; the data from our *in vitro* NNT KD models suggest that NNT loss is not limiting for adrenal steroidogenesis. Interestingly, Zhao *et al.* (51) demonstrated a biphasic relationship between ROS and steroidogenesis, indicating that the direction of the effect is dose-dependent.

**Figure 7.**
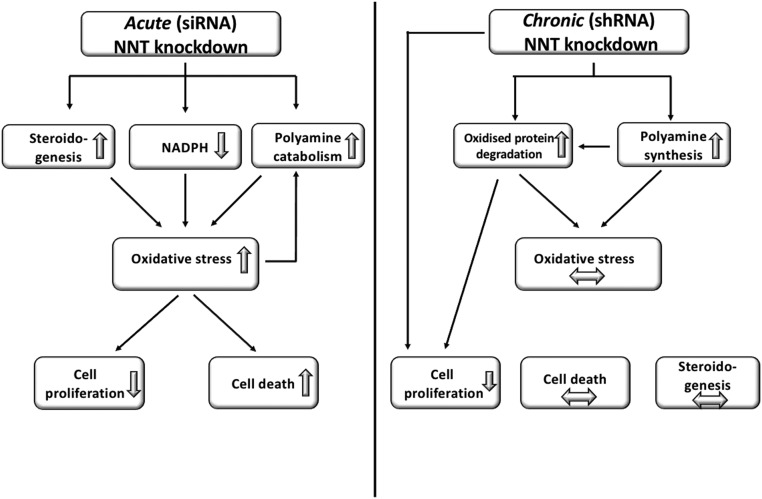
Response of NCI-H295R cells to NNT silencing in the acute (transient KD) and chronic (stable KD) setting, with proposed redox adaptation mechanisms. Acute NNT KD induces oxidative stress as predicted by the role of NNT as a major NADPH generator; enhanced steroidogenesis and polyamine catabolism further accentuate ROS accumulation, triggering apoptosis and a sharp decline in cell proliferation. With time (stable KD), cells manage to adapt removing damaged proteins and enhancing spermine synthesis as an alternative, NADPH-independent ROS scavenger. This restores redox homeostasis and abrogates the original proapoptotic effect, but cellular proliferation remains suppressed. Horizontal arrows represent paucity of change.

Taken together, we show that NNT silencing can induce cytotoxicity and impede cell growth in ACC cells, as well as sensitize them to chemically induced oxidative stress. Moreover, we have demonstrated how the plasticity of ACC cells can lead to the development of a compensatory molecular response with time and described how changes in polyamine metabolism and ER protein processing are involved in this process (Fig. 7). A limitation of our work is that it is based on a single cell line; however, NCI-H295R remains the only established, well-characterized steroidogenic human ACC cell line. These results merit further exploration with *in vivo* studies to corroborate the effectiveness of mitochondrial antioxidant pathway targeting and explore its durability, alone or in combination with other pro-oxidant agents. The unique features of adrenocortical cells, with their high-volume ROS generation due to steroidogenesis, make ACC a most amenable target to this approach.

## Supplementary Material

Supplemental Data

## Abbreviations:

ACC: adrenocortical carcinoma
BSO: buthionine sulfoximine
ECAR: extracellular acidification rate
ER: endoplasmic reticulum
GSH: reduced glutathione
GSSG: oxidized glutathione
KD: knockdown
LC-MS/MS: liquid chromatography–tandem mass spectrometry
NADH: reduced form of NAD
NADPH: reduced form of NAD phosphate
NNT: nicotinamide nucleotide transhydrogenase
OCR: oxygen consumption rate
qRT-PCR: quantitative real-time PCR
RLU: relative luminescence unit
ROS: reactive oxygen species
SCR: scrambled
shRNA: short hairpin RNA
siRNA: small interfering RNA
TCA: tricarboxylic acid

## References

[1] Else T, Kim AC, Sabolch A, Raymond VM, Kandathil A, Caoili EM, Jolly S, Miller BS, Giordano TJ, Hammer GD Adrenocortical carcinoma. Endocr Rev. 2014;35(2):282–326. 24423978 10.1210/er.2013-1029 PMC3963263

[2] Fassnacht M, Kroiss M, Allolio B Update in adrenocortical carcinoma. J Clin Endocrinol Metab. 2013;98(12):4551–4564. 24081734 10.1210/jc.2013-3020

[3] Fassnacht M, Terzolo M, Allolio B, Baudin E, Haak H, Berruti A, Welin S, Schade-Brittinger C, Lacroix A, Jarzab B, Sorbye H, Torpy DJ, Stepan V, Schteingart DE, Arlt W, Kroiss M, Leboulleux S, Sperone P, Sundin A, Hermsen I, Hahner S, Willenberg HS, Tabarin A, Quinkler M, de la Fouchardière C, Schlumberger M, Mantero F, Weismann D, Beuschlein F, Gelderblom H, Wilmink H, Sender M, Edgerly M, Kenn W, Fojo T, Müller HH, Skogseid B; FIRM-ACT Study Group Combination chemotherapy in advanced adrenocortical carcinoma. N Engl J Med. 2012;366(23):2189–2197. 22551107 10.1056/NEJMoa1200966

[4] Meimaridou E, Kowalczyk J, Guasti L, Hughes CR, Wagner F, Frommolt P, Nürnberg P, Mann NP, Banerjee R, Saka HN, Chapple JP, King PJ, Clark AJ, Metherell LA Mutations in NNT encoding nicotinamide nucleotide transhydrogenase cause familial glucocorticoid deficiency. Nat Genet. 2012;44(7):740–742. 22634753 10.1038/ng.2299 PMC3386896

[5] Roucher-Boulez F, Mallet-Motak D, Samara-Boustani D, Jilani H, Ladjouze A, Souchon PF, Simon D, Nivot S, Heinrichs C, Ronze M, Bertagna X, Groisne L, Leheup B, Naud-Saudreau C, Blondin G, Lefevre C, Lemarchand L, Morel Y NNT mutations: a cause of primary adrenal insufficiency, oxidative stress and extra-adrenal defects. Eur J Endocrinol. 2016;175(1):73–84. 27129361 10.1530/EJE-16-0056

[6] Arkblad EL, Betsholtz C, Mandoli D, Rydström J Characterization of a nicotinamide nucleotide transhydrogenase gene from the green alga *Acetabularia acetabulum* and comparison of its structure with those of the corresponding genes in mouse and *Caenorhabditis elegans*. Biochim Biophys Acta. 2001;1520(2):115–123. 11513952 10.1016/s0167-4781(01)00257-3

[7] Sabharwal SS, Schumacker PT Mitochondrial ROS in cancer: initiators, amplifiers or an Achilles’ heel? Nat Rev Cancer. 2014;14(11):709–721. 25342630 10.1038/nrc3803 PMC4657553

[8] Gupta SC, Hevia D, Patchva S, Park B, Koh W, Aggarwal BB Upsides and downsides of reactive oxygen species for cancer: the roles of reactive oxygen species in tumorigenesis, prevention, and therapy. Antioxid Redox Signal. 2012;16(11):1295–1322. 22117137 10.1089/ars.2011.4414 PMC3324815

[9] Gupta A, Bhatt ML, Misra MK Assessment of free radical-mediated damage in head and neck squamous cell carcinoma patients and after treatment with radiotherapy. Indian J Biochem Biophys. 2010;47(2):96–99. 20521622

[10] Hanukoglu I, Hanukoglu Z Stoichiometry of mitochondrial cytochromes *P*-450, adrenodoxin and adrenodoxin reductase in adrenal cortex and corpus luteum. Implications for membrane organization and gene regulation. Eur J Biochem. 1986;157(1):27–31. 3011431 10.1111/j.1432-1033.1986.tb09633.x

[11] Prasad R, Kowalczyk JC, Meimaridou E, Storr HL, Metherell LA Oxidative stress and adrenocortical insufficiency. J Endocrinol. 2014;221(3):R63–R73. 24623797 10.1530/JOE-13-0346 PMC4045218

[12] Giordano TJ, Kuick R, Else T, Gauger PG, Vinco M, Bauersfeld J, Sanders D, Thomas DG, Doherty G, Hammer G Molecular classification and prognostication of adrenocortical tumors by transcriptome profiling. Clin Cancer Res. 2009;15(2):668–676. 19147773 10.1158/1078-0432.CCR-08-1067 PMC2629378

[13] Fruehauf JP, Meyskens FL Jr Reactive oxygen species: a breath of life or death? Clin Cancer Res. 2007;13(3):789–794. 17289868 10.1158/1078-0432.CCR-06-2082

[14] Jühlen R, Idkowiak J, Taylor AE, Kind B, Arlt W, Huebner A, Koehler K Role of ALADIN in human adrenocortical cells for oxidative stress response and steroidogenesis. PLoS One. 2015;10(4):e0124582. 25867024 10.1371/journal.pone.0124582 PMC4395102

[15] Lebbe M, Taylor AE, Visser JA, Kirkman-Brown JC, Woodruff TK, Arlt W The steroid metabolome in the isolated ovarian follicle and its response to androgen exposure and antagonism. Endocrinology. 2017;158(5):1474–1485. 28323936 10.1210/en.2016-1851 PMC5460835

[16] Meimaridou E, Goldsworthy M, Chortis V, Fragouli E, Foster PA, Arlt W, Cox R, Metherall LA NNT is a key regulator of adrenal redox homeostasis and steroidogenesis in male mice. J Endocrinol. 2017;236(1):13–28. 29046340 10.1530/JOE-16-0638 PMC5744559

[17] Schröder M, Kaufman RJ The mammalian unfolded protein response. Annu Rev Biochem. 2005;74(1):739–789. 15952902 10.1146/annurev.biochem.73.011303.074134

[18] Tameire F, Verginadis II, Koumenis C Cell intrinsic and extrinsic activators of the unfolded protein response in cancer: mechanisms and targets for therapy. Semin Cancer Biol. 2015;33:3–15. 25920797 10.1016/j.semcancer.2015.04.002 PMC4523493

[19] Mandal S, Mandal A, Johansson HE, Orjalo AV, Park MH Depletion of cellular polyamines, spermidine and spermine, causes a total arrest in translation and growth in mammalian cells. Proc Natl Acad Sci USA. 2013;110(6):2169–2174. 23345430 10.1073/pnas.1219002110 PMC3568356

[20] Wei C, Wang Y, Li M, Li H, Lu X, Shao H, Xu C Spermine inhibits endoplasmic reticulum stress–induced apoptosis: a new strategy to prevent cardiomyocyte apoptosis. Cell Physiol Biochem. 2016;38(2):531–544. 26828926 10.1159/000438648

[21] Sava IG, Battaglia V, Rossi CA, Salvi M, Toninello A Free radical scavenging action of the natural polyamine spermine in rat liver mitochondria. Free Radic Biol Med. 2006;41(8):1272–1281. 17015174 10.1016/j.freeradbiomed.2006.07.008

[22] Yin F, Sancheti H, Cadenas E Silencing of nicotinamide nucleotide transhydrogenase impairs cellular redox homeostasis and energy metabolism in PC12 cells. Biochim Biophys Acta. 2012;1817(3):401–409. 22198343 10.1016/j.bbabio.2011.12.004 PMC3269566

[23] Lopert P, Patel M Nicotinamide nucleotide transhydrogenase (Nnt) links the substrate requirement in brain mitochondria for hydrogen peroxide removal to the thioredoxin/peroxiredoxin (Trx/Prx) system. J Biol Chem. 2014;289(22):15611–15620. 24722990 10.1074/jbc.M113.533653 PMC4140916

[24] Ronchi JA, Francisco A, Passos LA, Figueira TR, Castilho RF The contribution of nicotinamide nucleotide transhydrogenase to peroxide detoxification is dependent on the respiratory state and counterbalanced by other sources of NADPH in liver mitochondria. J Biol Chem. 2016;291(38):20173–20187. 27474736 10.1074/jbc.M116.730473 PMC5025700

[25] Fujisawa Y, Napoli E, Wong S, Song G, Yamaguchi R, Matsui T, Nagasaki K, Ogata T, Giulivi C Impact of a novel homozygous mutation in nicotinamide nucleotide transhydrogenase on mitochondrial DNA integrity in a case of familial glucocorticoid deficiency. BBA Clin. 2015;3:70–78. 26309815 10.1016/j.bbacli.2014.12.003 PMC4545511

[26] Arkblad EL, Tuck S, Pestov NB, Dmitriev RI, Kostina MB, Stenvall J, Tranberg M, Rydström J A *Caenorhabditis elegans* mutant lacking functional nicotinamide nucleotide transhydrogenase displays increased sensitivity to oxidative stress. Free Radic Biol Med. 2005;38(11):1518–1525. 15890626 10.1016/j.freeradbiomed.2005.02.012

[27] Gameiro PA, Laviolette LA, Kelleher JK, Iliopoulos O, Stephanopoulos G Cofactor balance by nicotinamide nucleotide transhydrogenase (NNT) coordinates reductive carboxylation and glucose catabolism in the tricarboxylic acid (TCA) cycle. J Biol Chem. 2013;288(18):12967–12977. 23504317 10.1074/jbc.M112.396796 PMC3642339

[28] Koka PS, Mondal D, Schultz M, Abdel-Mageed AB, Agrawal KC Studies on molecular mechanisms of growth inhibitory effects of thymoquinone against prostate cancer cells: role of reactive oxygen species. Exp Biol Med (Maywood). 2010;235(6):751–760. 20511679 10.1258/ebm.2010.009369

[29] Donadelli M, Costanzo C, Beghelli S, Scupoli MT, Dandrea M, Bonora A, Piacentini P, Budillon A, Caraglia M, Scarpa A, Palmieri M Synergistic inhibition of pancreatic adenocarcinoma cell growth by trichostatin A and gemcitabine. Biochim Biophys Acta. 2007;1773(7):1095–1106. 17555830 10.1016/j.bbamcr.2007.05.002

[30] Montero AJ, Jassem J Cellular redox pathways as a therapeutic target in the treatment of cancer. Drugs. 2011;71(11):1385–1396. 21812504 10.2165/11592590-000000000-00000

[31] Ho HY, Lin YT, Lin G, Wu PR, Cheng ML Nicotinamide nucleotide transhydrogenase (NNT) deficiency dysregulates mitochondrial retrograde signaling and impedes proliferation. Redox Biol. 2017;12:916–928. 28478381 10.1016/j.redox.2017.04.035 PMC5426036

[32] Trachootham D, Alexandre J, Huang P Targeting cancer cells by ROS-mediated mechanisms: a radical therapeutic approach? Nat Rev Drug Discov. 2009;8(7):579–591. 19478820 10.1038/nrd2803

[33] Pirinen E, Kuulasmaa T, Pietilä M, Heikkinen S, Tusa M, Itkonen P, Boman S, Skommer J, Virkamäki A, Hohtola E, Kettunen M, Fatrai S, Kansanen E, Koota S, Niiranen K, Parkkinen J, Levonen AL, Ylä-Herttuala S, Hiltunen JK, Alhonen L, Smith U, Jänne J, Laakso M Enhanced polyamine catabolism alters homeostatic control of white adipose tissue mass, energy expenditure, and glucose metabolism. Mol Cell Biol. 2007;27(13):4953–4967. 17485446 10.1128/MCB.02034-06 PMC1951486

[34] Gerner EW, Meyskens FL Jr Polyamines and cancer: old molecules, new understanding. Nat Rev Cancer. 2004;4(10):781–792. 15510159 10.1038/nrc1454

[35] Chopra S, Wallace HM Hydrogen peroxide induces the catabolism of polyamines in human breast cancer cells. Biochem Soc Trans. 1996;24(2):230S. 8736888 10.1042/bst024230s

[36] Babbar N, Ignatenko NA, Casero RA Jr, Gerner EW Cyclooxygenase-independent induction of apoptosis by sulindac sulfone is mediated by polyamines in colon cancer. J Biol Chem. 2003;278(48):47762–47775. 14506281 10.1074/jbc.M307265200

[37] Prasad R, Chan LF, Hughes CR, Kaski JP, Kowalczyk JC, Savage MO, Peters CJ, Nathwani N, Clark AJ, Storr HL, Metherell LA Thioredoxin reductase 2 (TXNRD2) mutation associated with familial glucocorticoid deficiency (FGD). J Clin Endocrinol Metab. 2014;99(8):E1556–E1563. 24601690 10.1210/jc.2013-3844 PMC4207928

[38] Bailey HH l-*S*,*R*-buthionine sulfoximine: historical development and clinical issues. Chem Biol Interact. 1998;111–112:239–254. 10.1016/s0009-2797(97)00164-6 9679558

[39] Bailey HH, Mulcahy RT, Tutsch KD, Arzoomanian RZ, Alberti D, Tombes MB, Wilding G, Pomplun M, Spriggs DR Phase I clinical trial of intravenous l-buthionine sulfoximine and melphalan: an attempt at modulation of glutathione. J Clin Oncol. 1994;12(1):194–205. 8270977 10.1200/JCO.1994.12.1.194

[40] Lee HR, Cho JM, Shin DH, Yong CS, Choi HG, Wakabayashi N, Kwak MK Adaptive response to GSH depletion and resistance to l-buthionine-(*S*,*R*)-sulfoximine: involvement of Nrf2 activation. Mol Cell Biochem. 2008;318(1–2):23–31. 18587629 10.1007/s11010-008-9853-y

[41] Maeda H, Hori S, Ohizumi H, Segawa T, Kakehi Y, Ogawa O, Kakizuka A Effective treatment of advanced solid tumors by the combination of arsenic trioxide and l-buthionine-sulfoximine. Cell Death Differ. 2004;11(7):737–746. 15002036 10.1038/sj.cdd.4401389

[42] Schnelldorfer T, Gansauge S, Gansauge F, Schlosser S, Beger HG, Nussler AK Glutathione depletion causes cell growth inhibition and enhanced apoptosis in pancreatic cancer cells. Cancer. 2000;89(7):1440–1447. 11013356

[43] Rudin CM, Yang Z, Schumaker LM, VanderWeele DJ, Newkirk K, Egorin MJ, Zuhowski EG, Cullen KJ Inhibition of glutathione synthesis reverses Bcl-2-mediated cisplatin resistance. Cancer Res. 2003;63(2):312–318. 12543781

[44] Gandin V, Fernandes AP, Rigobello MP, Dani B, Sorrentino F, Tisato F, Björnstedt M, Bindoli A, Sturaro A, Rella R, Marzano C Cancer cell death induced by phosphine gold(I) compounds targeting thioredoxin reductase. Biochem Pharmacol. 2010;79(2):90–101. 19665452 10.1016/j.bcp.2009.07.023

[45] Li H, Hu J, Wu S, Wang L, Cao X, Zhang X, Dai B, Cao M, Shao R, Zhang R, Majidi M, Ji L, Heymach JV, Wang M, Pan S, Minna J, Mehran RJ, Swisher SG, Roth JA, Fang B Auranofin-mediated inhibition of PI3K/AKT/mTOR axis and anticancer activity in non-small cell lung cancer cells. Oncotarget. 2016;7(3):3548–3558. 26657290 10.18632/oncotarget.6516 PMC4823126

[46] Sobhakumari A, Love-Homan L, Fletcher EV, Martin SM, Parsons AD, Spitz DR, Knudson CM, Simons AL Susceptibility of human head and neck cancer cells to combined inhibition of glutathione and thioredoxin metabolism. PLoS One. 2012;7(10):e48175. 23118946 10.1371/journal.pone.0048175 PMC3485193

[47] Weir SJ, DeGennaro LJ, Austin CP Repurposing approved and abandoned drugs for the treatment and prevention of cancer through public-private partnership. Cancer Res. 2012;72(5):1055–1058. 22246671 10.1158/0008-5472.CAN-11-3439 PMC3341848

[48] Diemer T, Allen JA, Hales KH, Hales DB Reactive oxygen disrupts mitochondria in MA-10 tumor Leydig cells and inhibits steroidogenic acute regulatory (StAR) protein and steroidogenesis. Endocrinology. 2003;144(7):2882–2891. 12810543 10.1210/en.2002-0090

[49] Stocco DM, Wells J, Clark BJ The effects of hydrogen peroxide on steroidogenesis in mouse Leydig tumor cells. Endocrinology. 1993;133(6):2827–2832. 8243310 10.1210/endo.133.6.8243310

[50] Prasad R, Metherell LA, Clark AJ, Storr HL Deficiency of ALADIN impairs redox homeostasis in human adrenal cells and inhibits steroidogenesis. Endocrinology. 2013;154(9):3209–3218. 23825130 10.1210/en.2013-1241 PMC3958737

[51] Zhao Y, Ao H, Chen L, Sottas CM, Ge RS, Li L, Zhang Y Mono-(2-ethylhexyl) phthalate affects the steroidogenesis in rat Leydig cells through provoking ROS perturbation. Toxicol In Vitro. 2012;26(6):950–955. 22525294 10.1016/j.tiv.2012.04.003

