# Supplementary material for: Nicotinamide Nucleotide Transhydrogenase as a Novel Treatment Target in Adrenocortical Carcinoma
Source: Endocrinology. 2018 Apr 20;159(8):2836–49. doi: 10.1210/en.2018-00014 (PMC6093335; doi:10.1210/en.2018-00014)
Supplement: Supplemental Data [file en.2018-00014.sd1.pdf]

Supplementary File

Supplementary Methods

In vitro steroid profiling by liquid chromatography-tandem mass spectrometry (LC-MS/MS)

NCI-H295R cells were incubated in 6-well plates in 1 ml of serum-free DMEM/Ham's F-12 medium (Gibco, Thermo Fisher), supplemented with 1% penicillin-streptomycin and 1% ITS universal cell culture premix. Serum-free media was used, as serum itself contains steroids which may confound results. Media was collected after 48-hour incubation in silanized glass tubes and stored at -20°C. To extract steroids from the media, 20 µl of serum steroid internal standard solution was transferred to each tube, followed by 3 ml Methyl tert-butyl ether (MTBE, Sigma-Aldrich). After vortexing, samples were frozen at -20°C for at least 1 hour. The top layer (liquid phase) was transferred to a 96-well plate using Pasteur pipettes. MTBE was evaporated to dryness at 55°C and samples were reconstituted in 125 µl of 1:1 H_2O/methanol. Steroid metabolites were identified and quantified by LC-MS/MS, with reference to a linear calibration series and appropriate internal standards as described previously (AcquityTM Ultra Performance Liquid Chromatographer, Xevo TQ Mass Spectrometer) (1, 2).

RNA sequencing – NCI-H295R NNT knockdown models

RNA samples from KD siRNA, SCR SiRNA (72 hours post-transfection), KD shRNA and SCR shRNA cells (in triplicates) were prepared using the RNeasy Mini kit (Qiagen). NNT protein knock-down was confirmed independently by Western Blotting. Libraries were generated using the TruSeq Stranded mRNA Library Prep Kit (Illumina).

RNA was quantified using Qubit® RNA BR Assay Kit and if necessary diluted to 25-100 ng in 12.5 µl. This was followed by RNA quality check to establish the RIN (RNA Integrity Number) using the Agilent RNA ScreenTape system. RNA was then processed on the automated Illumina Neoprep

system following the Library prep TruSeq Stranded mRNA Library Prep Kit for NeoPrep. Libraries were normalized to 10 nM by the Neoprep.

Each library quantity was checked again using Qubit DNA HS Assay Kit and 2 μl of each library were pooled together into a single tube. This pool of 16 samples was checked on the Agilent High Sensitivity D1000 ScreenTape to ensure the libraries were the correct size at 300 bp. The pooled sample was diluted to 4 nM. The 4 nM library (containing the 16 pooled libraries) was sequenced on a NextSeq500 using a NextSeq® 500/550 High Output Kit v2 (150 cycles) with a 1% PhiX control spiked in.

Data Quality control was performed with FastQC v0.11.4 (RRID:SCR_014583), revealing no appreciable technical biases. RNA-seq reads were mapped to the human genome (hg19, UCSC annotation) using STAR software v2.4.2a (RRID:SCR_015899) (3) supplied with default parameters. Counts per gene were calculated using the htseq-count tool from the HTSeq v0.6.1p1 package (RRID:SCR_005514) (4) with the following parameters: --format=bam --minaqual=10 --stranded=reverse --mode=union. Differentially expressed genes were identified using the DESeq2 v1.14.1 package (RRID:SCR_015687) (5) from Bioconductor release 3.3. Differentially expressed genes were called at a false discovery rate of 5%. Adjusted p-values for the KD-shRNA vs SCR-shRNA pairwise comparison were re-calculated using fdrtool (6). Pathway Analysis was carried out using GAGE (7) v2.22 package from Bioconductor release 3.3, referencing KEGG pathways (RRID:SCR_012773) and assessing gene sets towards both single directions (up-/down-regulated) and both directions simultaneously (bi-directional). Differentially regulated pathways were called at a p value of <0.01. The accession number for the raw and processed data files for the RNA sequencing analysis reported in this paper is NCBI GEO: GSE106873.

RNA sequencing – mouse adrenals

Aligned BAM files from Meimaridou et al (8) were used to determine counts per gene using custom scripts acting in a HTSeq-count (RRID:SCR_005514) (4) compatible mode with the following parameters: --format=bam --minaqual=10 --stranded=reverse --mode=union. Gene counts were analysed using DESeq2 (v1.14.1) (RRID:SCR_015687) (6) package from Bioconductor release 3.4, and pathway analysis was carried out using GAGE (7) v2.24, referencing KEGG pathways (RRID:SCR_012773) and assessing gene sets towards both single directions (up-/down-regulated) and both directions simultaneously (bi-directional). Differentially regulated pathways were called at a false discovery rate (q-value) of < 0.05.

Whole metabolome analysis

Metabolism was quenched in a) KD siRNA and SCR siRNA cells growing in 6-well plates, 96 hours after cell loading and 72 hours after siRNA transfection and b) KD shRNA and SCR shRNA cells, 96 hours after cell loading. Six biological replicates were used per cell group, each consisting of two wells. 200 μl of media from each well were collected and frozen, followed by media removal and washes with three aliquots of Phosphate-Buffered Saline. Following this, 0.9 ml of a 40/40/20% solution of acetonitrile, methanol and water (Sigma-Aldrich) at a temperature of approximately -40°C was added to each well and plates were frozen at -80°C for 15 min. Cells were scraped off the wells and the suspension was centrifuged at 12,000 rpm (4°C) for 10 min to separate the extraction supernatant from cell pellet. The extraction supernatant was dried applying a vacuum centrifugal evaporator (Thermo Scientific Savant SPD111V speedvac concentrator coupled to a Savant RVT5105 vapour trap). 100 μl of media samples were dried applying the same process. Pooled QC samples were prepared for cell media analysis (by pooling of 100 μl aliquots of all biological samples) and cell extract analysis (by pooling of 200 μl aliquots of all cell extract samples).

Samples were analysed applying ultra-performance liquid chromatography-mass spectrometry (UPLC-MS) using a Thermo Scientific Ultimate3000 UPLC system coupled to an electrospray Q Exactive Focus mass spectrometer. Two assays were applied to increase the number of metabolites detected, a HILIC assay to investigate water-soluble metabolites and a C_{18} reversed phase method to investigate lipid metabolites.

The HILIC method applied a Thermo Scientific Accucore 150 Amide HILIC column (100 x 2.1mm, 2.6 µm) operated at a temperature of 35°C and a flow rate of 500 $\mu L.min^{-1}$. Solvent A was A 10 mM Ammonium Formate in 95% Acetonitrile/5% water + 0.1% formic acid and solvent B was 10 mM Ammonium Formate in 50% Acetonitrile/50% water + 0.1% formic acid. The gradient elution was applied at follows; Start at 99%A for 1 minute, followed by decreases to 85% and 50% at 3 minutes and 6 minutes with a curve of 5 and then a decrease to 5% A at 9 minutes and an increase to 99% A at 10.5 minutes with a curve of 5. The total analysis time was 15 minutes and the injection volume was 2µL. Mass spectral data was collected in positive and negative ion modes separately at a mass resolution of 70,000 (FWHM at m/z 200). Data Dependent Analysis data was acquired for three QC samples to aid metabolite identification. QC samples were analysed 10 times at the start of the run and then after every 6th biological sample with two QC samples analysed after all biological samples had been analysed.

The C_{18} reversed phase method applied a Thermo Scientific Hypersil GOLD C_{18} column (100 x 2.1mm, 1.9 µm) operated at a temperature of 55°C and a flow rate of 400 $\mu L.min^{-1}$. Solvent A was 10 mM Ammonium Formate in 60% Acetonitrile/40% water + 0.1% formic acid and solvent B was 10 mM Ammonium Formate in 90% isopropyl alcohol/10% acetonitrile + 0.1% formic acid. The gradient elution was applied at follows; Start at 80%A for 0.5 minutes, followed by a decrease to 0% A at 8.5 minutes with a curve of 5 and then an increase to 80% A at 11.5 minutes with a curve of 5. The total analysis time was 15 minutes and the injection volume was 2µL. Mass spectral data was collected in positive and negative ion modes separately at a mass resolution of 70,000 (FWHM at m/z 200). QC

samples were analysed 10 times at the start of the run and then after every 6th biological sample with two QC samples analysed after all biological samples had been analysed.

Raw data was converted to the mzML format applying ProteoWizard and then deconvoluted applying the software XCMS (9), operated on an office PC in R using previously described parameters (10). Putative metabolite annotations were provided using the software PUTMEDID_LCMS using a RT window of +/- 2 seconds and a mass error of 5ppm (11).

The data were filtered for quality based on the QC sample data with metabolites with a relative standard deviation >20% or detected in less than 60% of the QC samples being removed (12). Univariate and multivariate data analysis and pathway enrichment analysis were performed in MetaboAnalyst 3.0 (RRID:SCR_015539) (13). This included Principal Components Analysis (PCA), Mann Whitney U tests or Kruskal-Wallis tests to identify metabolites demonstrating a statistically significant change in relative concentrations between two or three biological classes. Fold changes were calculated by division of the mean peak response for one biological class by the mean peak response of the second biological class.

Supplementary Tables and Figures

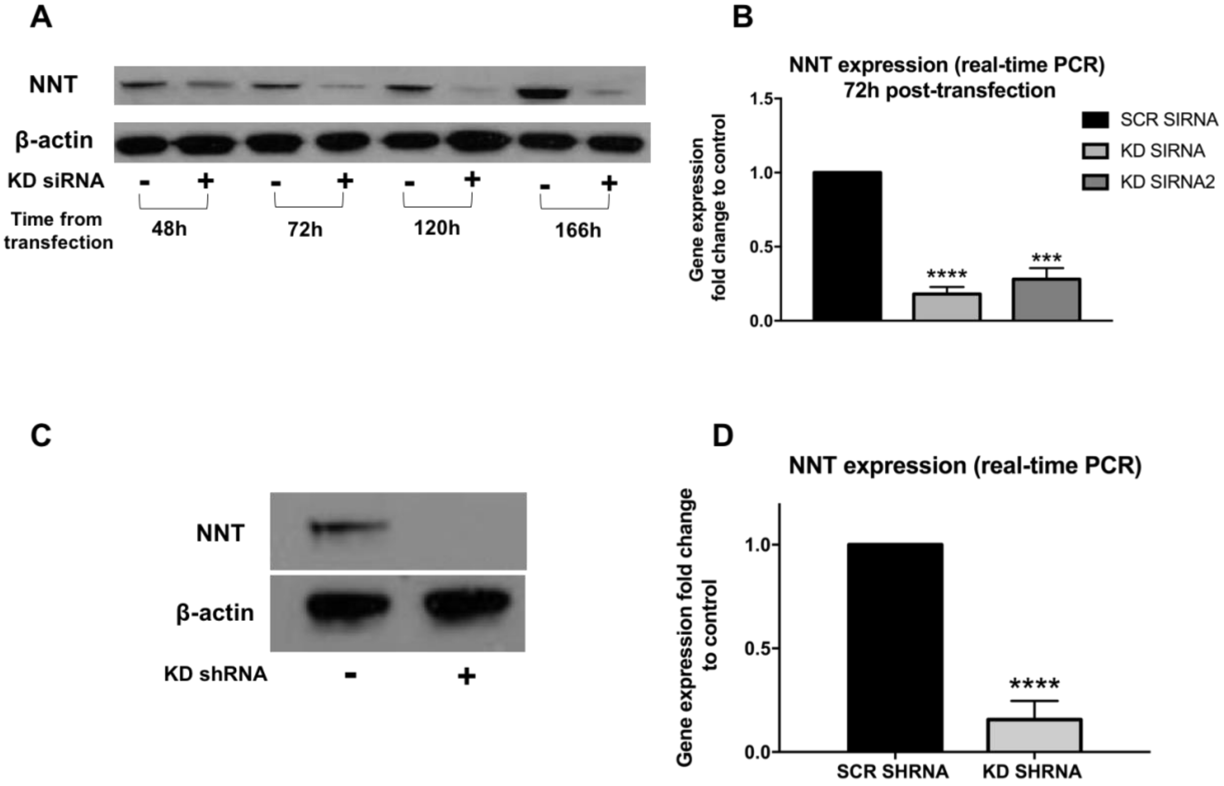

Supplementary Figure 1. A*, Western Blotting confirms efficient NNT protein silencing in NCI-H295R cells transfected with anti-NNT siRNA (KD siRNA), 72-166 hours post transfection.* ***B,*** *Real-time PCR confirms effective NNT silencing 72 hours post siRNA transfection in NCI-H295R cells. Two different siRNAs were tried, targeting different sequences on the NNT transcriptome (KD siRNA and KD siRNA2); KD siRNA2 was used to corroborate the effects observed with KD siRNA transfection on proliferation and apoptosis. Cells transfected with scrambled, non-sense siRNA were used as controls (SCR siRNA). ***p<0.001, ****p<0.0001; n≥10.* ***C,*** *Western Blotting confirms NNT protein silencing in NCI-H295R cells transfected with KD shRNA. Cells transfected with scramble, non-sense shRNA were used as controls (SCR shRNA).* ***D,*** *Real-time PCR confirms effective NNT silencing in NCI-H295R cells transfected with shRNA against NNT (KD shRNA). Cells transfected with scrambled, non-sense shRNA were used as controls (SCR shRNA). ****p<0.0001; n≥7.*

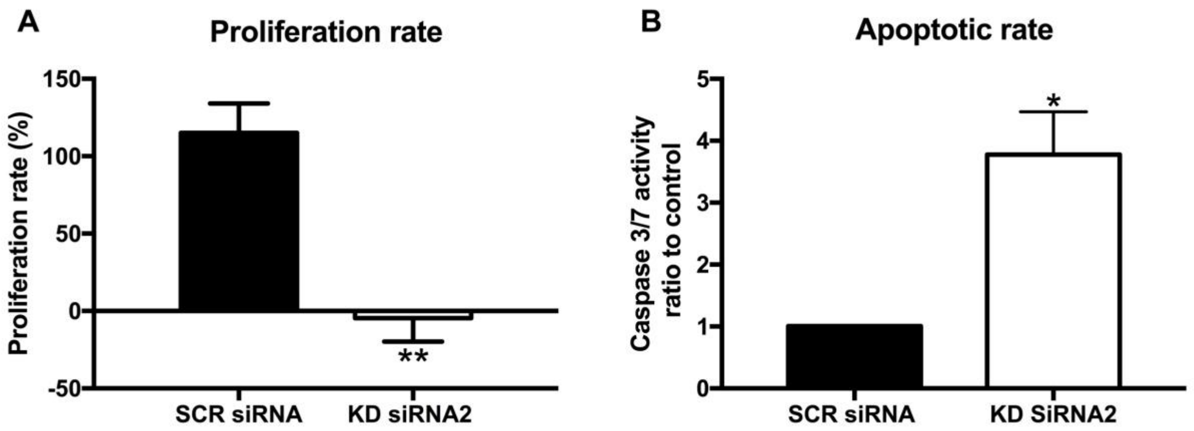

Supplementary Figure 2. Effect of alternative siRNA against NNT (KD siRNA2) on NCI-H295R cell proliferation and apoptosis rates. A, *Proliferation rates observed in siRNA2-transfected NCI-H295R cells, 72-166 h post-transfection. **p<0.01; n=5 independent experiments.* ***B****, Caspase 3/7 activity ratio in KD siRNA2 cells to SCR siRNA-transfected cells, after standardization to cell numbers (120 h post-transfection). *p<0.05; n=5.*

Supplementary Table 1. Genetic STR analysis of H295R cells used in this project, including 22 highly polymorphic areas (loci). *Number of sequence repeats is indicated for each locus.*

Marker	Allele 1	Allele 2
AMEL	X	
D3S1358	15	16
D1S1656	12	
D2S441	11	
D10S1248	14	
D13S317	13	
Penta E	5	
D16S539	11	
D18S51	17	
D2S1338	25	
CSF1PO	10	12
Penta D	8	
TH01	9.3	
vWA	17	18
D21S11	32.2	
D7S820	9	12
D5S818	12	
TPOX	8	
D8S1179	13	
D12S391	19	20
D19S433	13	
FGA	19.2	24
D22S1045	15	

Supplementary Table 2. Steroidogenic enzyme expression in KD siRNA and SCR siRNA cells, assessed by Real-Time PCR. *Results are expressed as median ΔCt (IQR) values. n≥7 independent experiments. StAR: Steroidogenic acute regulatory protein; CYP11A1: Cholesterol side-chain cleaving enzyme; 3βHSD2: 3β-hydroxysteroid dehydrogenase type II; CYP17A1: 17α-hydroxylase- 17/20 lyase; CYP21A2: 21-hydroxylase*

	SCR SiRNA median ΔCt (IQR)	KD SiRNA median ΔCt (IQR)	p value	Median fold change KD siRNA/ SCR siRNA
StAR	4 (2.3-6.8)	2.2 (1.9-3.0)	0.09	
CYP11A1	5.9 (3.3-9.0)	4.8 (3.5-4.9)	0.14	
3βHSD2	8.7 (7.6-10.3)	8 (7.4-8.8)	0.02	2
CYP17A1	2.8 (1.5-5.2)	1.8 (0.7-2.9)	0.03	2.5
CYP21A2	9.3 (4.2-12.9)	6.4 (0.4-9.2)	0.02	11.5

Supplementary table 3. Significant genes that are differentially regulated between KD siRNA and SCR siRNA cells (A) or between KD shRNA and SCR shRNA cells (B) (RNA sequencing). *17 genes highlighted in bold font were significantly altered in both comparisons. Positive values denote up-regulation with NNT knockdown. q<0.05 (False Discovery Rate 5%) was used as the threshold of statistical significance.*

Supplementary Table 3A

**Gene**	**$\log_2$ Fold Change KD siRNA Vs SCR siRNA**	**p value**	**q value**
SLC16A6	1.383218178	$1.95971E^{-15}$	$6.41147E^{-12}$
SLC16A9	1.259199322	$4.2203E^{-20}$	$2.96392E^{-16}$
ARRDC3	1.103592627	$2.28231E^{-15}$	$6.41147E^{-12}$
LOC728175	1.021723337	$2.9811E^{-09}$	$1.39575E^{-06}$
GNRHR	0.895911271	$7.74013E^{-12}$	$9.05983E^{-09}$
CCNE1	0.788621396	$2.81919E^{-09}$	$1.36546E^{-06}$
MAN1A1	0.778872578	$5.13273E^{-07}$	0.000107603
CTSV	0.758410912	$1.66716E^{-08}$	$5.45459E^{-06}$
PPP2R1B	0.715673998	$7.21568E^{-11}$	$5.63063E^{-08}$
C1orf141	0.701674984	$6.26031E^{-05}$	0.00389081
CYP17A1	0.675702613	0.000184861	0.00845784
SPRED2	0.668466019	$2.27935E^{-06}$	0.00037082
MC2R	0.651355465	0.000228438	0.009723137
TAPT1	0.632443173	$3.06564E^{-15}$	$7.17665E^{-12}$
ARSG	0.62590135	0.000294315	0.011328988
GNAI1	0.618791402	$9.6579E^{-07}$	0.00018841
ZNF678	0.614386714	$4.41421E^{-06}$	0.000607863
CA2	0.606754247	0.000550892	0.017157034
MTF2	0.599485848	$1.75993E^{-09}$	$9.50767E^{-07}$
ARHGAP12	0.599306312	$1.02643E^{-07}$	$2.6736E^{-05}$
ZNF763	0.598863323	0.000292769	0.011328988

DPP4	0.593070146	0.000503607	0.016298764
IL23R	0.59226251	0.000539068	0.016964399
ITGA1	0.585001105	0.00086819	0.022658038
ITGB8	0.578320032	0.00090321	0.023192846
MSMO1	0.577036521	8.33654E^{-05}	0.004878958
MET	0.571708587	5.63755E^{-05}	0.003534264
MGAT4A	0.564163158	0.000436759	0.014743703
MACC1	0.560597901	0.002080498	0.039650849
CYP21A2	0.560441003	0.001429487	0.031226397
RGL1	0.560328372	1.58752E^{-06}	0.000282257
NRBF2	0.560125717	1.6267E^{-05}	0.001476118
SULT2A1	0.558771598	0.000370974	0.013225124
IKBKAP	0.555933991	1.5175E^{-05}	0.001420989
NEU1	0.555882407	0.000163965	0.007651324
DUSP4	0.547314248	0.001670006	0.034990186
CITED2	0.54701362	0.000420118	0.014414836
PLK2	0.54415396	0.002344394	0.04270993
PTPMT1	0.544114794	1.60059E^{-05}	0.001469406
E2F2	0.543856464	2.46997E^{-10}	1.73466E^{-07}
PLSCR4	0.542943203	4.89159E^{-06}	0.000662283
MSRB3	0.536027719	4.11192E^{-09}	1.8631E^{-06}
ARL4D	0.535615205	0.000394721	0.013860632
PLLP	0.533817534	0.002906259	0.048770984
LBR	0.530390577	2.82822E^{-07}	6.30559E-05
PDE7B	0.525790885	0.00013728	0.006862053
GPR137C	0.525605479	7.26486E^{-05}	0.004360776
DDX18	0.52061528	3.57513E^{-10}	2.28256E^{-07}
SLC38A2	0.516908523	3.55185E^{-06}	0.000509075
PDCD6	0.516729505	5.67892E^{-08}	1.59532E^{-05}
SLC2A3	0.512212935	0.000184148	0.008452757
DCP2	0.51065777	5.22544E^{-06}	0.000691004
UGCG	0.510430232	0.001532866	0.032921467

FAM178A	0.508057229	0.00060397	0.018166948
SLC38A9	0.506291005	0.000378962	0.013436491
DLC1	0.505975183	2.57228E^{-05}	0.002072238
DRP2	0.502053068	0.002229753	0.041224498
ODC1	0.502018384	4.65752E^{-05}	0.003101868
WEE1	0.496812958	1.16891E^{-05}	0.001190671
C2CD2	0.494506882	0.000234175	0.00981857
MAP2K4	0.49410377	0.000415335	0.014414836
TYW3	0.490052348	2.03923E^{-06}	0.000336977
DNMT3B	0.488671044	0.001850909	0.037030036
ETV1	0.487963762	1.66174E^{-05}	0.001486675
COL15A1	0.483502139	0.000357364	0.012903696
MICU3	0.482772281	0.000120267	0.006303252
TCERG1L	0.482710076	0.002686552	0.046341781
SIX2	0.481735626	0.000614755	0.018411192
AQP11	0.479356148	0.000527492	0.016838998
RBPJ	0.478536856	5.8081E^{-06}	0.000728399
SLC17A5	0.475561228	0.000181465	0.008356893
RFK	0.474934962	9.86232E^{-06}	0.001056074
ZCCHC3	0.474168664	2.34513E^{-06}	0.00037082
DICER1	0.472110682	0.000906736	0.023240893
GOLGA4	0.470671062	8.61391E^{-06}	0.000967928
SBNO1	0.467053151	3.3112E^{-06}	0.000483589
HIC2	0.465264904	0.001552439	0.03306235
ARHGEF6	0.46436213	4.13069E^{-05}	0.002890941
LHFPL2	0.464235212	1.33655E^{-05}	0.001300404
AMDHD1	0.461513434	0.000339667	0.012621599
GPSM2	0.461343302	3.30934E^{-07}	7.26297E^{-05}
LIPT2	0.457860911	0.001350959	0.030052382
BOD1	0.457396612	0.000194451	0.008782201
CREBL2	0.456851397	1.66985E^{-08}	5.45459E^{-06}
ZSWIM4	0.454725159	0.000208264	0.009113014

VCAM1	0.452412037	0.000542536	0.016972088
ZNF441	0.452204029	0.000996952	0.024916693
PAX2	0.451189863	0.002760969	0.046981814
ANGPT1	0.450124438	9.37121E-05	0.005350735
SPRED1	0.449934783	0.000242903	0.010005331
PCSK6	0.44752782	6.47817E^{-05}	0.003973466
KIAA1147	0.447169844	5.4371E^{-06}	0.000701239
SLC45A4	0.443566994	0.000223012	0.009579306
**DHFRL1**	0.442526775	1.29954E^{-05}	0.001288913
FRY	0.441461555	0.001999336	0.03862816
MED21	0.441074062	0.000160305	0.007530589
PLK4	0.438164834	2.407E^{-05}	0.002012427
AIF1L	0.437554259	0.000448505	0.01489291
BMP2K	0.43650997	0.000885198	0.022855684
TMEM65	0.436500989	5.56813E^{-05}	0.003507173
ING2	0.435537424	2.214E^{-07}	5.25109E^{-05}
WSB1	0.434970323	1.41851E^{-06}	0.000262162
FAM19A4	0.433491627	0.00243137	0.04378337
RMI2	0.433230738	1.84801E^{-05}	0.00161225
TOMM20	0.432611531	0.0001255	0.006462374
GPR137B	0.432140946	0.000110674	0.005910731
YRDC	0.431873593	4.42872E^{-05}	0.002994804
NRAS	0.43159367	1.00964E^{-05}	0.001066274
STIM2	0.428215051	0.000213364	0.009267948
**G3BP2**	0.427849428	8.62523E^{-11}	6.37631E^{-08}
PPP1CC	0.427838671	1.57073E^{-05}	0.001451481
TESK2	0.426926903	0.000246783	0.010105874
PLEKHA5	0.426508645	1.64784E^{-05}	0.001483691
CDK8	0.424442513	3.88579E^{-05}	0.002808317
NTAN1	0.42443504	7.20739E^{-06}	0.000857924
EEA1	0.423991582	6.35793E^{-05}	0.003934072
ERCC5	0.421813797	0.001577531	0.033471297

KATNBL1	0.420601594	$3.70958E^{-05}$	0.00271379
**CIDEB**	0.419674529	0.000994688	0.024904443
PTPN3	0.419638217	$3.58458E^{-05}$	0.002636071
ZNF143	0.418412231	0.000119697	0.006296857
**H1F0**	0.417627495	0.000163209	0.007641445
FAAP24	0.416550212	0.000201321	0.008934482
GJA1	0.416324894	0.001150017	0.027106682
GLA	0.414672661	$9.34974E^{-05}$	0.005350735
POLQ	0.413759191	$6.75548E^{-05}$	0.004107683
GBA	0.412475082	0.000445065	0.014865951
ZDHHC17	0.411924596	$1.67464E^{-05}$	0.001488732
STX12	0.411732272	$4.00986E^{-06}$	0.000568915
AKAP7	0.411656295	0.000270877	0.010745219
PVRL3	0.411093224	0.000217761	0.009411295
PREPL	0.410782167	0.000353906	0.012844877
SP4	0.408536925	0.001347899	0.03005172
TDG	0.407263585	$3.03286E^{-05}$	0.002353564
PLAGL1	0.406657762	$1.34244E^{-05}$	0.001300404
LAMTOR3	0.406238639	$7.89489E^{-06}$	0.000901558
DIXDC1	0.405252406	$3.39652E^{-05}$	0.002553861
PRRC2C	0.404961973	0.000204455	0.008980917
GABPA	0.402784031	$2.0123E^{-05}$	0.001734038
HMGN5	0.402665522	0.001806644	0.036830372
BTRC	0.402648359	$1.31222E^{-05}$	0.001288913
HSPD1	0.401802319	$1.79438E^{-06}$	0.000315048
MED4	0.401161503	$1.17082E^{-05}$	0.001190671
MAMSTR	0.40080178	0.001454078	0.031519771
SKIL	0.400726805	0.000240187	0.009922569
ANKRD44	0.399826305	0.000155628	0.007486149
CASD1	0.3995631	0.00010217	0.005605781
DET1	0.399308587	0.000125604	0.006462374
SC5D	0.399183706	0.001310365	0.029440137

MAGI2	0.39878402	0.000105947	0.005749612
C1D	0.398515503	0.0004467	0.014868125
ARL6IP5	0.398050569	0.001146779	0.027106682
ARFGEF2	0.39554849	$7.6691E^{-05}$	0.004545156
SMARCD2	0.395028724	0.000138731	0.006885546
ZSCAN21	0.393515948	0.000306085	0.011651119
ELMOD2	0.39303963	$5.44949E^{-06}$	0.000701239
EYA3	0.392313927	0.000334547	0.01249748
GORAB	0.392205154	0.00091105	0.023308951
SRGAP2B	0.389257319	0.000667814	0.01933575
FAM133B	0.388690627	0.002003068	0.03864711
SKIDA1	0.38838865	0.001977298	0.038360674
FOXN3	0.387657969	0.000229627	0.009744224
SRGAP2	0.38735527	0.000289103	0.011311264
ZNF625	0.386613139	0.002685761	0.046341781
ENAH	0.386340533	0.000509964	0.016428787
RCOR1	0.386281734	2.51755E-05	0.002072238
C2orf69	0.384767589	0.001204822	0.027788072
**NEK7**	0.383722681	0.002190509	0.040952756
RAD50	0.38365852	0.000213784	0.009267948
RALGAPA2	0.381402334	0.001196083	0.027631882
COL4A3BP	0.379787699	0.000535738	0.016964399
SYPL1	0.379686594	$2.63857E^{-05}$	0.002105756
MGME1	0.378864919	0.000440507	0.014773295
STMN1	0.377781548	0.000224701	0.00959315
RRAGC	0.377428393	0.001127614	0.026941873
PLXDC2	0.376303579	0.001529363	0.032896533
RAB7A	0.37586694	0.000758203	0.020880059
ISOC1	0.375663425	0.001689069	0.035199802
HMGB2	0.375259487	0.000706954	0.020100954
MOB4	0.375251374	0.002444575	0.043852496
EPB41L4B	0.375220821	0.000343981	0.012648053

DISP1	0.373164532	0.001239061	0.02839127
ZNF641	0.372862881	0.000859583	0.022567673
SCML2	0.371806486	0.002681092	0.046341781
ANKRA2	0.371257521	0.000416216	0.014414836
ATG2B	0.369074255	0.001987288	0.038501309
TIMM22	0.36869627	$8.11502E^{-05}$	0.004769187
MBTPS1	0.368666762	$1.45331E^{-05}$	0.001388648
ATF1	0.367788456	0.000367836	0.013146615
APBB2	0.367636769	0.000302826	0.011558414
ESYT2	0.367609065	$1.12842E^{-06}$	0.000217121
COX10	0.366474873	0.000231198	0.009781362
DDX3X	0.365974588	$1.82506E^{-05}$	0.001602172
ALDH3A2	0.365755806	0.002577868	0.045148052
CEP135	0.364292587	0.001016934	0.025325976
ZNF738	0.36326512	0.001139002	0.027026409
ALG9	0.363252169	$3.49722E^{-05}$	0.002612872
KLF3	0.362116765	0.000325375	0.012252583
C1orf21	0.361725135	0.001190521	0.027611899
ALG14	0.361172886	0.001900089	0.037484069
UEVLD	0.360988169	0.001855956	0.037030036
PPP3CB	0.36079551	$1.54514E^{-05}$	0.00143729
DDX10	0.36025669	0.002284524	0.041781801
CDC25A	0.358062394	0.001307101	0.029422339
CTNS	0.357501567	0.000535243	0.016964399
JAZF1	0.357354903	0.002158071	0.04061926
MBNL3	0.356986384	0.000693578	0.019841126
PNRC2	0.356804624	0.001116376	0.026879785
RTN4	0.35633992	$2.17691E^{-05}$	0.001864445
CTBP2	0.35396556	0.000147968	0.007209639
PPFIBP1	0.353462181	0.001358213	0.030052382
SEPT11	0.351952745	0.002657573	0.046141256
PIK3C2A	0.351488017	0.000717442	0.020235321

THAP1	0.351469324	0.001104581	0.026749899
HMGCR	0.351171023	0.002974969	0.049627577
RNF6	0.349822363	0.000651893	0.019128984
TRMT10A	0.349734868	0.000920259	0.023501741
SPOPL	0.34960625	0.000841028	0.022246853
TADA2B	0.347219856	0.000558111	0.017229075
DCP1A	0.34689833	0.000380416	0.013436491
PDS5B	0.346096563	0.002179739	0.040876648
SLC44A2	0.345427114	0.000646636	0.019121368
IRS1	0.343972684	0.001256726	0.028563054
PUM2	0.342604439	0.001188783	0.027611899
THOC2	0.341528224	0.000708638	0.020108128
RIF1	0.339507294	0.000578274	0.017773395
PRPF38A	0.339224598	$5.26395E^{-06}$	0.000691004
MTRF1L	0.338211507	0.002356839	0.042770232
VPS54	0.336510146	0.000123389	0.006408328
MAPK6	0.336395793	0.000670405	0.01933575
SSH2	0.336009178	0.002107529	0.039969929
PHACTR2	0.335704523	0.002703122	0.046394763
COX17	0.335499503	0.000675884	0.0194197
ZNHIT6	0.335282833	0.000955332	0.024045625
NUP50	0.334955768	0.001191287	0.027611899
TRPM7	0.334780224	0.000541083	0.016964399
STAG2	0.334718384	1.30478E-05	0.001288913
RPE	0.334605394	0.001638517	0.034453012
MAGI1	0.334246557	0.00188752	0.03738556
BPTF	0.333898755	0.001324294	0.029666726
CRY1	0.333596293	0.001816707	0.036920417
**C9orf40**	0.332332754	0.000234069	0.00981857
HMGN2	0.3321557	0.002199918	0.040952756
RCC2	0.331879266	3.57643E-05	0.002636071
GOPC	0.329605391	0.001671537	0.034990186

TRDMT1	0.328861946	0.002196466	0.040952756
RYBP	0.328018844	0.001450447	0.031519771
ZNF12	0.327007104	$7.65808E^{-05}$	0.004545156
ZNF627	0.326584405	0.001045671	0.025914426
H2AFY2	0.326467185	0.00283827	0.04803174
GOLT1B	0.325426114	0.000431697	0.014681872
SMAP1	0.325255591	0.000423657	0.014478539
AMD1	0.32473083	0.001553549	0.03306235
THUMPD1	0.324670826	0.001141012	0.027026409
HSPA4L	0.324414833	0.002537165	0.044882888
CHEK1	0.324001614	0.00011252	0.005986599
CCDC93	0.323912839	0.000420766	0.014414836
TARS	0.322949467	0.001101285	0.026716143
RAD21	0.321665368	0.00109034	0.026496403
TSC1	0.321648287	0.000720341	0.020262828
SFMBT1	0.320524563	0.000445576	0.014865951
SEC62	0.319961853	0.000337734	0.01258306
RP9	0.319282209	0.001563566	0.033225177
PEG10	0.319210855	0.000710211	0.020110932
EXOC6	0.316621398	0.000200111	0.008934482
KIAA1033	0.315453195	0.000752132	0.020796165
ARMC8	0.314234535	0.000253325	0.010313635
PTPN12	0.314170623	0.000580149	0.017792076
TVP23B	0.312708104	0.000628744	0.018750175
PTPN2	0.312209824	0.000123641	0.006408328
NUMB	0.311911357	0.002282628	0.041781801
CDH2	0.31185075	0.002703485	0.046394763
FAM122A	0.311641026	0.002461317	0.044040328
POLR3F	0.311273787	0.002011294	0.038702111
DDX17	0.310614711	0.001174926	0.027467601
FBXL7	0.309267515	0.000211465	0.009224323
DSCR3	0.308384026	0.000237409	0.009861816

STX6	0.308360284	0.000499729	0.0162106
FNTA	0.307814088	0.000518436	0.016658293
SLC31A1	0.307424643	2.58181E^{-05}	0.002072238
LZIC	0.306907626	0.002377631	0.043036349
INSR	0.306531283	0.001058994	0.026141706
SON	0.306284559	0.000871091	0.022658038
FAR1	0.30248949	0.001837074	0.037017786
TMEM168	0.302476342	0.001783243	0.036512285
HMGN1	0.302185044	2.57813E^{-05}	0.002072238
SLC9A6	0.301992132	0.001083134	0.026412674
LARP7	0.301509875	0.00027028	0.010745219
ZHX1	0.300855887	0.000258485	0.010403085
TLK1	0.300827492	0.001262319	0.028643837
KLHL42	0.299468829	0.000158843	0.007521722
NOP56	0.298995746	0.000321622	0.012176558
EIF3A	0.298724279	0.000106429	0.005749612
PTK2	0.298496449	0.000100535	0.005537698
FEZ2	0.297984984	0.001840929	0.037017786
PATL1	0.297161206	0.00075768	0.020880059
OGT	0.296896763	0.000333009	0.012473194
UBE2D3	0.296781724	0.000495105	0.016097789
PANK3	0.295944426	0.000792952	0.021377733
PRKAR2B	0.295931498	0.000847233	0.022368858
TBC1D15	0.295395134	0.000425652	0.014511431
DMTF1	0.2938517	0.000598683	0.018084104
STK4	0.293842951	0.002057002	0.039363289
MRPL48	0.293591034	0.002953778	0.049406943
USP32	0.292949692	0.002448418	0.043865401
CD59	0.291844338	0.001964856	0.038199261
MTAP	0.290632559	0.000236342	0.009861816
DCAF10	0.287975987	0.002211534	0.041005242
GSE1	0.287864863	0.000589496	0.018000141

PTPN1	0.287810036	9.92466E-06	0.001056074
NUS1	0.287306166	0.00107363	0.026272143
RNF139	0.286322489	0.000264917	0.010631488
AKIRIN1	0.285247035	0.001150191	0.027106682
PPIA	0.284955678	0.000435424	0.014737258
RPIA	0.283708539	0.002574449	0.045144454
IPO7	0.283270632	0.002348966	0.042737787
CD3EAP	0.282782185	0.002756528	0.046981814
PPP6R3	0.282021692	$9.03905E^{-05}$	0.005224797
POLR3A	0.282012288	0.001341146	0.029996395
RWDD4	0.281858937	0.002605199	0.045456685
**CDCA4**	0.281398791	0.00247392	0.044097317
CHUK	0.280732377	$9.90725E^{-05}$	0.00552211
ALG5	0.28067173	0.001231594	0.028312553
SDCBP	0.27988087	0.000958675	0.024045625
MAPK1IP1L	0.278660767	0.000142858	0.007065431
LAPTM4A	0.278390698	0.000341806	0.012634244
SMARCD1	0.278090473	0.000302219	0.011558414
MORF4L1	0.277700081	$4.15755E^{-05}$	0.002890941
NET1	0.277688274	0.002036267	0.039072959
SAP130	0.276860625	$6.0866E^{-06}$	0.00075657
SPPL2A	0.276832305	0.00041561	0.014414836
DAZAP2	0.275639531	0.000738692	0.020586655
BTF3L4	0.275348683	0.002299223	0.04199596
KIAA0513	0.275061531	0.00285932	0.048329736
RSRC1	0.274491319	0.0008844	0.022855684
ADIPOR1	0.274198767	0.000655865	0.019192241
RUFY1	0.274160054	0.002336337	0.042618437
RABL3	0.273643297	0.001852892	0.037030036
DYRK1A	0.273250137	0.000663783	0.019303292
DNAJC9	0.272546626	$4.31139E^{-05}$	0.002954038
NCKAP1	0.271690051	0.000868033	0.022658038

VMP1	0.271170382	0.002504635	0.044434417
LINC01578	0.270556436	0.002688919	0.046341781
PHAX	0.270146015	0.001454137	0.031519771
TNPO3	0.269143137	0.000748183	0.020727779
ITGB1	0.269069865	0.002205309	0.040973232
FAM103A1	0.267733405	0.001435433	0.031307595
AHI1	0.267232326	0.002439216	0.043852496
PPP4R3A	0.267143052	0.000196962	0.008867068
ARF6	0.266389047	0.00201143	0.038702111
ABHD10	0.264978388	0.002077722	0.039650849
RABGAP1	0.264895918	0.001777243	0.036512285
KPNA1	0.26458193	0.000160234	0.007530589
NUP160	0.264302759	0.002801305	0.047520689
TOPBP1	0.264281887	0.001618248	0.034180318
USP46	0.263980039	0.002882272	0.048536712
TTL	0.263551615	0.002783526	0.047276185
WAC	0.263447042	0.002882589	0.048536712
RSL24D1	0.262881874	0.002585017	0.045170447
SEPHS1	0.258387825	0.000440696	0.014773295
TXLNG	0.256796441	0.002263242	0.041656772
GPBP1	0.256384375	0.001108907	0.026808457
SPG20	0.256091557	0.000267948	0.010692056
CASK	0.255870751	0.00193501	0.037906768
PFN1	0.25529201	0.000126895	0.00649253
CFL2	0.254064536	0.000933382	0.023707554
SRSF11	0.253974623	0.0021243	0.04021282
RPL15	0.253039591	0.001173256	0.027467601
SMUG1	0.252699312	0.001509533	0.032519795
LARS	0.251807986	0.001072252	0.026272143
SMC1A	0.251579896	0.002201291	0.040952756
TCERG1	0.251313253	0.001959763	0.038178679
NPTN	0.250618556	0.001714129	0.035506783

TOMM6	0.249729858	0.000554329	0.017187875
DHX15	0.243819556	0.001821514	0.036920417
SEL1L	0.241778894	0.002885388	0.048536712
HOXA10	0.240812123	0.001363604	0.030115065
SMC3	0.238169996	0.002549814	0.044949964
ARCN1	0.234152434	0.001220339	0.028099795
PKM	0.233646356	0.000100368	0.005537698
HIF1AN	0.232009054	0.002274102	0.041754294
DHX9	0.231546852	0.000271576	0.010745219
SP3	0.230691455	0.001930639	0.037906768
IREB2	0.230625736	0.001874228	0.037216549
RBBP6	0.230551606	0.000955763	0.024045625
POLR2K	0.230372864	0.001869455	0.037216549
ZNF655	0.229860798	0.001491069	0.03222085
SEC31A	0.229382654	0.00140326	0.030749133
CUTA	0.229272721	0.000810129	0.021715774
SERBP1	0.226265573	0.000805097	0.021634524
SET	0.224759899	0.002551022	0.044949964
ATXN7L3B	0.224274791	0.002363149	0.042829413
TACC1	0.223618705	0.001661834	0.034891069
SARS	0.219920223	0.001824204	0.036920417
SRSF4	0.219554521	0.001872582	0.037216549
CWC15	0.219531355	0.001252857	0.028563054
ZDHHC7	0.217271571	0.001122824	0.026941873
PARD3	0.21682903	0.000234088	0.00981857
CNOT2	0.206050296	0.002876694	0.048536712
CAPZA2	0.201801085	0.002679607	0.046341781
**TSTD2**	0.201039339	0.001358626	0.030052382
DLD	-0.18955922	0.001312084	0.029440137
EIF4H	-0.197944909	0.0012384	0.02839127
CMTR1	-0.231337833	0.00221287	0.041005242
ATOX1	-0.232397299	0.002521409	0.044660412

C6orf120	-0.234397669	0.001088531	0.026496403
VIM	-0.237518011	0.000159045	0.007521722
RSU1	-0.237935816	0.000324253	0.012243158
CSNK1A1	-0.244201077	0.001678568	0.035032945
VOPP1	-0.251570066	0.0016966	0.035304361
TMED4	-0.25195419	0.002108623	0.039969929
EIF2B4	-0.255725896	0.001824066	0.036920417
CD81	-0.257026802	0.00238854	0.043178157
ACTR1A	-0.258409012	0.001127853	0.026941873
DTD1	-0.273831895	0.0007116	0.020110932
GALNT13	-0.27530258	0.001942094	0.037931801
NAPG	-0.275519677	0.001346003	0.03005172
YBX3	-0.276012382	0.002093287	0.039829755
ITPK1	-0.276848888	0.001538921	0.032950733
CMSS1	-0.278271818	0.002812339	0.04765031
BLVRA	-0.279590992	0.000633113	0.018840475
PROSER1	-0.281296004	0.001068532	0.026262955
HYOU1	-0.281714706	9.34739E-06	0.001017778
DCTN2	-0.284412983	0.001273454	0.028803446
NLN	-0.288287981	0.001411569	0.030883023
PLXNB2	-0.288305458	0.001137613	0.027026409
IDE	-0.288746288	0.000593211	0.018035147
LIMA1	-0.294062993	0.001245209	0.028439355
ETV6	-0.295473593	0.000418155	0.014414836
RUVBL2	-0.295504777	0.002965763	0.049532826
TCF7L2	-0.295882155	0.00055625	0.017209456
C18orf25	-0.297227842	0.00192101	0.037827605
SLC35B4	-0.29755565	0.002585579	0.045170447
ASPH	-0.299610908	0.000649186	0.019121485
PDZRN3	-0.301259536	0.000484477	0.015862379
G6PD	-0.30152022	0.002505486	0.044434417
HDAC5	-0.313999158	0.000100146	0.005537698

ZXDB	-0.314671352	0.00194439	0.037931801
FBXO42	-0.315501204	0.000156282	0.007491945
CDH12	-0.316903871	0.000201729	0.008934482
POLDIP2	-0.317409657	0.000590931	0.018004799
DECR1	-0.318453237	0.001014509	0.025310471
SRM	-0.31878575	0.00012756	0.00649253
CASP7	-0.320077144	0.000584768	0.01789468
MANEAL	-0.322196333	0.000349847	0.01281619
TRAPPC4	-0.322893354	0.001505325	0.032478951
COMMD7	-0.324364295	0.001538061	0.032950733
GINM1	-0.325094944	0.000825677	0.021933556
GTF3C2	-0.325570881	0.001675622	0.035023482
BCL2L1	-0.326087164	0.002762849	0.046981814
ABR	-0.326576523	0.000784719	0.021316165
ADCK4	-0.326780855	0.002709485	0.046394763
UBE2Q2	-0.329210343	0.002903786	0.048770984
PCTP	-0.333633317	0.000638535	0.018921653
ETNK2	-0.335000092	0.002544359	0.044949964
ZWINT	-0.335763678	0.002143696	0.040426363
TMED2	-0.336707812	$9.47973E^{-06}$	0.001024249
AXIN2	-0.338658517	0.000956931	0.024045625
TTC12	-0.338889604	0.002954708	0.049406943
SLC25A3	-0.339353223	0.000110039	0.005910731
FCHO2	-0.339604515	0.000128677	0.006524883
ZBTB8A	-0.340052411	0.00052061	0.016658293
TIRAP	-0.341254482	0.002231041	0.041224498
LYRM7	-0.342076969	0.002076333	0.039650849
ABHD4	-0.342996216	$7.32855E^{-05}$	0.004380293
AGPAT2	-0.346788136	0.00216023	0.04061926
LRPAP1	-0.347998354	5.18508E-06	0.000691004
UNKL	-0.349246435	1.96013E-06	0.000331711
IQCH	-0.353745978	0.002692886	0.046353278

TMEM245	-0.35790812	0.000138494	0.006885546
ATP13A2	-0.359071693	0.000778206	0.021265931
SOD2	-0.359358127	3.14915E-05	0.002430384
KIF1C	-0.359629896	0.000350379	0.01281619
ZNF367	-0.359808737	0.000190532	0.008660826
FAM46C	-0.36095502	0.001628866	0.034352934
PAQR5	-0.361298866	0.000705149	0.020090305
CHPF	-0.361398189	0.000536652	0.016964399
FLII	-0.361814698	1.46516E^{-06}	0.000263841
GANAB	-0.361844254	3.06361E^{-06}	0.000452963
PRKCSH	-0.362106771	2.76988E^{-05}	0.00218133
RALGDS	-0.362384624	0.000864663	0.022658038
ZNFX1	-0.363369962	0.00026758	0.010692056
EMC2	-0.364571461	4.43485E^{-05}	0.002994804
C14orf159	-0.365341675	0.000255148	0.010324939
KIAA1804	-0.365847835	0.000367516	0.013146615
DYRK2	-0.366212609	0.000684389	0.01961821
MOSPD1	-0.36638983	0.000169006	0.007860451
STK36	-0.366523579	0.000204606	0.008980917
NSUN4	-0.368695918	9.64572E^{-05}	0.005445493
C4orf3	-0.36875413	2.56589E^{-05}	0.002072238
UBL7	-0.369183736	0.000746402	0.020727779
HINT3	-0.369265759	5.50413E^{-05}	0.003498239
SEPW1	-0.37098234	0.002467269	0.044071809
ZNF436	-0.372325474	0.00187593	0.037216549
NTM	-0.372478379	0.001934837	0.037906768
IKBKE	-0.372689604	0.001716439	0.035506783
UBA7	-0.37350552	0.001022591	0.0254218
CIPC	-0.373567979	0.000596132	0.018084104
MPI	-0.373727063	0.000154652	0.007464731
MOCS1	-0.374219449	0.000467352	0.015409452
LIX1L	-0.374295856	0.00219106	0.040952756

DPCD	-0.376065952	0.000404866	0.01414613
EIF3I	-0.376198078	$2.75728E^{-05}$	0.00218133
TBL1X	-0.376637546	0.000294434	0.011328988
SIMC1	-0.37724029	0.000892207	0.022994381
RALY	-0.377792645	0.000394248	0.013860632
C1orf198	-0.377807865	$4.13964E^{-05}$	0.002890941
FGF13	-0.378542053	$4.23563E^{-05}$	0.002916359
IL17RC	-0.379254808	$4.65965E^{-05}$	0.003101868
TERF2IP	-0.379570535	0.000553878	0.017187875
BRE	-0.379775815	$5.35842E^{-05}$	0.003421109
ESYT1	-0.380574637	0.000157055	0.007503361
BRINP2	-0.380581557	0.000351919	0.012839087
PMP22	-0.38067865	0.000539599	0.016964399
FLYWCH2	-0.380711517	0.001852947	0.037030036
VPS9D1-AS1	-0.381135646	0.00093059	0.023705862
PFKP	-0.382127849	0.002493961	0.044398194
TBC1D19	-0.382239745	0.000788663	0.021318877
ENGASE	-0.383298422	0.001439565	0.031349036
CRELD2	-0.384977508	0.001892434	0.03738556
GLB1L2	-0.385156758	0.000738157	0.020586655
IGSF8	-0.387147313	0.000852839	0.022474638
SS18	-0.387235712	$9.15583E^{-06}$	0.00101262
SS18L2	-0.389148718	0.000197627	0.008868595
ARSB	-0.389243781	0.00192289	0.037827605
BCL2L2	-0.390273391	0.000134406	0.00674239
DNAJB2	-0.391799529	0.000353522	0.012844877
ANGEL2	-0.393081934	0.000145532	0.007147339
STAT5A	-0.393197431	0.002642584	0.045994708
EML1	-0.39346381	0.000634771	0.018849892
AGAP2-AS1	-0.39360994	0.001707615	0.035480996
CASP9	-0.393612609	0.001379371	0.030367788
CKAP4	-0.394619317	0.000473083	0.015525523

SDF2L1	-0.39524631	0.000546006	0.017042663
CLDND1	-0.397217482	0.000341719	0.012634244
CD151	-0.398468043	0.000238015	0.009861816
ETFB	-0.398720489	0.000219381	0.009452208
PPP1R26	-0.39995587	0.000252011	0.010289979
TMEM167A	-0.400093473	$4.9037E^{-06}$	0.000662283
SLC25A23	-0.400927625	0.001966258	0.038199261
C1QTNF1	-0.401168716	0.002052709	0.039334725
TLE3	-0.401398313	0.002135091	0.040362706
STIM1	-0.401941438	0.000669589	0.01933575
RNF43	-0.404960099	0.001400411	0.030734634
ZNF385A	-0.405631311	0.000823265	0.021933556
APLP1	-0.408276246	0.000665839	0.019323101
ARRB2	-0.408589642	0.001163031	0.027363368
PPP1R18	-0.409142825	$7.71926E^{-06}$	0.000888728
MMP24-AS1	-0.410198376	$5.244E^{-05}$	0.003363343
ZNF652	-0.411188273	0.000789251	0.021318877
NT5DC1	-0.411634516	$2.36972E^{-06}$	0.00037082
SPATA20	-0.414131633	$4.85508E^{-05}$	0.003186654
RFPL1S	-0.415076106	0.000181169	0.008356893
LGMN	-0.415303003	$2.5771E^{-05}$	0.002072238
NT5C3A	-0.415499535	0.001114395	0.026879785
INPP5J	-0.415818804	0.001265816	0.028676861
OGFRL1	-0.416085215	0.001782811	0.036512285
CTNNBIP1	-0.417560868	0.000144264	0.007109914
NEFL	-0.417743173	$8.94267E^{-05}$	0.005190442
FAM43A	-0.418145855	0.001779811	0.036512285
THSD4	-0.421651594	0.000568783	0.017520013
TMSB15B	-0.422603964	0.002095557	0.039829755
CNKSR2	-0.423317565	0.001793541	0.036616382
GPC4	-0.425104615	0.000437714	0.014743703
STMN3	-0.425165643	0.000202276	0.008934482

MARCH2	-0.425459881	0.002265825	0.041656772
SLC36A1	-0.426204089	0.001083085	0.026412674
C10orf82	-0.426244241	0.000174103	0.00807079
IL13RA1	-0.426594979	0.000598599	0.018084104
CCDC113	-0.426878644	0.001063155	0.02619837
NEFM	-0.426937624	0.000786115	0.021316165
DOHH	-0.427682394	0.002168339	0.040717227
MR1	-0.427919433	9.88282E-05	0.00552211
NUMBL	-0.42835969	0.000355213	0.012859068
SYNJ2	-0.429143866	0.000397474	0.013922498
TTC7A	-0.429594406	1.4714E^{-05}	0.001396434
MFSD12	-0.429838904	0.002236442	0.041224498
ULBP3	-0.430078326	0.001185826	0.027611899
SYT5	-0.430461379	0.002235107	0.041224498
B9D1	-0.432810433	0.00135322	0.030052382
USO1	-0.432872777	0.000814007	0.02177818
TP53I13	-0.43863385	0.000488016	0.015941105
P2RX4	-0.439362102	0.000191147	0.008660826
LONRF1	-0.440986752	0.00094513	0.023962622
C9orf3	-0.441101619	5.23478E^{-05}	0.003363343
MYH14	-0.441160929	3.9524E^{-05}	0.002832422
NPY6R	-0.442195208	0.000343149	0.012648053
APBB3	-0.442865089	0.002713247	0.046394763
LHX6	-0.442938229	0.001748151	0.036056568
TBCEL	-0.443275671	3.21496E^{-05}	0.002467617
AHRR	-0.444146743	0.002570672	0.045134575
TAP1	-0.44446719	0.000676081	0.0194197
SIRT2	-0.445068737	3.89371E^{-05}	0.002808317
XYLT2	-0.445515811	0.000255038	0.010324939
DPF3	-0.445592814	0.002714289	0.046394763
PCBD1	-0.445972248	0.000737199	0.020586655
FAM129B	-0.446463298	0.001139095	0.027026409

ANGPTL2	-0.446780249	$3.24733E^{-05}$	0.002478914
C4orf19	-0.44738555	0.001792654	0.036616382
KRBOX4	-0.447754187	0.000658475	0.019211693
**MYLK**	-0.448741576	0.002559109	0.044987799
RILPL1	-0.448791742	$5.54767E^{-05}$	0.003507173
DAAM2	-0.448823242	0.000759626	0.020880059
EFNA1	-0.449256838	$2.78609E^{-05}$	0.00218133
SHISA4	-0.450339606	0.001459243	0.031581714
DNAL1	-0.450889309	$4.96028E^{-05}$	0.003225562
IL1RAP	-0.451197949	0.0012564	0.028563054
AAED1	-0.452243944	0.000329884	0.012389189
HERC6	-0.452249513	0.000132256	0.006658328
PARVB	-0.453214539	0.001175283	0.027467601
LDLRAD3	-0.453764279	$2.77678E^{-06}$	0.000419383
SMAD6	-0.453943356	0.001636613	0.034453012
POU3F2	-0.455378468	0.002748036	0.04690026
SAMHD1	-0.455829418	0.000104038	0.005686039
L3MBTL3	-0.455968479	0.002715114	0.046394763
GLIPR1	-0.456582849	0.000736233	0.020586655
CSRNP2	-0.456747361	$4.60803E^{-05}$	0.003096864
FAHD2B	-0.457057508	0.000884093	0.022855684
SLC44A5	-0.458087791	0.001069515	0.026262955
KLF10	-0.458898715	0.001542554	0.032978264
IRX5	-0.459053565	0.00194262	0.037931801
CLPP	-0.459610241	0.00018959	0.008646033
INAFM1	-0.460039056	0.000700343	0.019993948
ADSSL1	-0.460356856	0.000824854	0.021933556
TCTA	-0.460511768	$1.18385E^{-05}$	0.001190671
WNT11	-0.461732135	$1.13095E^{-07}$	$2.88825E^{-05}$
DOCK5	-0.463175019	0.002553757	0.044949964
FOSL2	-0.464421163	0.000148367	0.007209639
KNDC1	-0.465194962	0.000201941	0.008934482

PCDHGC3	-0.466497551	0.000224596	0.00959315
**HDAC9**	-0.469822757	$2.33889E^{-07}$	$5.38559E^{-05}$
TRAM2-AS1	-0.470487559	0.000282671	0.011121547
TTC19	-0.470897323	$7.4093E^{-06}$	0.000874546
SMIM14	-0.471037679	$4.02493E^{-05}$	0.002855259
NUDT11	-0.471908918	$2.69614E^{-06}$	0.00041163
MXD4	-0.472438498	0.000361208	0.01297738
TGM2	-0.472892189	0.001389601	0.030545121
ADAM15	-0.473350894	0.000283745	0.011132614
KCND1	-0.473951944	0.000854641	0.02247993
DACT1	-0.474412177	0.00012272	0.006407899
QSOX1	-0.475206567	$7.63237E^{-06}$	0.000888728
RABGAP1L	-0.476639265	0.000110265	0.005910731
ABHD14B	-0.476891423	2.25443E-05	0.001907572
ESRRA	-0.477343823	0.002469352	0.044071809
TPRKB	-0.478062461	$8.74214E^{-05}$	0.00509511
GYLTL1B	-0.481037402	0.000528997	0.016848739
MBNL2	-0.481543963	0.000119296	0.006296857
TNFRSF19	-0.482114792	0.000614527	0.018411192
CERS4	-0.483640624	0.002654823	0.046141256
RAB23	-0.485770897	0.000245808	0.010095359
MB	-0.488054518	0.000649363	0.019121485
DENND6B	-0.489852192	0.001047968	0.02591506
CIART	-0.490854722	0.002910108	0.048777297
GALK1	-0.491300999	0.000460737	0.015227088
TTC39A	-0.492479143	0.001998933	0.03862816
MGLL	-0.492959515	0.001822311	0.036920417
BLOC1S3	-0.493563393	0.000659265	0.019211693
EFNA3	-0.494113251	0.001302777	0.029419295
PSMG3-AS1	-0.496013582	0.000147745	0.007209639
PNPO	-0.496508691	$7.09397E^{-07}$	0.000144408
RFWD2	-0.496614603	$1.02787E^{-07}$	$2.6736E^{-05}$

CNTNAP1	-0.496626485	0.000293817	0.011328988
TXNDC5	-0.497248349	0.000292784	0.011328988
SECISBP2L	-0.499016584	1.18677E^{-05}	0.001190671
PRKAG2	-0.500425098	0.000506888	0.016367228
LMO2	-0.501075181	0.000540769	0.016964399
PER2	-0.501854023	0.00084011	0.022246853
ARHGEF10L	-0.501966897	0.000277721	0.010957493
CTXN1	-0.504310051	0.002684638	0.046341781
SSC4D	-0.504705508	0.000361253	0.01297738
CTDSPL	-0.505037045	2.98624E^{-06}	0.00044622
FLT3LG	-0.505417018	0.001729865	0.035731892
ASB13	-0.505460723	1.43828E^{-06}	0.000262365
UNC93B1	-0.506966898	0.00077561	0.021236287
HSPB8	-0.50782772	1.94349E-06	0.000331711
CPLX1	-0.507831696	0.000931627	0.023705862
PXYLP1	-0.510843522	0.000957283	0.024045625
MME	-0.511434533	1.98407E^{-06}	0.000331764
CLEC11A	-0.511806905	0.001371536	0.0302427
TMEM221	-0.512165246	0.002281395	0.041781801
PINK1-AS	-0.513259404	0.000418885	0.014414836
CTSS	-0.514547634	0.000237733	0.009861816
GABBR2	-0.515138195	0.001117599	0.026879785
GRIPAP1	-0.515226466	9.31359E^{-09}	3.4426E^{-06}
**PHEX**	-0.515275715	0.002610602	0.045494441
TBX2	-0.517520977	0.001183925	0.027611899
FLOT2	-0.519774259	6.19161E^{-06}	0.000762872
MAPK8IP2	-0.520932468	0.000652341	0.019128984
PER1	-0.521149449	0.000952188	0.024045625
CRY2	-0.521221596	9.67601E^{-05}	0.005445493
ST7-AS1	-0.521456062	0.002416455	0.04362664
ISG20	-0.523027157	0.001244966	0.028439355
SNTA1	-0.524779364	5.16123E-05	0.003340765

**EIF4E3**	-0.525059766	$4.15459E^{-06}$	0.000583553
LOC101926913	-0.525196407	0.000520646	0.016658293
AFP	-0.525295041	0.002421064	0.04365375
**POU6F1**	-0.527057757	$7.46254E^{-09}$	$2.99482E^{-06}$
CDH8	-0.528622693	0.001855984	0.037030036
TUBB3	-0.528657927	0.000805557	0.021634524
TMEM56	-0.528868206	$8.26787E^{-06}$	0.000936536
TSPAN33	-0.530707302	0.001550422	0.03306235
RANBP10	-0.530816555	$2.76342E^{-08}$	$8.43804E^{-06}$
IGFBP2	-0.531165496	$3.40006E^{-05}$	0.002553861
TAPBPL	-0.53124793	0.000721302	0.020262828
**ADGRG1**	-0.532216366	0.001755702	0.036159217
MIEF1	-0.533321369	$1.09594E^{-08}$	$3.75451E^{-06}$
FAM213B	-0.534547823	0.000292254	0.011328988
SHANK1	-0.534613458	0.000157626	0.007505154
CCDC96	-0.534646991	0.000780799	0.021295342
MERTK	-0.537425316	$3.33961E^{-06}$	0.000483589
TBC1D2B	-0.538679627	$1.48955E^{-05}$	0.001404175
LRFN4	-0.539632993	0.001195081	0.027631882
GRIK2	-0.540576353	$6.71169E^{-06}$	0.000812693
CXCL16	-0.54132552	$4.20633E^{-05}$	0.002910448
FAS	-0.541598744	0.000295202	0.011328988
FZD7	-0.542524738	$2.55075E^{-07}$	$5.77867E^{-05}$
GNB2	-0.542578021	$6.3959E^{-06}$	0.00078119
SLC48A1	-0.542982391	$6.99806E^{-06}$	0.000840126
APC2	-0.543614914	0.000449986	0.014906842
HOXD-AS2	-0.546024791	0.002193845	0.040952756
UNC13A	-0.551100298	$1.07003E^{-05}$	0.001105119
GPC1	-0.5520851	$1.37384E^{-05}$	0.001321705
CSF1	-0.552130403	0.002500946	0.044434417
KCNIP3	-0.552860149	0.002443454	0.043852496
SLC2A1	-0.554087991	$1.0519E^{-05}$	0.001102607

CHST3	-0.554343781	0.002018013	0.038775657
SPARCL1	-0.554480604	0.001890658	0.03738556
DACT3	-0.554858619	0.000106022	0.005749612
CRIP2	-0.555167579	0.002356449	0.042770232
PTPRG	-0.555659485	4.15483E-05	0.002890941
FOXP4	-0.556585285	4.95512E-05	0.003225562
IGFBP3	-0.557963659	0.001046097	0.025914426
KIAA0040	-0.558028607	7.24318E-05	0.004360776
APCDD1	-0.559043297	4.81746E-05	0.003176808
PTX4	-0.560636385	0.002144215	0.040426363
CGREF1	-0.561098532	0.001131474	0.026982488
CCND1	-0.563235341	0.001842192	0.037017786
MYCBP	-0.56355763	0.000761487	0.020890338
TRIM7	-0.564788406	0.001838457	0.037017786
LOXL1-AS1	-0.566284789	0.001832078	0.037017786
GFM1	-0.566680011	7.13442E-08	1.9649E-05
IGF1	-0.567381762	0.001714399	0.035506783
GAP43	-0.569488661	0.00160848	0.034025175
DOCK9-AS2	-0.569547846	0.000870664	0.022658038
MAOB	-0.569577918	0.00047046	0.015475587
CTNNA2	-0.570114945	0.001305527	0.029422339
SLC35F3	-0.570302281	0.000784671	0.021316165
ARID5B	-0.572716739	2.34875E-06	0.00037082
KLK4	-0.575010363	0.001356082	0.030052382
CASC15	-0.575365078	0.001605175	0.034006473
C10orf54	-0.578047152	0.001173755	0.027467601
FIBCD1	-0.584961174	0.000129909	0.006563669
IRF9	-0.586607695	1.81901E-05	0.001602172
KATNB1	-0.587679086	2.34339E-09	1.21908E-06
MESDC2	-0.589824252	4.50571E-08	1.30969E-05
GFRA2	-0.590776308	0.001127405	0.026941873
MAP1A	-0.592651962	0.000255808	0.010324939

SLC2A13	-0.595761746	$2.37604E^{-06}$	0.00037082
GPC6	-0.598308337	0.000604013	0.018166948
PPM1L	-0.599206249	0.000877182	0.022774298
HLF	-0.6008902	0.000747278	0.020727779
KCNN1	-0.601876415	$2.79538E^{-05}$	0.00218133
ISM1	-0.602154848	$7.80598E^{-05}$	0.004606841
MITF	-0.605389764	$8.75823E^{-06}$	0.000976334
**PYGM**	-0.606721761	0.000896577	0.023064682
LACC1	-0.610835938	0.000826061	0.021933556
LOC101926911	-0.61343965	0.000127576	0.00649253
SYT14	-0.613459264	$7.12015E^{-05}$	0.00431076
PPL	-0.614089986	0.000374362	0.013312127
BTG3	-0.614376638	$5.70678E^{-06}$	0.000722139
ICAM5	-0.619051184	$9.69225E^{-05}$	0.005445493
POGLUT1	-0.619579346	$8.26535E^{-09}$	$3.13771E^{-06}$
MDM2	-0.619919793	$2.64074E^{-06}$	0.000407603
HAR1A	-0.620325604	0.000435096	0.014737258
SPTLC3	-0.621175966	$3.38951E^{-05}$	0.002553861
DIRAS1	-0.623610456	0.000623362	0.01862925
LOC101928069	-0.624868806	0.000380729	0.013436491
LRP12	-0.626073294	$2.04314E^{-07}$	$4.94792E^{-05}$
NTF4	-0.628437895	0.000489997	0.01596866
RBM20	-0.633123092	0.000417735	0.014414836
SESN3	-0.640332205	$9.32273E^{-05}$	0.005350735
DBNDD1	-0.64140089	$1.06332E^{-05}$	0.001105119
CPLX2	-0.641549831	0.000307053	0.01165641
LIN7B	-0.642535312	$2.21336E^{-05}$	0.001884172
GRIN3A	-0.642763318	$9.32718E^{-06}$	0.001017778
ADRA2C	-0.651600611	$6.44624E^{-05}$	0.003971223
PRICKLE1	-0.652119732	$4.38332E^{-06}$	0.000607863
**LOC100129550**	-0.653117052	$1.94877E^{-06}$	0.000331711
DTNA	-0.658747242	$1.197E^{-07}$	$3.00234E^{-05}$

FAH	-0.659241708	$1.07275E^{-09}$	$6.27828E^{-07}$
IFI44L	-0.66027456	$1.32697E^{-06}$	0.000248515
ZBTB34	-0.660389866	$8.54485E^{-07}$	0.000169044
PCDHB17P	-0.66322077	0.000116194	0.006158694
PLEKHA4	-0.667929614	$1.62892E^{-05}$	0.001476118
JAK3	-0.668934613	$3.53464E^{-05}$	0.002626851
NDUFB5	-0.671842934	$4.33263E^{-09}$	$1.90176E^{-06}$
LIMK1	-0.676718342	$3.80553E^{-12}$	$4.85932E^{-09}$
RHOU	-0.680751199	$7.69899E^{-07}$	0.000154486
C9orf173-AS1	-0.681476563	$4.75E^{-05}$	0.003147097
SLIT3	-0.684192819	$4.54709E^{-07}$	$9.82592E^{-05}$
CALU	-0.689253472	$4.5689E^{-08}$	$1.30969E^{-05}$
HOXC8	-0.689591349	0.000148853	0.007209639
CEMIP	-0.692950698	$6.74769E^{-05}$	0.004107683
CPM	-0.694065784	$4.7039E^{-07}$	0.000100107
USP2	-0.694756026	$9.63431E^{-05}$	0.005445493
COL26A1	-0.696940708	$7.66294E^{-06}$	0.000888728
KREMEN2	-0.700134104	$3.89877E^{-05}$	0.002808317
PIGZ	-0.701117173	$2.57706E^{-05}$	0.002072238
FSCN1	-0.704413338	$5.7145E^{-09}$	$2.36076E^{-06}$
KIAA1143	-0.706735395	$2.0098E^{-11}$	$2.01641E^{-08}$
SLC39A14	-0.710947721	$2.69494E^{-11}$	$2.52354E^{-08}$
CHRD	-0.714928435	$5.66147E^{-05}$	0.003534264
IL15RA	-0.716248903	$3.99644E^{-05}$	0.002849439
GEMIN7	-0.721382804	$4.57366E^{-09}$	$1.94672E^{-06}$
PPP1R1A	-0.736878442	$4.36619E^{-05}$	0.002977064
PTCHD4	-0.747878645	$5.49169E^{-06}$	0.000701239
EFHD2	-0.752470311	$5.79345E^{-11}$	$4.78675E^{-08}$
SYNC	-0.754622598	$2.388E^{-05}$	0.002008492
ISOC2	-0.758205452	$9.80078E^{-09}$	$3.52979E^{-06}$
L3MBTL4	-0.777717184	$1.88771E^{-05}$	0.001636718
NFE2L3	-0.783951384	$3.45681E^{-10}$	$2.28256E^{-07}$

SIX4	-0.786068433	$1.03296E^{-08}$	$3.62725E\text{-}^{06}$
LINGO1	-0.799633257	$4.41472E^{-10}$	$2.69605E^{-07}$
RABEPK	-0.800196329	$7.90425E^{-09}$	$3.08398E^{-06}$
UAP1	-0.824043651	$8.3071E^{-13}$	$1.29646E^{-09}$
BASP1	-0.824097946	$1.42052E^{-09}$	$7.98106E^{-07}$
CDK15	-0.8422545	$1.07177E^{-11}$	$1.158E^{-08}$
ADAM19	-0.846120742	$1.30861E^{-06}$	0.000248388
GPR1	-0.853316906	$7.60446E^{-08}$	$2.05408E^{-05}$
PLXNB3	-0.870070489	$2.72313E^{-09}$	$1.36546E^{-06}$
KIAA1462	-0.878292752	$2.2431E^{-07}$	$5.25109E^{-05}$
MYRIP	-0.897493144	$1.07803E^{-12}$	$1.5142E^{-09}$
ANKFN1	-0.899481869	$5.75088E^{-07}$	0.000118789
SEMA6B	-0.907557468	$2.09422E^{-08}$	$6.68534E^{-06}$
USMG5	-0.941816931	$1.14731E^{-18}$	$5.37173E^{-15}$
USH1C	-0.962877589	$1.21873E^{-07}$	$3.00321E^{-05}$
NAT16	-0.972059149	$4.3972E^{-08}$	$1.30969E^{-05}$
RHBG	-1.013298074	$2.49676E^{-08}$	$7.79321E^{-06}$
METTL9	-1.030072641	$3.39804E^{-14}$	$6.81842E^{-11}$
HS3ST3A1	-1.051996657	$5.1111E^{-11}$	$4.48691E^{-08}$
RNF11	-1.192395522	$6.74941E^{-22}$	$9.48022E^{-18}$
**NNT**	-1.335737567	$2.08051E^{-13}$	$3.65286E^{-10}$

Supplementary Table 3B

**Gene**	**$\log_2$ Fold Change KD shRNA vs SCR shRNA**	**p value**	**q value**
PAG1	0.922963685	$3.59096E^{-07}$	$1.18855E^{-12}$
LRRC19	0.612820719	$1.93025E^{-06}$	$1.18855E^{-12}$
FLJ36777	0.53515861	0.002720375	0.000102951
MIR30A	0.491980816	$2.16694E^{-05}$	$6.25969E^{-11}$
MAP3K7CL	0.455497596	0.008864981	0.001233905
AGPAT9	0.426596178	0.005470306	0.000437182

TOB1-AS1	0.424577398	0.012176789	0.002685871
LOC102546294	0.416840189	0.015880389	0.004944718
HNRNPH2	0.416450686	0.015703784	0.004922055
MRVI1	0.38349567	0.028211527	0.014027222
OXCT1-AS1	0.377560899	0.028736809	0.014536857
DNAH17-AS1	0.376939137	0.038228442	0.023347948
LOC101927482	0.37279889	0.029301462	0.015139944
TMEM27	0.36469166	0.034590982	0.020564754
SNORD56	0.361375391	0.013589884	0.003519753
ZMYND12	0.360175385	0.039033123	0.02415184
RPP21	0.35779367	0.014347098	0.004064221
CFD	0.356412208	0.050482853	0.037597227
EVI2A	0.355319362	$5.46757E^{-05}$	$9.94986E^{-10}$
DHRS2	0.349121289	0.052839644	0.040072093
KLHL29	0.347924949	0.041412922	0.027579562
LOC100506023	0.347404148	0.055390582	0.043898698
LOC100288842	0.336063684	0.022815615	0.009868025
PRIMA1	0.33263901	0.050862553	0.037849115
GIPC2	0.318119744	0.057966443	0.047702294
MBOAT1	0.317049219	0.044522848	0.03114562
KCNN2	0.316723437	0.019130807	0.007161549
IFI27L2	0.314031426	0.030406681	0.015796429
LOC100128568	0.309623899	0.040639045	0.026388408
LUM	0.307710987	0.029093526	0.014950075
SPATA12	0.305674434	0.054742649	0.042903917
ZBTB42	0.304157315	0.037958504	0.023347948
DENND5B-AS1	0.299255963	0.016876204	0.005352566
C15orf38-AP3S2	0.293687827	0.032084415	0.017576687
CTF1	0.287930437	0.058573172	0.048541296
LACTB2-AS1	0.283455424	0.005183491	0.000419307
**CIDEB**	0.274631471	0.023699386	0.010331414
RPL41	0.269248114	0.030030805	0.015538175

LRP1B	0.266774653	0.051107393	0.038006822
LINC01108	0.262559086	0.055193056	0.043631188
LOC439994	0.259761615	0.040718778	0.026388408
SPRYD4	0.257010204	0.015764599	0.004922055
SLC39A11	0.254822509	0.055682845	0.044162631
MCTP1	0.251502657	0.018877004	0.006980542
PALMD	0.250883466	0.050958639	0.037849115
SYP	0.25037673	0.040671083	0.026388408
ULBP1	0.243470563	0.053773876	0.041491298
**NEK7**	0.242325668	0.041951421	0.028489075
OXCT1	0.236869688	0.044214553	0.03114562
UBE2V1	0.235145801	0.02327178	0.010230316
GCNT1	0.222076804	0.052755223	0.040043735
IPP	0.219147644	0.03821867	0.023347948
**EIF4E3**	0.219037133	0.043522905	0.030636982
**DHFRL1**	0.216899876	0.022378488	0.009536317
**H1F0**	0.216390215	0.0391133	0.02415184
STX10	0.215862429	0.033900656	0.019652053
MVB12A	0.210255131	0.057932256	0.047702294
LIAS	0.206217317	0.051565446	0.03853314
CFDP1	0.205600558	0.017917354	0.006058174
FUT11	0.205440062	0.053139102	0.04041915
LINC01249	0.204685473	0.053288466	0.040592472
RPUSD3	0.202870628	0.027084724	0.012975545
TCN1	0.20167018	0.005110315	0.000419307
C15orf27	0.190901221	0.026425217	0.012638337
**C9orf40**	0.18995916	0.025902497	0.012262875
TMEM101	0.185293794	0.047508275	0.033310378
SLC25A48	0.181902166	0.026917564	0.012892514
SPTY2D1	0.17734588	0.020252885	0.007525648
LCLAT1	0.176846194	0.048698723	0.035028452
**CDCA4**	0.174916638	0.046324217	0.032695147

NSUN3	0.174118623	0.035415164	0.021442113
LRRC7	0.173789434	0.015245721	0.004612919
RAB13	0.163267759	0.047484968	0.033310378
DHFR	0.161618689	0.042633026	0.029431019
MYO1D	0.159225446	0.039384463	0.024470139
EIF3J-AS1	0.1583085	0.058081061	0.047702294
FAM20B	0.145125488	0.045864587	0.032322417
LGALS3	0.138138996	0.028227591	0.014027222
**G3BP2**	0.137087002	0.026800165	0.012892514
ITGAX	0.130955312	0.055704246	0.044162631
MRPL22	0.12803647	0.054501503	0.042680859
C2orf88	0.120676356	0.02555943	0.012009553
C6	0.100356484	0.048832167	0.035155931
KRT38	0.097390291	0.043871246	0.030636982
LOC101927926	0.095872076	0.038189467	0.023347948
LINC01215	0.088617824	0.049360066	0.035965553
LOC101927285	-0.09961036	0.022599791	0.009704973
LOC100144595	-0.133631587	0.04441406	0.03114562
ZC3HC1	-0.1337447	0.050313073	0.037546911
ANKRD62P1-PARP4P3	-0.137498722	0.048598705	0.034971679
NOMO1	-0.139838945	0.049835564	0.036670996
OPCML	-0.140331825	0.053833259	0.041491298
WDR45	-0.141400001	0.043695613	0.030636982
ANKRD10	-0.142295751	0.038491433	0.023598529
CTNNA1	-0.145772443	0.045649344	0.032234797
**POU6F1**	-0.159009836	0.059033235	0.049007404
EHD4	-0.165197365	0.037459267	0.023234751
ZXDC	-0.167856226	0.043785279	0.030636982
WDR19	-0.180732699	0.046884277	0.03277974
LOC650226	-0.184870592	0.037345064	0.02317222
COTL1	-0.187295152	0.046150649	0.032491318

NGF	-0.190570223	0.046796916	0.032751312
**HDAC9**	-0.194872835	0.019944616	0.007280366
LINC01461	-0.200178236	0.047344414	0.033310378
**TSTD2**	-0.203090059	0.000492572	7.51596E-07
LOC101928307	-0.206526135	0.004306283	0.000342047
PRG2	-0.208843565	0.016935882	0.005352566
PDK3	-0.20901309	0.054342343	0.042481722
FAM124B	-0.213022439	0.036047545	0.022101759
C11orf45	-0.214602111	0.046406016	0.032709644
TXK	-0.215703629	0.037241585	0.02317222
NKTR	-0.215780944	0.036561446	0.022478415
HSPB3	-0.216955329	0.050962483	0.037849115
PEAK1	-0.22095597	0.05174136	0.038760256
RPS6KC1	-0.223482955	0.008085316	0.000983621
LAMA2	-0.225480292	0.058164554	0.047731676
USP34	-0.225948829	0.045500177	0.032109909
TNRC6C	-0.226546766	0.051995203	0.039159188
PCDHB8	-0.228649076	0.058897448	0.049007404
ZNF653	-0.23155931	0.057670782	0.047405262
SLC51B	-0.23604596	0.04551301	0.032109909
PBLD	-0.239042045	0.048254642	0.034556275
COL6A2	-0.23935149	0.046040618	0.032419111
SYNE2	-0.240403854	0.036090447	0.022101759
CHADL	-0.240559921	0.04541016	0.032109909
PARVA	-0.241122296	0.037814418	0.023347948
PLEKHH1	-0.241169948	0.055957206	0.044204248
CEP95	-0.244337751	0.01790992	0.006058174
PCDHGB4	-0.244656367	0.059318509	0.049482729
SRGAP1	-0.244692824	0.052208547	0.039470544
LOC146880	-0.247626908	0.046673166	0.032751312
DNM3OS	-0.248968848	0.004924791	0.000419307
DYNC2H1	-0.250011021	0.05602147	0.044204248

UTRN	-0.251506423	0.051211838	0.038076921
SEMA3G	-0.252903462	0.026571066	0.012724545
AEBP1	-0.253179042	0.029844498	0.015538175
IPW	-0.253514177	0.016844343	0.005352566
DPY19L2	-0.254920522	0.024091969	0.01061904
LINC01239	-0.255127158	0.050686855	0.037849115
LTBP1	-0.256529094	0.004672361	0.000413142
SPP1	-0.257970302	0.038021365	0.023347948
**LOC100129550**	-0.258779822	0.047386083	0.033310378
FAM117B	-0.258976801	0.008040006	0.000983621
L3MBTL1	-0.26190691	0.045796234	0.032322417
ZNF208	-0.262893055	0.023930888	0.010520777
VPS13D	-0.263240714	0.050747736	0.037849115
CLCN6	-0.266043675	0.055202928	0.043631188
RBMS3	-0.269945181	0.014721024	0.004308479
PCDHA9	-0.270198906	0.021332561	0.008572439
LOC440982	-0.271264841	0.032967571	0.018687829
FLRT2	-0.271608743	0.053002981	0.040275599
KIAA0226L	-0.274653216	0.049724002	0.036588427
PPP3R2	-0.274674803	0.059051084	0.049007404
CHSY3	-0.275838137	0.055733761	0.044162631
ACTA2	-0.277633844	0.04309727	0.030007826
TPTE	-0.278423442	0.037089502	0.023147156
HUWE1	-0.279358357	0.058017492	0.047702294
CYP2E1	-0.280986411	0.052475621	0.039584269
COL6A1	-0.283183834	0.054641467	0.042836561
LOC101928323	-0.283644537	0.058917263	0.049007404
CD101	-0.284007048	0.048574916	0.034971679
MRPS31P5	-0.285703667	0.036368263	0.02242285
RYR2	-0.286100951	0.023638099	0.010331414
COLQ	-0.287357308	0.038521567	0.023598529
DMD	-0.288680924	0.044780203	0.031386013

POU2F1	-0.288917145	0.031709068	0.017233977
KALRN	-0.290277332	0.036109577	0.022101759
AFG3L1P	-0.291907712	0.030630892	0.015796429
ZNF286B	-0.29331278	0.037684631	0.023347948
HERC2P4	-0.295624049	0.042488458	0.029411855
FRMD3	-0.296504278	0.003677761	0.000229949
RNF213	-0.296639061	0.046690641	0.032751312
LINC00310	-0.296837674	0.016458615	0.005234614
GSDMB	-0.296872076	0.057378817	0.047050854
LPXN	-0.297268286	0.016236562	0.005109174
SVEP1	-0.297287351	0.03730122	0.02317222
IDH1-AS1	-0.297363747	0.043680474	0.030636982
OR2F1	-0.2979349	0.023657058	0.010331414
HBEGF	-0.298871268	0.047210145	0.033299029
HERC1	-0.300714423	0.04797921	0.034133625
MIR600HG	-0.303578674	0.046564236	0.032751312
RAB31	-0.304570057	0.033106877	0.018723435
DYNC1H1	-0.305061922	0.046752173	0.032751312
STARD9	-0.307091184	0.024434833	0.010827321
C1orf167	-0.307892525	0.043017164	0.030005596
HECTD4	-0.310726695	0.026128514	0.012460571
KLHL4	-0.311214425	0.033834649	0.019652053
CFAP44	-0.311348547	0.013153606	0.003320399
LOC101927391	-0.313651651	0.056568122	0.045306176
GRK4	-0.318791089	0.016912	0.005352566
SCN1A	-0.32072469	0.031019005	0.016270811
GPR75	-0.32264214	0.04591856	0.032322417
ZDHHC11	-0.323069891	0.004917445	0.000419307
ACP5	-0.323346927	0.024506614	0.010827321
TRPV1	-0.323909404	0.008039478	0.000983621
ATP10A	-0.325064816	0.033438973	0.019144679
FAT1	-0.325729481	0.029861134	0.015538175

CXCL14	-0.328423567	0.044586412	0.03114562
LINC00202-2	-0.328472898	0.030142058	0.015542747
FLG-AS1	-0.331614925	0.055938407	0.044204248
DST	-0.332088409	0.010518958	0.001841208
RASGRF2	-0.332120848	0.040235519	0.025911685
ADGB	-0.334430061	0.005038477	0.000419307
IGFALS	-0.334742154	0.039133537	0.02415184
SMG1	-0.334968335	0.019855891	0.007280366
PCDH17	-0.337204457	0.025635549	0.012009553
USP32P2	-0.339749701	0.019406971	0.007163797
CLEC2D	-0.339829939	0.017178205	0.005501014
DGKH	-0.340297155	0.052397283	0.039569596
LOC100506124	-0.340482209	0.049108883	0.035586716
GAB3	-0.342672996	0.045391115	0.032109909
PDE5A	-0.343363181	0.005647091	0.000447311
PDLIM1	-0.343597663	0.055790637	0.044162631
**MYLK**	-0.343879737	0.005415077	0.000437182
PTOV1-AS1	-0.343883113	0.03524978	0.021395879
CRIPAK	-0.344547847	0.022252749	0.009490262
MUC20	-0.345473344	0.019222985	0.007163018
SCN9A	-0.34662102	0.057466049	0.047087575
SGK494	-0.347473714	0.035307024	0.021395879
FBXL21	-0.34889163	0.005012418	0.000419307
PKI55	-0.349264393	0.05046487	0.037597227
PTCH2	-0.349775682	0.045009063	0.031699682
ZNF540	-0.351148848	0.042656233	0.029431019
SRRM4	-0.351663107	0.011305939	0.002192449
CEBPD	-0.352917759	0.044471066	0.03114562
SFRP4	-0.353364453	0.052372546	0.039569596
HCN1	-0.354724925	0.032065205	0.017576687
MTTP	-0.355403608	0.051354664	0.038230752
IL4I1	-0.356932511	0.026270991	0.012540813

DNHD1	-0.359480179	0.021282429	0.008572439
**ADGRG1**	-0.360549493	0.027851612	0.013843904
RUNX1T1	-0.361741635	0.005392784	0.000437182
SNRK-AS1	-0.361980713	0.038188926	0.023347948
SMIM17	-0.362330777	0.033003746	0.018687829
FAR2P1	-0.364086639	0.044433552	0.03114562
LYG1	-0.365128345	0.029806655	0.015538175
HMCN1	-0.36587039	0.035100904	0.021323169
FMN1	-0.367719966	0.043874627	0.030636982
HNRNPU-AS1	-0.367751957	0.026942619	0.012892514
IFITM2	-0.37206973	0.024869464	0.011196374
MAP2	-0.372274477	0.036921253	0.022987099
DNAJC15	-0.372886745	0.0389168	0.02415184
KLKB1	-0.373000009	0.029894185	0.015538175
RUNX1	-0.374529132	0.030605355	0.015796429
AGT	-0.376065563	0.019921807	0.007280366
**PHEX**	-0.37647032	0.024284102	0.010758912
THSD1	-0.376631368	0.008257927	0.001020541
KHDC1	-0.378596959	0.038149179	0.023347948
NEB	-0.37884558	0.033995357	0.019674258
**PYGM**	-0.380270814	0.036455789	0.022433902
GALNT3	-0.380624629	0.030057531	0.015538175
PCDHGA10	-0.382438236	0.036036303	0.022101759
CFAP47	-0.384093868	0.032332408	0.017847704
MARCH10	-0.3855816	0.027942978	0.013854415
ACTG2	-0.391306317	0.03049252	0.015796429
UG0898H09	-0.396061696	0.028402001	0.014161905
LOC102724890	-0.397039577	0.027825104	0.013843904
LINC01337	-0.401564459	0.019327656	0.007163797
IL17B	-0.403321609	0.013344278	0.003398214
AGPAT4-IT1	-0.407989736	0.014814628	0.004310088
FN3K	-0.410044666	0.023076858	0.010091562

NELL2	-0.412183847	0.006494158	0.00061319
ZNF90	-0.413760099	0.023445697	0.010331414
SEMA6A-AS1	-0.417981728	0.011056796	0.002094253
LINC01004	-0.418332894	0.019578957	0.007163797
SLC13A5	-0.418477488	0.021914729	0.009176936
MUCL1	-0.418850026	0.007850538	0.000983621
NPIPB4	-0.419435333	0.005639869	0.000447311
RHPN1-AS1	-0.426272458	0.019548344	0.007163797
PITX2	-0.428057386	0.016078399	0.00504525
LRRC3B	-0.428588228	0.00926441	0.001375844
ADGRV1	-0.436666903	0.000271548	$1.3211E^{-07}$
GTSF1	-0.442421232	0.006004992	0.000520865
UNC5C	-0.464547124	0.01048731	0.001841208
FN1	-0.46740212	0.003703526	0.000229949
FAT4	-0.471025872	0.009823221	0.00156647
PCDHGA5	-0.473251816	0.009544251	0.001469787
LRRC70	-0.474788669	0.008088762	0.000983621
S100A10	-0.481139441	0.006491744	0.00061319
SPTBN5	-0.485013534	0.007871563	0.000983621
MSH5-SAPCD1	-0.490386257	0.006543624	0.00061319
KCND2	-0.517020462	0.001161919	$9.70578E^{-06}$
KCNQ1OT1	-0.55680171	0.002258799	$6.29538E^{-05}$
NANOS1	-0.568628778	0.001750595	$3.13104E^{-05}$
FAM133CP	-0.578109116	$1.52287E^{-05}$	$2.47218E^{-11}$
PEX5L	-0.855451164	$4.604E^{-09}$	$1.18855E^{-12}$
**NNT**	-1.65327293	$1.24657E^{-19}$	$1.18855E^{-12}$

Supplementary Table 4. A-B: Significantly (p<0.01) dysregulated pathways comparing KD siRNA cells to SCR siRNA cells (4A) and KD shRNA cells to SCR shRNA cells (4B). C: Gene expression changes within the 'Protein Processing in the Endoplasmic Reticulum' KEGG pathway, comparing KD shRNA cells to SCR shRNA cells.

Supplementary Table 4A

Pathway (KD siRNA vs SCR siRNA)	p value	q value	up-regulated genes	down-regulated genes
hsa03040 Spliceosome	$5.42948E^{-06}$	0.000782253	94	-32
hsa03010 Ribosome	$7.59468E^{-06}$	0.000782253	97	-29
hsa03013 RNA transport	$1.51944E^{-05}$	0.00104335	105	-36
hsa04115 p53 signaling pathway	0.000170523	0.015091608	30	-32
hsa04010 MAPK signaling pathway	0.000171555	0.015091608	109	-111
hsa04360 Axon guidance	0.000219781	0.015091608	69	-68
hsa04710 Circadian rhythm	0.000815962	0.042022043	13	-15
hsa04110 Cell cycle	0.000957167	0.049294082	79	-39
hsa04114 Oocyte meiosis	0.001315026	0.051042169	65	-30
hsa03008 Ribosome biogenesis in eukaryotes	0.001486665	0.051042169	52	-18
hsa03015 mRNA surveillance pathway	0.002031137	0.059773455	54	-25
hsa04150 mTOR signaling pathway	0.00259412	0.06679858	85	-45
hsa04068 FoxO signaling pathway	0.001990434	0.072315727	61	-52
hsa04151 PI3K-Akt signaling pathway	0.002106283	0.072315727	118	-107
hsa04014 Ras signaling pathway	0.002689978	0.079162207	79	-71
hsa03030 DNA replication	0.006003215	0.11408099	27	-8
hsa03460 Fanconi anemia pathway	0.006150791	0.11408099	36	-12
hsa04120 Ubiquitin mediated proteolysis	0.006580001	0.11408099	89	-42
hsa03018 RNA degradation	0.006645495	0.11408099	52	-22
hsa04140 Regulation of autophagy	0.008136037	0.123597863	72	-46
hsa00970 Aminoacyl-tRNA biosynthesis	0.008399855	0.123597863	28	-16
hsa04390 Hippo signaling pathway	0.005437739	0.140021788	54	-67

Supplementary Table 4B

Pathway (KD shRNA vs SCR shRNA)	p.val	q.val	Up-regulated genes	Down-regulated genes
hsa03010 Ribosome	1.04416E-05	0.002150972	98	-34
hsa03040 Spliceosome	0.001103391	0.113235169	84	-46
hsa04141 Protein processing in endoplasmic reticulum	0.001649056	0.113235169	102	-59
hsa00983 Drug metabolism - other enzymes	0.005443889	0.229149376	25	-10
hsa00240 Pyrimidine metabolism	0.005561878	0.229149376	65	-34
hsa00190 Oxidative phosphorylation	0.00993535	0.341113698	68	-47

Supplementary Table 4C

Gene	Log2fold change KD shRNA vs SCR shRNA	p value	q value
AMFR	0.029120894	0.692680229	0.923307833
ATF4	-0.038779036	0.749485377	0.954539392
ATF6	0.011596787	0.894351109	0.994260181
ATF6B	0.021001096	0.781540675	0.976327549
ATXN3	-0.013868658	0.878327015	0.993017965
BAG1	0.036172831	0.763954601	0.963331979
BAG2	0.094784169	0.585576621	0.83898096
BAK1	0.086045716	0.403960204	0.611763447
BAX	0.154032462	0.118575863	0.145116226
BCAP31	0.051863741	0.531856228	0.774625038
BCL2	0.264732307	0.063414339	0.054909291
CALR	0.041508948	0.633471971	0.8781132
CANX	0.062913422	0.409500979	0.619405643
CAPN1	-0.000619529	0.994582708	1
CAPN2	0.062333279	0.472683232	0.703888616
CKAP4	0.033468944	0.754032405	0.955323467
CRYAB	-0.07853826	0.66601657	0.904603198
CUL1	-0.070065107	0.416543969	0.62780944
DAD1	-0.005456425	0.947446795	1
DDIT3	0.016955951	0.925816883	1
DDOST	0.074300928	0.315038287	0.47954916
DERL1	0.02177722	0.761535097	0.961247176
DERL2	0.02130004	0.747894859	0.953932446
DERL3	0.125943444	0.483678768	0.716996125

DNAJA1	0.026895612	0.846239555	0.993017965
DNAJA2	0.034288294	0.653996117	0.895577112
DNAJB1	0.019377577	0.855982505	0.993017965
DNAJB11	0.045474053	0.573816699	0.826548807
DNAJB12	-0.051777694	0.557169975	0.806207111
DNAJB2	0.127391544	0.210599576	0.29968158
DNAJC1	0.208341418	0.071374842	0.063696747
DNAJC10	-0.03392065	0.6074001	0.859987994
DNAJC3	0.03564692	0.638573891	0.88182883
DNAJC5	0.058052695	0.373352077	0.563225419
DNAJC5B	-0.005691453	0.841974099	0.993017965
DNAJC5G	0.028958158	0.753369557	0.95466863
EDEM1	-0.029555307	0.779016203	0.974083751
EDEM2	0.040029532	0.642439254	0.885189529
EDEM3	-0.033780382	0.742804483	0.95228687
EIF2AK1	-0.011811788	0.849785813	0.993017965
EIF2AK2	0.072101074	0.501691058	0.73976841
EIF2AK3	-0.050681638	0.487351497	0.720265345
EIF2AK4	0.015797672	0.825704713	0.993017965
EIF2S1	0.008872628	0.907393451	0.999456943
ERLEC1	0.034950188	0.675729634	0.913887439
ERN1	0.084514189	0.457532042	0.685291886
ERO1A	0.12521397	0.259085879	0.389646236
ERO1B	0.025408659	0.805929681	0.993017965
ERP29	0.079287044	0.462056315	0.69037553
FBXO2	-0.013466883	0.941123962	1
FBXO6	-0.045909183	0.668014318	0.905945065
GANAB	0.056723727	0.435820189	0.653217036
HERPUD1	-0.008613719	0.935825808	1
HSP90AA1	0.049457951	0.6959656	0.923307833
HSP90AB1	0.027239569	0.734677206	0.951441751
HSP90B1	0.034458995	0.698058433	0.924409218
HSPA1A	-0.006797184	0.965327355	1
HSPA1B	0.090466711	0.363409827	0.549969177
HSPA1L	0.11312329	0.443397447	0.663627153
HSPA2	-0.037981621	0.745043223	0.95228687
HSPA4L	-0.004670522	0.963538567	1
HSPA5	0.046658057	0.504077511	0.742899748
HSPA6	-0.127562115	0.412822443	0.62288389
HSPA8	0.065215999	0.638611585	0.88182883
HSPBP1	0.000887396	0.993688187	1

HSPH1	0.019896432	0.878338747	0.993017965
HYOU1	0.043969168	0.459618516	0.688000682
LMAN1	0.050698474	0.54194916	0.789328352
LMAN1L	0.139561888	0.381858555	0.577168278
LMAN2	0.120650764	0.16488796	0.220413878
MAN1A1	-0.059559597	0.689496573	0.922591082
MAN1A2	0.073376513	0.390064306	0.589383219
MAN1B1	0.035607125	0.724636496	0.943578581
MAN1C1	-0.005119397	0.97149816	1
MAP2K7	0.12186443	0.369707866	0.557481343
MAP3K5	-0.122240346	0.403909705	0.611763447
MAPK10	-0.158493298	0.177231713	0.242838509
MAPK8	-0.011912116	0.893714501	0.994107865
MAPK9	-0.017925903	0.777676134	0.97385499
MARCH6	-0.051123261	0.492734682	0.727072243
MBTPS1	-0.051666244	0.517443442	0.756981957
MBTPS2	0.079209414	0.446740087	0.667852591
MOGS	0.121293355	0.256380469	0.386111655
NFE2L2	-0.042174373	0.705215433	0.929482314
NGLY1	0.01489591	0.880649163	0.993017965
NPLOC4	-0.040005858	0.660166539	0.897674496
NSFL1C	0.083690553	0.275362568	0.41614581
OS9	0.054138039	0.476595319	0.707696985
P4HB	0.116021915	0.127071861	0.159405719
PARK2	-0.046058773	0.787567574	0.981106444
PDIA3	0.053520403	0.48750727	0.720265345
PDIA4	0.032534269	0.744295685	0.95228687
PDIA6	0.021028668	0.769691785	0.967111581
PLAA	0.035249955	0.642720433	0.885460651
PPP1R15A	0.105426696	0.470330781	0.701262454
PREB	0.001220953	0.987458323	1
PRKCSH	0.04564895	0.573342312	0.826057887
RAD23A	0.084598367	0.336763602	0.510899339
RAD23B	0.058327984	0.306234455	0.464872169
RBX1	-0.090956466	0.282067705	0.427201992
RNF5	0.249909325	0.167614943	0.224458263
RPN1	-0.00840644	0.912798848	1
RPN2	-0.010289403	0.90753017	0.999456943
RRBP1	-0.044216132	0.590373232	0.84305055
SAR1A	-0.009191662	0.903865087	0.997893009
SAR1B	-0.012020133	0.850266483	0.993017965

SEC13	0.038741149	0.575308486	0.828576978
SEC23A	-0.008835874	0.926486277	1
SEC23B	-0.019503886	0.824832001	0.993017965
SEC24A	-0.02802575	0.809661788	0.993017965
SEC24B	-0.109016005	0.181150499	0.248166155
SEC24C	-0.02074248	0.79923445	0.989170476
SEC24D	0.06699759	0.428207736	0.642909201
SEC31A	-0.048936166	0.467517817	0.697359546
SEC31B	-0.015252849	0.899591629	0.99619613
SEC61A1	0.015760673	0.804192011	0.992852481
SEC61A2	-0.126420437	0.182351406	0.250856876
SEC61B	0.048406191	0.725665345	0.944183473
SEC61G	0.033188795	0.765804128	0.964873315
SEC62	0.066993131	0.425442981	0.638631164
SEC63	0.017028265	0.819170253	0.993017965
SEL1L	-0.006841532	0.928561892	1
SIL1	0.120190262	0.317505822	0.482601775
SKP1	-0.021639638	0.76726016	0.965886524
SSR1	0.061203562	0.466219741	0.696051077
SSR2	0.02625855	0.737994841	0.95228687
SSR3	0.032178011	0.654125701	0.895577112
SSR4	0.012745906	0.898038942	0.995811867
STT3A	0.036998633	0.658397793	0.897511674
STT3B	0.025926771	0.773037108	0.969430453
STUB1	0.005928304	0.941118859	1
SVIP	0.066199387	0.457313668	0.685165325
SYVN1	0.039574585	0.55950895	0.809143736
TRAF2	-0.016765601	0.881468537	0.993017965
TRAM1	0.032896168	0.728785757	0.946248622
TUSC3	-0.00929134	0.914057929	1
TXNDC5	0.083035209	0.523342805	0.766355471
UBE2D1	-0.125335946	0.276006512	0.417563888
UBE2D2	0.033436207	0.676764495	0.914911246
UBE2D3	0.026529833	0.740844246	0.95228687
UBE2D4	-0.010900293	0.912931061	1
UBE2E1	0.010547037	0.894623851	0.994390931
UBE2E2	0.101797409	0.432129742	0.647776291
UBE2E3	-0.020118927	0.766551781	0.965492779
UBE2G1	0.005878768	0.959889107	1
UBE2G2	-0.021487958	0.79078306	0.982556181
UBE2J1	-0.005258362	0.968385928	1

UBE2J2	-0.119709682	0.221219606	0.316420574
UBE4B	-0.024646192	0.782048181	0.976711933
UBQLN1	0.051591776	0.341759559	0.517282868
UBQLN2	0.046786148	0.469756261	0.69988123
UBQLN4	0.009201442	0.917088157	1
UBQLNL	0.071783173	0.549416529	0.795525837
UBXN6	0.111697779	0.249533853	0.371629671
UFD1L	-0.005880474	0.919213328	1
UGGT1	0.002325935	0.979651056	1
UGGT2	-0.013674879	0.869005723	0.993017965
VCP	0.020456793	0.699357297	0.925301986
WFS1	0.003500233	0.961781482	1
XBP1	0.023941113	0.815399529	0.993017965
YOD1	0.016727595	0.888340058	0.993017965

Supplementary Table 5. Significantly dysregulated (q<0.05) pathways comparing C57BL/6J (Nnt mutant) to C57BL/6N (Nnt wild-type) mice (5A) and C57BL/6J (NNT mutant) to C57BL/6J^{BAC} (transgenic) mice (5B). *Pathways that are shared between the two comparisons are highlighted in bold.*

Supplementary Table 5A

**C57BL/6J (*Nnt* mutant) vs C57BL/6N (*Nnt* wild-type)**		
Significantly dysregulated pathways (bi-directional changes)	**p value**	**q value**
**mmu00190 Oxidative phosphorylation**	1.58048E-06	0.000308669
mmu01200 Carbon metabolism	3.01141E-06	0.000308669
mmu00020 Citrate cycle (TCA cycle)	1.28873E-05	0.00088063
**mmu00640 Propanoate metabolism**	3.83142E-05	0.001963602
mmu01212 Fatty acid metabolism	7.77069E-05	0.003185983
**mmu04723 Retrograde endocannabinoid signaling**	0.000140909	0.003922293
mmu00620 Pyruvate metabolism	0.000146728	0.003922293
mmu03320 PPAR signaling pathway	0.000153065	0.003922293
mmu04151 PI3K-Akt signaling pathway	0.001317175	0.030002327
mmu04260 Cardiac muscle contraction	0.001849233	0.035754338
mmu00500 Starch and sucrose metabolism	0.002008868	0.035754338
mmu04512 ECM-receptor interaction	0.002293544	0.035754338

mmu00280 Valine, leucine and isoleucine degradation	0.002298437	0.035754338
mmu04020 Calcium signaling pathway	0.002550279	0.035754338
**mmu04713 Circadian entrainment**	0.002786827	0.035754338
mmu04612 Antigen processing and presentation	0.002790582	0.035754338
**mmu04010 MAPK signaling pathway**	0.003163588	0.038149144
Significantly up-regulated pathways	**p value**	**q value**
**mmu00190 Oxidative phosphorylation**	2.06663E-12	4.23659E-10
mmu01200 Carbon metabolism	4.8563E-08	4.97771E-06
mmu00640 Propanoate metabolism	7.01511E-07	4.79366E-05
mmu04151 PI3K-Akt signaling pathway	1.53587E-06	7.87135E-05
mmu00020 Citrate cycle (TCA cycle)	4.88167E-06	0.000200148
mmu04510 Focal adhesion	6.53269E-06	0.000223017
mmu04512 ECM-receptor interaction	7.61521E-06	0.000223017
mmu04723 **Retrograde endocannabinoid signaling**	8.82393E-06	0.000226113
**mmu04024 cAMP signaling pathway**	3.37987E-05	0.000769859
mmu00280 Valine, leucine and isoleucine degradation	4.07869E-05	0.000796474
mmu04260 Cardiac muscle contraction	4.27376E-05	0.000796474
mmu00620 Pyruvate metabolism	0.000160913	0.002748933
**mmu04010 MAPK signaling pathway**	0.000174536	0.002752294
mmu03320 PPAR signaling pathway	0.00021487	0.003146314
**mmu04910 Insulin signaling pathway**	0.000256595	0.003506801
mmu04152 AMPK signaling pathway	0.000383166	0.004845375
mmu04066 HIF-1 signaling pathway	0.000401812	0.004845375
**mmu04915 Estrogen signaling pathway**	0.000548811	0.006250352
mmu04974 Protein digestion and absorption	0.000683958	0.007379542
mmu04540 Gap junction	0.000987373	0.009854066
mmu00650 Butanoate metabolism	0.001009441	0.009854066
mmu00900 Terpenoid backbone biosynthesis	0.001787018	0.016651756
mmu04920 Adipocytokine signaling pathway	0.00230064	0.019307492
mmu04146 Peroxisome	0.002322073	0.019307492
mmu04068 FoxO signaling pathway	0.002354572	0.019307492
mmu04922 Glucagon signaling pathway	0.002649164	0.020887641
mmu04022 cGMP-PKG signaling pathway	0.003722971	0.028267001
mmu00010 Glycolysis / Gluconeogenesis	0.004342057	0.031790058
mmu00500 Starch and sucrose metabolism	0.004680431	0.033075304
mmu01212 Fatty acid metabolism	0.004840288	0.033075304
mmu01230 Biosynthesis of amino acids	0.006365407	0.040823184
mmu01210 2-Oxocarboxylic acid metabolism	0.006372399	0.040823184
mmu00030 Pentose phosphate pathway	0.007110632	0.043781916
**mmu04727 GABAergic synapse**	0.007261391	0.043781916
mmu04918 Thyroid hormone synthesis	0.007932356	0.04646094
mmu04970 Salivary secretion	0.008794037	0.04854583
mmu00062 Fatty acid elongation	0.008808562	0.04854583
mmu04530 Tight junction	0.008998739	0.04854583

Supplementary Table 5B

**C57BL/6J (*Nnt* mutant) vs C57BL/6J-BAC (*Nnt* transgenic)**		
significantly dysregulated pathways (bi-directional changes)	**p value**	**q value**
mmu04514 Cell adhesion molecules (CAMs)	0.000252642	0.051791633
Significantly up- regulated pathways	**p value**	**q value**
mmu04742 Taste transduction	2.57846E-05	0.005285834
**mmu04724 Glutamatergic synapse**	5.15961E-05	0.005288602
**mmu04723 Retrograde endocannabinoid signaling**	9.33199E-05	0.006376862
**mmu04713 Circadian entrainment**	0.000227984	0.011255597
**mmu04727 GABAergic synapse**	0.000274527	0.011255597
**mmu04024 cAMP signaling pathway**	0.000447334	0.01392701
**mmu00190 Oxidative phosphorylation**	0.000475556	0.01392701
mmu04012 ErbB signaling pathway	0.000875639	0.022438257
**mmu04910 Insulin signaling pathway**	0.001089304	0.022497227
**mmu04010 MAPK signaling pathway**	0.001147763	0.022497227
**mmu04725 Cholinergic synapse**	0.001207168	0.022497227
**mmu04921 Oxytocin signaling pathway**	0.002491578	0.041579918
mmu04070 Phosphatidylinositol signaling system	0.002694835	0.041579918
**mmu04915 Estrogen signaling pathway**	0.002933384	0.041579918
**mmu00640 Propanoate metabolism**	0.003042433	0.041579918

Supplementary Table 6. Intracellular and extracellular metabolome analysis comparing KD siRNA to SCR siRNA cells (A, B) and KD shRNA to SCR shRNA cells (C, D). *Metabolites with q<0.01 and <0.8 or >1.2- fold change are tabulated for the KD siRNA to SCR siRNA comparison. Metabolites with p<0.05 and <0.8 or >1.2-fold change are tabulated for the KD shRNA to SCR shRNA comparison (no metabolites with q<0.05 for this comparison).*

Supplementary Table 6A

**Significantly altered (q<0.01) intracellular metabolites (KD siRNA vs SCR siRNA)**	**p value**	**q value**	**Fold change (KD siRNA to SCR siRNA)**	**Metabolite class**
N-stearoyl histidine	0.002	0.006	0.36	Acyl amino acid
N-arachidonoyl GABA;;N-oleoyl GABA	0.002	0.006	0.42	Acyl amino acid
N-Acetylaspartylglutamic acid	0.002	0.006	0.57	Acyl amino acid
N5-Hexanoylspermidine	0.004	0.007	0.75	Acyl amino acid
Hexanoylglycine;Isovalerylalanine;Isovalerylsarcosine	0.002	0.007	1.36	Acyl amino acid
2-Methylbutyrylglycine;Dehydrocarnitine;Isovalerylglycine;N-Acetylvaline;Valerylglycine	0.002	0.006	1.49	Acyl amino acid
N1,N12-Diacetylspermine	0.002	0.007	1.54	Acyl amino acid
N-Acetyl-L-aspartic acid;N-Formyl-L-glutamic acid	0.002	0.007	2.13	Acyl amino acid
Glutarylglycine;N-Acetylglutamic acid	0.004	0.007	3.38	Acyl amino acid
3-Phenylpropionylglycine;N-Acetyl-phenylalanine;Phenylpropionylglycine	0.002	0.006	4.66	Acyl amino acid
N-Acetylputrescine	0.002	0.007	7.46	Acyl amino acid
Pentadecanoylglycine	0.002	0.007	9.37	Acyl amino acid
N-arachidonoyl glutamic acid	0.004	0.007	14.19	Acyl amino acid
N-Acetylcadaverine	0.004	0.007	43.76	Acyl amino acid
N1,N8-Diacetylspermidine	0.002	0.007	46.67	Acyl amino acid
Propenoylcarnitine	0.004	0.007	0.01	Acyl carnitine
Hexadecenoylcarnitine	0.002	0.006	0.02	Acyl carnitine

Malonylcarnitine	0.004	0.007	0.02	Acyl carnitine
Butyrylcarnitine;Isobutyryl-carnitine	0.002	0.006	1.54	Acyl carnitine
Clupanodonyl carnitine	0.002	0.006	1.70	Acyl carnitine
Hydroxyhexadecanoylcarnitine	0.002	0.006	1.77	Acyl carnitine
Propionylcarnitine	0.004	0.007	1.80	Acyl carnitine
Dehydroxycarnitine	0.002	0.006	1.83	Acyl carnitine
3-Methylglutarylcarnitine	0.004	0.007	2.07	Acyl carnitine
Cervonyl carnitine	0.002	0.006	2.45	Acyl carnitine
Hydroxy-octadecanoylcarnitine	0.002	0.006	2.54	Acyl carnitine
3-Hydroxy-octadecenoylcarnitine	0.002	0.007	3.85	Acyl carnitine
Butenylcarnitine	0.002	0.007	5.46	Acyl carnitine
Hydroxy-hexadecenoylcarnitine	0.002	0.006	12.36	Acyl carnitine
Heptanoylcarnitine	0.004	0.007	30.87	Acyl carnitine
2-trans,4-cis-Decadienoylcarnitine	0.002	0.006	40.24	Acyl carnitine
9-Decenoylcholine	0.002	0.007	0.03	Acyl choline
Butyrylcholine	0.002	0.007	1.64	Acyl choline
Caproylcholine	0.002	0.007	1.88	Acyl choline
TG[57:6];;TG[50:1]	0.004	0.007	1.60	Acyl glyceride
TG[51:3]	0.004	0.007	1.69	Acyl glyceride
DG[31:0]	0.002	0.006	6.32	Acyl glyceride
TG[44:1]	0.004	0.007	8.86	Acyl glyceride
DG[38:0]	0.004	0.007	340.89	Acyl glyceride
Proline	0.002	0.007	0.56	Arginine and proline metabolism
2-Oxoarginine	0.004	0.009	0.77	Arginine and proline metabolism
Creatinine	0.004	0.007	1.24	Arginine and proline metabolism
Dehydrospermidine	0.004	0.007	1.37	Arginine and proline metabolism
Creatine	0.002	0.006	2.10	Arginine and proline metabolism
Arginine	0.002	0.006	5.31	Arginine and proline metabolism

Glutamic acid 5-phosphate	0.002	0.007	12.60	Arginine and proline metabolism
Phosphoarginine	0.004	0.009	19.86	Arginine and proline metabolism
Asymmetric dimethylarginine;Symmetric dimethylarginine	0.002	0.007	24.04	Arginine and proline metabolism
Spermine	0.002	0.006	0.13	Arginine and proline metabolism and glutathione metabolism
Spermidine	0.002	0.006	0.39	Arginine and proline metabolism and glutathione metabolism
Spermine dialdehyde	0.004	0.007	1.78	Arginine and proline metabolism and glutathione metabolism
2-Hydroxyphenethylamine;Dimethylaniline-N-oxide;Tyramine	0.004	0.007	0.00	Aromatic metabolites and associated metabolism
Phenylethylamine;N,N-Dimethylaniline";Phenylethylamine	0.004	0.007	0.13	Aromatic metabolites and associated metabolism
Methylhippuric acid;Methyl n-acetylanthranilate	0.004	0.009	0.42	Aromatic metabolites and associated metabolism
Hydroxycinnamic acid;Coumaric acid;Enol-phenylpyruvate;Phenylpyruvic acid	0.004	0.009	0.56	Aromatic metabolites and associated metabolism
N'-Formylkynurenine	0.004	0.007	0.60	Aromatic metabolites and associated metabolism
Formyl-5-hydroxykynurenamine;L-Kynurenine;;5-Hydroxyindoleacetic acid	0.004	0.009	0.67	Aromatic metabolites and associated metabolism
5-Hydroxy-L-tryptophan	0.002	0.006	0.79	Aromatic metabolites and associated

				metabolism
3-Methoxytyramine;4-(beta-Methylaminoethyl)catechol;4-Methoxytyramine	0.004	0.007	1.22	Aromatic metabolites and associated metabolism
DOPA sulfate	0.002	0.007	1.50	Aromatic metabolites and associated metabolism
Tyrosine	0.002	0.007	1.57	Aromatic metabolites and associated metabolism
3-Hydroxymandelic acid;"3,4-Dihydroxybenzeneacetic acid";"3,4-Dihydroxymandelaldehyde";Homogentisic acid;Orsellinic acid;p-Hydroxymandelic acid	0.004	0.007	1.60	Aromatic metabolites and associated metabolism
4-(2-Amino-3-hydroxyphenyl)-2,4-dioxobutanoic acid";4-Aminohippuric acid	0.002	0.007	1.64	Aromatic metabolites and associated metabolism
Indole	0.004	0.007	1.91	Aromatic metabolites and associated metabolism
Cinnamyl benzoate	0.002	0.006	2.14	Aromatic metabolites and associated metabolism
Quinaldic acid	0.004	0.007	2.17	Aromatic metabolites and associated metabolism
Tryptophan	0.004	0.007	2.22	Aromatic metabolites and associated metabolism
2-(Methylamino)benzoic acid;2-Phenylglycine	0.004	0.007	2.43	Aromatic metabolites and associated metabolism
5-Hydroxyindoleacetaldehyde	0.004	0.007	2.68	Aromatic metabolites and associated metabolism

Phenylbutyric acid;Benzenebutanoic acid	0.002	0.007	5.93	Aromatic metabolites and associated metabolism
5-Methoxytryptophol	0.002	0.006	21.57	Aromatic metabolites and associated metabolism
S-(2-Methylpropionyl)-dihydrolipoamide-E	0.002	0.006	0.30	BCAA metabolism
S-Acetyldihydrolipoamide	0.002	0.006	1.29	BCAA metabolism
3-Isopropylmalate;3-propylmalic acid	0.004	0.007	2.16	BCAA metabolism
Isopropylmaleate	0.004	0.007	2.54	BCAA metabolism
Beta-Leucine;Leucine;isoleucine;Norleucine	0.002	0.006	23.84	BCAA metabolism
5-Acetylamino-6-formylamino-3-methyluracil	0.002	0.006	0.02	Caffeine metabolism
4-O-Methyl-myo-inositol	0.004	0.009	0.00	Carbohydrate
Allose;Glucose;Galactose;Fructose;Mannose;Tagatose;Sorbose;Myoinositol	0.004	0.009	0.01	Carbohydrate
Galactitol;L-Iditol;Mannitol;Sorbitol	0.004	0.007	0.41	Carbohydrate
Myo-inositol-1,3-bisphosphate;Myo-inositol-1,4-bisphosphate";Myo-inositol-3,4-bisphosphate";Glucose-1,6-bisphosphate";Fructose-2,6-bisphosphate'Fructose 1,6-bisphosphate	0.002	0.007	0.43	Carbohydrate
Arabinonic acid;Ribonic acid;Dihydroxybutanoic acid;Deoxyerythronic acid;Deoxythreonic acid	0.004	0.009	0.47	Carbohydrate
N-Acetylneuraminate 9-phosphate;N-Acetylneuraminic acid 9-phosphate	0.002	0.006	0.50	Carbohydrate
Ribose 1,5-bisphosphate	0.004	0.009	0.56	Carbohydrate
UDP-N-acetyl-D-mannosamine;Uridine diphosphate-N-acetylgalactosamine;Uridine diphosphate-N-acetylglucosamine	0.002	0.006	0.63	Carbohydrate
Deoxyribonic acid;Arabinose;Apiose;Ribose;Ribulose;Xylose;Xylulose	0.004	0.009	0.72	Carbohydrate
Glucose;Galactose;Fructose;Mannose;Tagatose;Sorbose;Myoinositol	0.002	0.006	1.57	Carbohydrate
Erythronic acid;Threonic acid	0.002	0.007	1.83	Carbohydrate

Galactonic acid;Gluconic acid;Gulonic acid	0.004	0.009	1.85	Carbohydrate
N-Acetyl-galactosamine;N-Acetyl-glucosamine	0.004	0.009	1.91	Carbohydrate
Glucosamine;Fructosamine	0.004	0.007	2.21	Carbohydrate
Neuraminic acid;N-Acetylglucosamine;N-Acetyl-galactosamine	0.004	0.009	5.77	Carbohydrate
Glucaric acid;Galactaric acid	0.002	0.007	15.42	Carbohydrate
Lactose;Maltose;Epimelibiose;Galabiose;Galactinol;Isomaltose;Lactulose;Mannobiose;Neotrehalose;Trehalose;Turanose	0.002	0.006	231.41	Carbohydrate
N-Acetyl-9-O-lactoylneuraminic acid	0.004	0.009	607.85	Carbohydrate
6-(alpha-D-Glucosaminyl)-1D-myo-inositol;Lactosamine	0.004	0.007	1670.40	Carbohydrate
SM(d18:2/24:1);;SM(d18:0/22:0)	0.004	0.007	0.56	Ceramide and sphingolipid
PE-Cer(d14:2/23:0);PE-Cer(d15:2/22:0);PE-Cer(d16:2/21:0);SM(d16:1/18:1);SM(d18:1/16:1);SM(d18:2/16:0)	0.004	0.007	1.47	Ceramide and sphingolipid
PE-Cer(d14:1/21:0);PE-Cer(d15:1/20:0);PE-Cer(d16:1/19:0);SM(d16:1/16:0);SM(d18:1/14:0)	0.004	0.007	1.52	Ceramide and sphingolipid
Safingol (L-threo-sphinganine);Sphinganine	0.004	0.007	1.74	Ceramide and sphingolipid
PI-Cer(d18:0/18:0);PI-Cer(d20:0/16:0)	0.004	0.007	1.89	Ceramide and sphingolipid
PE-Cer(d14:1/22:1(2OH));PE-Cer(d14:2/22:0(2OH));PE-Cer(d16:1/20:1(2OH));PE-Cer(d16:2/20:0(2OH))	0.004	0.007	1.94	Ceramide and sphingolipid
PE-Cer(d14:2/25:0);PE-Cer(d15:2/24:0);PE-Cer(d16:2/23:0);SM(d16:1/20:1);SM(d18:0/18:2);SM(d18:1/18:1);SM(d18:2/18:0);SM(d19:1/17:1)	0.004	0.007	1.98	Ceramide and sphingolipid
3-Dehydrosphinganine;"5-hydroxy,-sphingosine";d18:1) sphingosine;Sphingosine	0.002	0.006	2.12	Ceramide and sphingolipid
SM(d18:1/22:1);SM(d18:2/22:0)	0.004	0.007	2.63	Ceramide and sphingolipid
SM(d16:1/22:1);SM(d18:1/20:1);SM(d18:2/20:0)	0.004	0.007	3.68	Ceramide and sphingolipid

PE-Cer(d14:2/24:1(2OH));PE-Cer(d16:2/22:1(2OH))	0.004	0.009	20.14	Ceramide and sphingolipid
(4-Hydroxybenzoyl)choline	0.004	0.009	2.48	Choline metabolism
NADH	0.002	0.007	0.73	Co-factor
FAD	0.002	0.007	1.28	Co-factor
Thiosulfate	0.004	0.009	0.64	Cysteine and methionine metabolism
Aspartic acid	0.002	0.007	0.71	Cysteine and methionine metabolism
bromo-octadecadieneynoic acid";"Methyl-bromo-octadecadieneynoate	0.002	0.007	0.03	Fatty acid
hexadecatrienoic acid	0.002	0.006	0.10	Fatty acid
methyl-hexatriacontahexaenoic acid	0.004	0.007	0.12	Fatty acid
Hexacosanedioic acid	0.002	0.006	0.12	Fatty acid
Tetradecanedioic acid	0.002	0.007	0.19	Fatty acid
Octadecenoic acid;methyl-heptadecenoic acid	0.002	0.006	0.25	Fatty acid
Octadecatrienynoic acid	0.002	0.007	0.30	Fatty acid
Petroselinic acid	0.002	0.006	0.37	Fatty acid
Arachidonic Acid (d8)	0.002	0.006	0.50	Fatty acid
triacontatetraenoic acid	0.002	0.006	0.60	Fatty acid
Tetradecadienoic acid	0.002	0.006	0.61	Fatty acid
Tridecatrienediynol;Decenoic acid	0.004	0.009	0.61	Fatty acid
Dimethyl-2,4-octadienedioic acid	0.004	0.009	0.68	Fatty acid
Dimethyl-heptadienoic acid	0.002	0.006	1.38	Fatty acid
octatriacontatetraenoic acid	0.004	0.007	1.44	Fatty acid
2-Aminooctanoic acid;Octenoic acid	0.004	0.007	1.45	Fatty acid
Arachidonoyl-EA(d8)	0.002	0.006	1.53	Fatty acid
Decenedioic acid	0.002	0.006	1.61	Fatty acid
Undecanedicarboxylic acid";dodecenoic acid	0.004	0.007	2.00	Fatty acid
Octatriynoic acid	0.004	0.007	3.18	Fatty acid
Octadecenetriynoic acid	0.004	0.007	3.57	Fatty acid

Docosahexaenoic acid	0.002	0.006	8.12	Fatty acid
Hexadecenoic acid;methyl-pentadecenoic acid	0.002	0.006	9.00	Fatty acid
Eicosenoic acid;Phytenic acid;Phytenoic acid	0.002	0.006	16.96	Fatty acid
9,10-dibromo-stearic acid	0.002	0.007	586.37	Fatty acid
Nonadecanetriol;;Octadecanol	0.002	0.006	0.10	Fatty alcohol
Pentanol;Methyl-1-butanol;Isopentanol	0.002	0.006	1.25	Fatty alcohol
Pentenol	0.002	0.006	1.37	Fatty alcohol
Decadienol	0.004	0.007	1.89	Fatty alcohol
(-)Nhydroxy-propyl) alpha,alpha-dimethylarachidonoyl amine;"(-)Nmethyl-2-hydroxy-ethyl) alpha,alpha-dimethylarachidonoyl amine";"(+)Nhydroxy-propyl) alpha,alpha-dimethylarachidonoyl amine";"(+)Nmethyl-2-hydroxy-ethyl) alpha,alpha-dimethylarachidonoyl amine";N-(5-hydroxy-pentyl) arachidonoyl amine;N-propyl N-(2-hydroxy-ethyl) arachidonoyl amine	0.004	0.007	0.51	Fatty amine or amide
Octadecylamine	0.002	0.006	206.38	Fatty amine or amide
Arachidyl myristate;Behenyl laurate;Lauryl behenate;Myristyl arachidate;Palmityl stearate;Stearyl palmitate	0.004	0.007	1.79	Fatty ester
Arachidyl linoleate;Linoleyl arachidate	0.004	0.007	2.02	Fatty ester
Linoleyl stearate;Oleyl oleate;Stearyl linoleate	0.004	0.007	2.11	Fatty ester
Hydantoin-5-propionic acid	0.002	0.007	0.42	Folate metabolism
Hydroxysepiapterin;Threoneopterin;Neopterin	0.002	0.006	0.75	Folate metabolism
Dihydroneopterin phosphate;;Threoneopterin;Neopterin	0.004	0.009	0.78	Folate metabolism
Hydroxytetrahydrobiopterin;Tetrahydroneopterin	0.004	0.009	1.46	Folate metabolism
4a-Hydroxytetrahydrobiopterin;Tetrahydroneopterin	0.002	0.007	1.95	Folate metabolism
Sapropterin;Tetrahydrobiopterin	0.004	0.009	4.26	Folate metabolism
Glutamine	0.002	0.007	0.75	Glutamine and

				glutamate metabolism
4-Hydroxyglutamate semialdehyde;Glutamic acid	0.002	0.007	5.04	Glutamine and glutamate metabolism
Oxidized glutathione	0.004	0.009	1.59	Glutathione metabolism
Glutathione	0.002	0.007	1.65	Glutathione metabolism
Triethanolamine	0.002	0.006	0.00	Glycerophospholipid metabolism
PC[37:4];PE[40:4];;PC[35:1];PE[38:1];;PA[42:5];PC(O-16:0/O-16:0)	0.004	0.007	0.00	Glycerophospholipid metabolism
PC[31:4];PE[34:4];;PC[29:1];PE[32:1];;PA[36:5]	0.004	0.009	0.03	Glycerophospholipid metabolism
PI[34:3];;PI[32:0]	0.004	0.009	0.13	Glycerophospholipid metabolism
PE[32:3];;PC[27:0];PE-NMe2[28:0];PE[30:0];;PA[34:4]	0.004	0.007	0.14	Glycerophospholipid metabolism
LysoPE[20:4];;LysoPE[18:1]	0.004	0.007	0.15	Glycerophospholipid metabolism
PC(O-20:0/O-1:0)	0.002	0.006	0.17	Glycerophospholipid metabolism
LysoPE[22:4];;LysoPE[20:1]	0.004	0.007	0.26	Glycerophospholipid metabolism
Glycerophosphocholine	0.004	0.007	0.31	Glycerophospholipid metabolism
PC[30:3];PE[33:3];;PC[28:0];PE[31:0];;PA[35:4]	0.004	0.007	0.31	Glycerophospholipid metabolism
PE[30:3];;PC[25:0];PE[28:0]	0.002	0.006	0.32	Glycerophospholipid metabolism
PG[35:6];;PG[33:3];PG[31:0];;PA[35:3];;PA[33:0]	0.004	0.007	0.32	Glycerophospholipid metabolism
PC[44:8];;PC[42:5];;PS(O-20:0/21:0);;PC[40:2];PE[43:2];PC(20:0/P-18:0);PC(22:0/P-16:0);PC(O-16:0/22:1);PC(o-16:1/22:0);PC(O-18:0/20:1);PC(o-18:1/20:0);PC(O-20:0/18:1);PC(P-16:0/22:0);PC(P-18:0/20:0);PC(P-20:0/18:0);PE(P-20:0/21:0)	0.004	0.007	0.33	Glycerophospholipid metabolism
PG(P-18:0/22:6);;PG[37:1];;PG(O-16:0/22:4);PG(O-18:0/20:4);PG(O-	0.004	0.009	0.33	Glycerophospholipi

20:0/18:4);PG(P-18:0/20:3);PG(P-20:0/18:3);;PA(P-20:0/22:6);;PA[39:1];;PA(O-18:0/22:4);PA(O-20:0/20:4);PA(P-20:0/20:3)				d metabolism
PC[39:6];PE[42:6];;PC[37:3];PE[40:3];;PA[44:7]	0.004	0.009	0.34	Glycerophospholipid metabolism
PC[20:4]	0.002	0.007	0.36	Glycerophospholipid metabolism
LysoPC(O-18:0)	0.002	0.006	0.37	Glycerophospholipid metabolism
PI[36:2]	0.004	0.009	0.43	Glycerophospholipid metabolism
PS[37:1];PT[36:1];;PC[34:2];PE-NMe[36:2];PE[37:2]	0.004	0.009	0.44	Glycerophospholipid metabolism
PC[32:4];PE[35:4];;PC[30:1];PE[33:1];;PA[37:5]	0.004	0.007	0.44	Glycerophospholipid metabolism
PS[35:2];;PG[35:4];;PC[30:0];PE-NMe[32:0];PE[33:0]	0.004	0.009	0.45	Glycerophospholipid metabolism
PS[36:2];;PG[36:4];;PC[31:0];PE-NMe2[32:0];PE[34:0];PC[33:3];PE[36:3]	0.004	0.007	0.45	Glycerophospholipid metabolism
PC[36:6];PE[39:6];;PC[34:3];PE[37:3];;PA[41:7];;PC[32:0];PE(18:0(10(R)Me)/16:0);PE[35:0]	0.004	0.007	0.45	Glycerophospholipid metabolism
PC[34:5];PE[37:5];;PC[32:2];PE[35:2];;PA[39:6]	0.004	0.007	0.46	Glycerophospholipid metabolism
PC[32:3];PE[35:3];;PC[30:0];PE-NMe[32:0];PE[33:0];;PA[37:4]	0.004	0.007	0.47	Glycerophospholipid metabolism
PS[36:2];;PG[36:4];;PC[31:0];PE-NMe2[32:0];PE[34:0];PC[33:3];PE[36:3]	0.004	0.009	0.48	Glycerophospholipid metabolism
PC[39:6];PE[42:6];;PC[37:3];PE[40:3];;PA[44:7];;PC[35:0];PE-NMe2[36:0];PE[38:0]	0.004	0.007	0.50	Glycerophospholipid metabolism
1-(sn-Glycero-3-phospho)-1D-myo-inositol	0.004	0.009	0.51	Glycerophospholipid metabolism
PG[39:3];;PG(O-18:0/22:6);PG(P-20:0/20:5);;PA[41:3];;PA(O-20:0/22:6)	0.004	0.009	0.54	Glycerophospholipid metabolism
PE(20:5/P-18:1);PE(22:6/P-16:0);PE(O-16:1/22:6);PE(P-18:1/20:5);;PE(18:2/P-18:1);PE(18:3/P-18:0);PE(20:3/P-16:0);PE(O-16:0/20:4);PE(O-18:0/18:4);PE(P-16:0/20:3);PE(P-18:0/18:3);PE(P-18:1/18:2);;PC(15:0/P-	0.004	0.007	0.57	Glycerophospholipid metabolism

16:0);PC(O-16:0/15:1);PC(P-16:0/15:0);PC(P-18:0/13:0);PE(16:0/P-18:0);PE(18:0/P-16:0);PE(O-18:0/16:1);PE(O-20:0/14:1);PE(P-16:0/18:0);PE(P-16:0e/18:1);PE(P-18:0/16:0);PE(P-20:0/14:0);;PE(O-16:0/O-16:0)				
PC[33:3];PE[36:3];;PC[31:0];PE-NMe2[32:0];PE[34:0];PA[38:4]	0.004	0.009	0.60	Glycerophospholipid metabolism
PS[37:2];;PG[37:4];PC[32:0];PE(18:0(10(R)Me)/16:0);PE[35:0]	0.004	0.009	0.60	Glycerophospholipid metabolism
PS[41:5];;PS[39:2];;PG[41:7];;PC[36:3];PE[39:3];;PC[34:0];PE-NMe[36:0];PE[37:0];;PC[38:6];PE[41:6]	0.004	0.009	0.61	Glycerophospholipid metabolism
PS[39:3];;PC[36:4];PE[39:4]	0.004	0.009	0.62	Glycerophospholipid metabolism
PS[39:3];;PS[37:0]	0.004	0.009	0.62	Glycerophospholipid metabolism
PC[33:2];PE[36:2];PA[38:3]	0.004	0.007	0.62	Glycerophospholipid metabolism
PC[34:2];PE-NMe[36:2];PE[37:2];;PA[39:3]	0.004	0.007	0.64	Glycerophospholipid metabolism
PC[42:8];;PC[40:5];;PS(O-18:0/21:0);PS(O-20:0/19:0);;PC[38:2];PE[41:2];PC(18:0/P-18:0);PC(20:0/P-16:0);PC(O-16:0/20:1);PC(o-16:1/20:0);PC(O-18:0/18:1);PC(o-18:1/18:0);PC(O-20:0/16:1);PC(P-16:0/20:0);PC(P-18:0/18:0);PC(P-20:0/16:0);PE(O-20:0/19:1);PE(P-18:0/21:0);PE(P-20:0/19:0)	0.004	0.007	0.66	Glycerophospholipid metabolism
PG(O-16:0/22:6);PG(P-18:0/20:5);;PG[35:0];;PG(O-16:0/20:3);PG(O-18:0/18:3);PG(P-16:0/20:2);PG(P-18:0/18:2);;PA(O-18:0/22:6);PA(P-20:0/20:5);;PA[37:0];;PA(O-18:0/20:3);PA(O-20:0/18:3);PA(P-16:0/22:2);PA(P-18:0/20:2);PA(P-20:0/18:2)	0.004	0.009	0.66	Glycerophospholipid metabolism
PC[38:7];PE[41:7];;PC[36:4];PE[39:4];;PC[34:1];PE[37:1];PC(16:0/O-16:0);PC(O-14:0/18:0);PC(O-16:0/16:0);PC(O-18:0/14:0);PC(O-20:0/12:0);PE(O-16:0/19:0);PE(O-18:0/17:0);PE(O-20:0/15:0)	0.004	0.007	0.67	Glycerophospholipid metabolism
PS[41:2];;PC[38:3];PE[41:3];;PC[36:0];PE[39:0];;PC[40:6];PE[43:6]	0.004	0.009	0.67	Glycerophospholipid metabolism

PC[32:0];PE(18:0(10(R)Me)/16:0);PE[35:0];;PA[37:1]	0.004	0.007	0.68	Glycerophospholipid metabolism
PC[44:7];;PC[42:4];;PC[40:1];PE[43:1];;GalCer(d18:0/24:0);GlcCer(d18:0/24:0);;PC(o-16:0/22:0);PC(o-18:0/20:0);PC(O-20:0/18:0);PE(O-20:0/21:0)	0.004	0.007	0.69	Glycerophospholipid metabolism
O-Phosphoethanolamine	0.004	0.009	0.69	Glycerophospholipid metabolism
PS[41:3];;PS[39:0];;PG[41:5];;PC[36:1];PE[39:1]	0.004	0.009	0.69	Glycerophospholipid metabolism
PC[39:7];PE[42:7];;PC[37:4];PE[40:4];;PA[44:8];;PC[35:1];PE[38:1];PC(O-16:0/17:0);PC(O-18:0/15:0);PC(O-20:0/13:0);PE(O-16:0/20:0);PE(O-18:0/18:0);PE(O-20:0/16:0)	0.004	0.007	0.69	Glycerophospholipid metabolism
PC[34:3];PE[37:3];;PC[32:0];PE(18:0(10(R)Me)/16:0);PE[35:0];;PA[39:4]	0.004	0.007	0.69	Glycerophospholipid metabolism
PS[37:3];;PS[35:0];;PG[37:5];;PC[32:1];PE-NMe[34:1];PE[35:1]	0.004	0.009	0.69	Glycerophospholipid metabolism
PC[34:4];PE[37:4];;PC[32:1];PE-NMe[34:1];PE[35:1];;PA[39:5]	0.004	0.007	0.71	Glycerophospholipid metabolism
PS[39:4];;PS[37:1];PT[36:1];;PG[39:6];;PC[34:2];PE-NMe[36:2];PE[37:2];;PC[36:5];PE[39:5]	0.004	0.009	0.71	Glycerophospholipid metabolism
PS[39:3];;PS[37:0];;PG[39:5];PC[34:1];PE[37:1]	0.004	0.009	0.73	Glycerophospholipid metabolism
PC[31:2];PE[34:2];;PA[36:3]	0.004	0.007	0.74	Glycerophospholipid metabolism
PE(16:1/P-18:1);PE(18:2/P-16:0);PE(O-16:0/18:3);PE(P-16:0/18:2);PE(P-18:1/16:1);;PC(O-14:0/15:0);PC(O-16:0/13:0);PE(O-16:0/16:0);PE(O-18:0/14:0);PE(O-20:0/12:0);;PA(O-16:0/20:4);PA(O-18:0/18:4);PA(P-16:0/20:3);PA(P-18:0/18:3)	0.004	0.007	0.74	Glycerophospholipid metabolism
PC[31:3];PE[34:3];PC(15:0/14:0);PC[29:0];PE[32:0];PA[36:4]	0.004	0.007	0.76	Glycerophospholipid metabolism
PC[37:6];PE(P-18:0/22:6(14OH));PE[40:6];;PC[35:3];PE[38:3];;PA[42:7]	0.004	0.007	0.77	Glycerophospholipid metabolism
PC[35:6];PE(P-16:0/22:6(14OH));PE[38:6];;PC[33:3];PE[36:3];;PA[40:7];;PC[31:0];PE-	0.004	0.007	0.78	Glycerophospholipid metabolism

NMe2[32:0];PE[34:0]				
PC[42:6];;PC[40:3];;PC[38:0];PE-NMe[40:0];PE[41:0]	0.004	0.007	0.78	Glycerophospholipid metabolism
PC[42:7];;PC[40:4];PE[43:4];;PC[38:1];PE[41:1];PC(O-14:0/22:0);PC(o-16:0/20:0);PC(o-18:0/18:0);PC(O-20:0/16:0);PE(O-18:0/21:0);PE(O-20:0/19:0)	0.004	0.007	0.80	Glycerophospholipid metabolism
PS[43:4];;PS[41:1];;PG[43:6];;PC[38:2];PE[41:2];;PC[40:5]	0.004	0.009	1.20	Glycerophospholipid metabolism
PC[35:5];PE(P-18:1/20:4(12OH[S]));PE(P-18:1/20:4(15OH[S]));PE(P-18:1/20:4(5OH[S]));PE[38:5];;PC[33:2];PE[36:2];;PA[40:6]	0.004	0.007	1.28	Glycerophospholipid metabolism
PC[36:2];PE[39:2];;PA[41:3]	0.004	0.007	1.31	Glycerophospholipid metabolism
PC(20:3/P-18:1);PC(20:4/P-18:0);PC(22:4/P-16:0);PC(O-16:0/22:5);PC(O-18:0/20:5);PC(o-18:1/20:4);PC(P-16:0/22:4);PC(P-18:0/20:4);PC(P-18:1/20:3);PC(P-20:0/18:4);;PC(18:0/P-18:1);PC(18:1/P-18:0);PC(20:1/P-16:0);PC(O-16:0/20:2);PC(o-18:0/18:2);PC(o-18:1/18:1);PC(P-16:0/20:1);PC(P-18:0/18:1);PC(P-18:1/18:0);PC(P-20:0/16:1);PE(P-20:0/19:1)	0.004	0.007	1.34	Glycerophospholipid metabolism
PC[38:5];PE[41:5];;PC[36:2];PE[39:2];;PA[43:6]	0.004	0.007	1.36	Glycerophospholipid metabolism
PC[38:3];PE[41:3];;PC[36:0];PE[39:0];;PA[43:4]	0.004	0.007	1.36	Glycerophospholipid metabolism
PC[42:5];;PC[40:2];PE[43:2]	0.004	0.007	1.40	Glycerophospholipid metabolism
PC[40:4];PE[43:4];;PC[38:1];PE[41:1]	0.004	0.007	1.40	Glycerophospholipid metabolism
PS[38:2]	0.004	0.007	1.40	Glycerophospholipid metabolism
PC[31:1];PE[34:1];PA[36:2];;PC(O-14:0/O-14:0)	0.004	0.007	1.40	Glycerophospholipid metabolism
PS[35:1];;PG[35:3]	0.004	0.009	1.42	Glycerophospholipid metabolism
PC(O-18:0/O-2:1);PC(P-20:0/0:0)	0.002	0.006	1.43	Glycerophospholipid metabolism

PS[22:2]	0.004	0.007	1.43	Glycerophospholipid metabolism
PS[36:4];;PG[36:6]	0.004	0.007	1.44	Glycerophospholipid metabolism
PE(20:3/P-18:1);PE(20:4/P-18:0);PE(22:4/P-16:0);PE(O-16:0/22:5);PE(O-18:0/20:5);PE(O-18:1/20:4);PE(P-16:0/22:4);PE(P-18:0/20:4);PE(P-18:1/20:3);PE(P-20:0/18:4);;PC(15:0/P-18:1);PC(O-16:0/17:2);PC(P-16:0/17:1);PC(P-18:0/15:1);PC(P-18:1/15:0);PE(18:0/P-18:1);PE(18:1/P-18:0);PE(20:1/P-16:0);PE(dm18:0/18:1);PE(O-16:0/20:2);PE(O-18:0/18:2);PE(P-16:0/20:1);PE(P-18:0/18:1);PE(P-18:1/18:0);PE(P-20:0/16:1);;PA(O-18:0/22:6);PA(P-20:0/20:5)	0.004	0.007	1.46	Glycerophospholipid metabolism
PS[38:3];;PS[36:0];;PG[38:5];PC[33:1];PE-NMe2[34:1];PE[36:1]	0.004	0.007	1.47	Glycerophospholipid metabolism
PC[34:1];PE[37:1];;PA[39:2];;PE-NMe2(O-16:0/O-16:0)	0.004	0.007	1.48	Glycerophospholipid metabolism
PE(18:2/P-18:1);PE(18:3/P-18:0);PE(20:3/P-16:0);PE(O-16:0/20:4);PE(O-18:0/18:4);PE(P-16:0/20:3);PE(P-18:0/18:3);PE(P-18:1/18:2);;PC(15:0/P-16:0);PC(O-16:0/15:1);PC(P-16:0/15:0);PC(P-18:0/13:0);PE(16:0/P-18:0);PE(18:0/P-16:0);PE(O-18:0/16:1);PE(O-20:0/14:1);PE(P-16:0/18:0);PE(P-16:0e/18:1);PE(P-18:0/16:0);PE(P-20:0/14:0);;PA(O-18:0/20:5);PA(P-16:0/22:4);PA(P-18:0/20:4);PA(P-20:0/18:4)	0.004	0.007	1.50	Glycerophospholipid metabolism
PS[38:4];;PS[36:1];;DGCC[36:5];;PG[38:6];;PC[33:2];PE[36:2]	0.004	0.007	1.50	Glycerophospholipid metabolism
PC[38:0];PE-NMe[40:0];PE[41:0]	0.004	0.007	1.51	Glycerophospholipid metabolism
PS[33:0];;PG[33:2];;PC[30:1];PE[33:1]	0.004	0.009	1.51	Glycerophospholipid metabolism
PC[32:2];PE[35:2];;PA[37:3]	0.004	0.007	1.52	Glycerophospholipid metabolism
PC[40:5];;PC[38:2];PE[41:2]	0.004	0.007	1.55	Glycerophospholipid metabolism
PC[29:2];PE[32:2];;PA[34:3]	0.004	0.007	1.55	Glycerophospholipi

				d metabolism
PS[41:1];;PG[41:3];;PC[38:2];PE[41:2]	0.004	0.009	1.57	Glycerophospholipid metabolism
PE(O-18:0/O-18:0);PE(O-20:0/O-16:0)	0.004	0.007	1.63	Glycerophospholipid metabolism
PA(P-20:0/22:2)	0.004	0.007	1.63	Glycerophospholipid metabolism
PI[38:3]	0.004	0.007	1.63	Glycerophospholipid metabolism
PS[35:0];PG[35:2]	0.004	0.009	1.64	Glycerophospholipid metabolism
PS[39:0];PG[39:2];;PC[36:1];PE[39:1]	0.004	0.009	1.67	Glycerophospholipid metabolism
PC(18:3/P-16:0);PC(O-14:0/20:4);PC(O-16:0/18:4);PC(P-16:0/18:3);;PC(14:0/P-18:0);PC(16:0/P-16:0);PC(O-14:0/18:1);PC(o-16:0/16:1);PC(O-18:0/14:1);PC(P-16:0/16:0);PC(P-18:0/14:0);PC(P-20:0/12:0);PE(O-16:0/19:1);PE(O-18:0/17:1);PE(O-20:0/15:1);PE(P-16:0/19:0);PE(P-18:0/17:0);PE(P-20:0/15:0)	0.004	0.007	1.67	Glycerophospholipid metabolism
LysoPC[16:0]	0.002	0.007	1.68	Glycerophospholipid metabolism
PC(O-15:0/20:4);PE(20:2/P-18:1);PE(20:3/P-18:0);PE(O-16:0/22:4);PE(O-18:0/20:4);PE(O-20:0/18:4);PE(P-18:0/20:3);PE(P-18:1/20:2);PE(P-20:0/18:3);;PC(15:0/P-18:0);PC(O-16:0/17:1);PC(O-18:0/15:1);PC(P-16:0/17:0);PC(P-18:0/15:0);PC(P-20:0/13:0);PE(18:0/P-18:0);PE(20:0/P-16:0);PE(dm18:0/18:0);PE(O-16:0/20:1);PE(O-18:0/18:1);PE(O-20:0/16:1);PE(P-16:0/20:0);PE(P-20:0/16:0);;PA(O-20:0/20:5);PA(P-18:0/22:4);PA(P-20:0/20:4)	0.004	0.009	1.71	Glycerophospholipid metabolism
PE(22:4/P-18:0);PE(O-20:0/20:5);PE(P-18:0/22:4);PE(P-20:0/20:4);;PC(O-18:0/17:2);PC(P-16:0/19:1);PC(P-18:0/17:1);PC(P-20:0/15:1);PE(20:0/P-18:1);PE(20:1/P-18:0);PE(22:1/P-16:0);PE(O-16:0/22:2);PE(O-18:0/20:2);PE(O-20:0/18:2);PE(P-16:0/22:1);PE(P-18:0/20:1);PE(P-	0.004	0.007	1.71	Glycerophospholipid metabolism

18:1/20:0);PE(P-20:0/18:1);;PA(O-20:0/22:6)				
PC[33:0];PE[36:0];;PA[38:1]	0.004	0.007	1.72	Glycerophospholipid metabolism
PS[34:2];;PG[34:4];PC(15:0/14:0);PC[29:0];PE[32:0]	0.004	0.007	1.79	Glycerophospholipid metabolism
PC[30:0];PE-NMe[32:0];PE[33:0]	0.004	0.009	1.79	Glycerophospholipid metabolism
PE(22:4/P-18:1);PE(22:5/dm18:0);PE(22:5/P-18:0);PE(O-18:0/22:6);PE(P-18:0/22:5);PE(P-18:1/22:4);PE(P-20:0/20:5);;PC(P-18:0/17:2);PE(20:1/P-18:1);PE(20:2/P-18:0);PE(22:2/P-16:0);PE(O-18:0/20:3);PE(O-20:0/18:3);PE(P-16:0/22:2);PE(P-18:0/20:2);PE(P-18:1/20:1);PE(P-20:0/18:2);;PA(P-20:0/22:6);;PC(O-16:0/17:0);PC(O-18:0/15:0);PC(O-20:0/13:0);PE(O-16:0/20:0);PE(O-18:0/18:0);PE(O-20:0/16:0)	0.004	0.007	1.81	Glycerophospholipid metabolism
PG[34:5]	0.004	0.007	1.83	Glycerophospholipid metabolism
PC[28:1];PE[31:1];PA[33:2]	0.004	0.007	1.85	Glycerophospholipid metabolism
PC[28:0];PE[31:0];;PA[33:1]	0.004	0.007	1.85	Glycerophospholipid metabolism
PC[30:2];PE[33:2];;PA[35:3]	0.004	0.007	1.88	Glycerophospholipid metabolism
PC[32:1];PE-NMe[34:1];PE[35:1];;PA[37:2];PE(O-16:0/O-16:0)	0.004	0.007	1.89	Glycerophospholipid metabolism
PC(15:0/P-18:0);PC(O-16:0/17:1);PC(O-18:0/15:1);PC(P-16:0/17:0);PC(P-18:0/15:0);PC(P-20:0/13:0);PE(18:0/P-18:0);PE(20:0/P-16:0);PE(dm18:0/18:0);PE(O-16:0/20:1);PE(O-18:0/18:1);PE(O-20:0/16:1);PE(P-16:0/20:0);PE(P-20:0/16:0)	0.004	0.007	1.92	Glycerophospholipid metabolism
PI[30:3]	0.004	0.007	1.96	Glycerophospholipid metabolism
LysoPC[14:0]	0.002	0.006	1.98	Glycerophospholipid metabolism
PC[38:2];PE[41:2]	0.004	0.007	2.01	Glycerophospholipi

				d metabolism
PC(P-16:0/17:2);PE(18:1/P-18:1);PE(18:2/P-18:0);PE(20:2/P-16:0);PE(O-16:0/20:3);PE(O-18:0/18:3);PE(P-16:0/20:2);PE(P-18:0/18:2);PE(P-18:1/18:1)	0.004	0.007	2.04	Glycerophospholipid metabolism
PC[30:1];PE[33:1];;PA[35:2];PE-NMe2(O-14:0/O-14:0)	0.004	0.007	2.05	Glycerophospholipid metabolism
PC(18:3/P-18:1);PC(18:4/dm18:0);PC(18:4/P-18:0);PC(20:4/P-16:0);PC(O-16:0/20:5);PC(o-16:1/20:4);PC(P-16:0/20:4);PC(P-18:0/18:4);PC(P-18:1/18:3);;PC(16:0/P-18:1);PC(16:1/P-18:0);PC(18:1/P-16:0);PC(O-16:0/18:2);PC(P-16:0/18:1);PC(P-18:0/16:1);PC(P-18:1/16:0);PC(P-20:0/14:1);PE(O-20:0/17:2);PE(P-18:0/19:1);PE(P-20:0/17:1);PnC[34:1]	0.004	0.007	2.10	Glycerophospholipid metabolism
PS(O-16:0/21:0);PS(O-18:0/19:0);PS(O-20:0/17:0);;PG(O-20:0/17:2);PG(P-18:0/19:1);PG(P-20:0/17:1);;PC(16:0/P-18:0);PC(18:0/P-16:0);PC(O-16:0/18:1);PC(o-16:1/18:0);PC(O-18:0/16:1);PC(o-18:1/16:0);PC(O-20:0/14:1);PC(P-16:0/18:0);PC(P-18:0/16:0);PC(P-20:0/14:0);PE(O-18:0/19:1);PE(O-20:0/17:1);PE(P-16:0/21:0);PE(P-18:0/19:0);PE(P-20:0/17:0)	0.004	0.009	2.15	Glycerophospholipid metabolism
PS[34:1];;PG[34:3]	0.004	0.007	2.16	Glycerophospholipid metabolism
PI[20:2]	0.004	0.007	2.18	Glycerophospholipid metabolism
PC[35:1];PE[38:1];;PA[40:2];;PC(O-16:0/O-16:0)	0.004	0.007	2.19	Glycerophospholipid metabolism
PC(O-20:0/22:6);;PC(22:1/P-18:1);PC(22:2/P-18:0);PC(O-20:0/20:3);PC(o-22:0/18:3);PC(P-18:0/22:2);PC(P-18:1/22:1);PC(P-20:0/20:2);;PC(o-16:0/22:0);PC(o-18:0/20:0);PC(O-20:0/18:0);PE(O-20:0/21:0)	0.004	0.007	2.20	Glycerophospholipid metabolism
PC[29:1];PE[32:1];;PA[34:2];;PE-NMe(O-14:0/O-14:0)	0.004	0.007	2.20	Glycerophospholipid metabolism
PC(16:0/P-18:1);PC(16:1/P-18:0);PC(18:1/P-16:0);PC(O-16:0/18:2);PC(P-16:0/18:1);PC(P-	0.004	0.007	2.20	Glycerophospholipid metabolism

18:0/16:1);PC(P-18:1/16:0);PC(P-20:0/14:1);PE(O-20:0/17:2);PE(P-18:0/19:1);PE(P-20:0/17:1);PnC[34:1]				
PC(20:4/P-18:1);PC(20:5/P-18:0);PC(22:5/P-16:0);PC(dm18:1/20:4);PC(o-16:0/22:6);PC(P-16:0/22:5);PC(P-18:0/20:5);PC(P-18:1/20:4);PC(18:1/P-18:1);PC(18:2/P-18:0);PC(20:2/P-16:0);PC(O-16:0/20:3);PC(O-18:0/18:3);PC(o-18:1/18:2);PC(P-16:0/20:2);PC(P-18:0/18:2);PC(P-18:1/18:1)	0.004	0.007	2.21	Glycerophospholipid metabolism
PC[36:1];PE[39:1];;PA[41:2];;PE(O-18:0/O-18:0);PE(O-20:0/O-16:0)	0.004	0.007	2.26	Glycerophospholipid metabolism
PI(O-16:0/12:0);;PG[30:0]	0.004	0.007	2.28	Glycerophospholipid metabolism
PS(P-20:0/19:1);PC(18:0/O-16:0);PC(o-16:0/18:0);PC(O-17:0/17:0);PC(O-18:0/16:0);PC(O-20:0/14:0);PE(O-16:0/21:0);PE(O-18:0/19:0);PE(O-20:0/17:0);;1-O-Hexadecyl-2-O-dihomogammalinolenoylglycero-3-phosphocholine;PC(18:1/P-18:1);PC(18:2/P-18:0);PC(20:2/P-16:0);PC(O-16:0/20:3);PC(O-18:0/18:3);PC(o-18:1/18:2);PC(P-16:0/20:2);PC(P-18:0/18:2);PC(P-18:1/18:1)	0.004	0.009	2.29	Glycerophospholipid metabolism
PS(O-20:0/19:1);PS(P-18:0/21:0);PS(P-20:0/19:0);PC(18:0/P-18:1);PC(18:1/P-18:0);PC(20:1/P-16:0);PC(O-16:0/20:2);PC(o-18:0/18:2);PC(o-18:1/18:1);PC(P-16:0/20:1);PC(P-18:0/18:1);PC(P-18:1/18:0);PC(P-20:0/16:1);PE(P-20:0/19:1);;PC(20:3/P-18:1);PC(20:4/P-18:0);PC(22:4/P-16:0);PC(O-16:0/22:5);PC(O-18:0/20:5);PC(o-18:1/20:4);PC(P-16:0/22:4);PC(P-18:0/20:4);PC(P-18:1/20:3);PC(P-20:0/18:4)	0.004	0.009	2.32	Glycerophospholipid metabolism
PC[40:2];PE[43:2]	0.004	0.007	2.38	Glycerophospholipid metabolism
LysoPE[18:1]	0.002	0.006	2.39	Glycerophospholipid metabolism
PS(O-16:0/21:0);PS(O-18:0/19:0);PS(O-20:0/17:0);PC(16:0/P-18:0);PC(18:0/P-16:0);PC(O-16:0/18:1);PC(o-16:1/18:0);PC(O-18:0/16:1);PC(o-18:1/16:0);PC(O-20:0/14:1);PC(P-	0.004	0.009	2.46	Glycerophospholipid metabolism

16:0/18:0);PC(P-18:0/16:0);PC(P-20:0/14:0);PE(O-18:0/19:1);PE(O-20:0/17:1);PE(P-16:0/21:0);PE(P-18:0/19:0);PE(P-20:0/17:0);;PC(18:2/P-18:1);PC(18:3/P-18:0);PC(20:3/P-16:0);PC(o-16:0/20:4);PC(O-18:0/18:4);PC(o-18:2/18:2);PC(P-16:0/20:3);PC(P-18:0/18:3);PC(P-18:1/18:2)				
LysoPC[18:1];PC(O-16:1/2:0);PC(P-16:0/2:0)	0.002	0.006	2.51	Glycerophospholipid metabolism
PG[17:1];LysoPC[14:0];PC(O-12:0/2:0)	0.002	0.007	2.58	Glycerophospholipid metabolism
PS[21:0];PC(O-16:1/2:0);PC(P-16:0/2:0)	0.002	0.007	2.64	Glycerophospholipid metabolism
PA(P-16:0/15:1)	0.004	0.007	2.70	Glycerophospholipid metabolism
PS[19:0];LysoPC[16:1];PC[16:1];PE[19:1]	0.002	0.007	2.75	Glycerophospholipid metabolism
PS[38:5];;PS[36:2];;PG[38:7];;PC[33:3];PE[36:3]	0.004	0.007	2.77	Glycerophospholipid metabolism
PC[33:5];PE[36:5];;PC[31:2];PE[34:2];;PA[38:6]	0.004	0.009	2.88	Glycerophospholipid metabolism
PC(22:4/P-18:1);PC(22:5/P-18:0);PC(o-18:0/22:6);PC(P-18:0/22:5);PC(P-18:1/22:4);PC(P-20:0/20:5);;PC(20:1/P-18:1);PC(20:2/P-18:0);PC(22:2/P-16:0);PC(O-18:0/20:3);PC(o-20:0/18:3);PC(P-16:0/22:2);PC(P-18:0/20:2);PC(P-18:1/20:1);PC(P-20:0/18:2);;PC(O-14:0/22:0);PC(o-16:0/20:0);PC(o-18:0/18:0);PC(O-20:0/16:0);PE(O-18:0/21:0);PE(O-20:0/19:0)	0.004	0.007	2.93	Glycerophospholipid metabolism
PS(O-18:0/21:0);PS(O-20:0/19:0);PC(18:0/P-18:0);PC(20:0/P-16:0);PC(O-16:0/20:1);PC(o-16:1/20:0);PC(O-18:0/18:1);PC(o-18:1/18:0);PC(O-20:0/16:1);PC(P-16:0/20:0);PC(P-18:0/18:0);PC(P-20:0/16:0);PE(O-20:0/19:1);PE(P-18:0/21:0);PE(P-20:0/19:0);;PC(20:2/P-18:1);PC(20:3/P-18:0);PC(O-16:0/22:4);PC(o-18:0/20:4);PC(O-20:0/18:4);PC(P-18:0/20:3);PC(P-18:1/20:2);PC(P-20:0/18:3)	0.004	0.009	2.94	Glycerophospholipid metabolism
PC(16:0/P-18:0);PC(18:0/P-16:0);PC(O-16:0/18:1);PC(o-16:1/18:0);PC(O-	0.004	0.007	2.96	Glycerophospholipid metabolism

18:0/16:1);PC(o-18:1/16:0);PC(O-20:0/14:1);PC(P-16:0/18:0);PC(P-18:0/16:0);PC(P-20:0/14:0);PE(O-18:0/19:1);PE(O-20:0/17:1);PE(P-16:0/21:0);PE(P-18:0/19:0);PE(P-20:0/17:0);;PA(P-20:0/19:1)				
PC(18:1/P-18:1);PC(18:2/P-18:0);PC(20:2/P-16:0);PC(O-16:0/20:3);PC(O-18:0/18:3);PC(o-18:1/18:2);PC(P-16:0/20:2);PC(P-18:0/18:2);PC(P-18:1/18:1);;PC(18:0/O-16:0);PC(o-16:0/18:0);PC(O-17:0/17:0);PC(O-18:0/16:0);PC(O-20:0/14:0);PE(O-16:0/21:0);PE(O-18:0/19:0);PE(O-20:0/17:0)	0.004	0.007	2.98	Glycerophospholipid metabolism
PC(18:2/P-18:1);PC(18:3/P-18:0);PC(20:3/P-16:0);PC(o-16:0/20:4);PC(O-18:0/18:4);PC(o-18:2/18:2);PC(P-16:0/20:3);PC(P-18:0/18:3);PC(P-18:1/18:2);;PC(16:0/P-18:0);PC(18:0/P-16:0);PC(O-16:0/18:1);PC(o-16:1/18:0);PC(O-18:0/16:1);PC(o-18:1/16:0);PC(O-20:0/14:1);PC(P-16:0/18:0);PC(P-18:0/16:0);PC(P-20:0/14:0);PE(O-18:0/19:1);PE(O-20:0/17:1);PE(P-16:0/21:0);PE(P-18:0/19:0);PE(P-20:0/17:0)	0.004	0.007	3.23	Glycerophospholipid metabolism
PC(o-14:0/16:0);PC(O-16:0/14:0);PC(O-18:0/12:0);PE(O-16:0/17:0);PE(O-18:0/15:0);PE(O-20:0/13:0);;PA(O-16:0/19:1);PA(O-18:0/17:1);PA(O-20:0/15:1);PA(P-16:0/19:0);PA(P-18:0/17:0);PA(P-20:0/15:0)	0.004	0.007	3.53	Glycerophospholipid metabolism
PC(20:2/P-18:1);PC(20:3/P-18:0);PC(O-16:0/22:4);PC(o-18:0/20:4);PC(O-20:0/18:4);PC(P-18:0/20:3);PC(P-18:1/20:2);PC(P-20:0/18:3)	0.004	0.007	3.69	Glycerophospholipid metabolism
PI[20:4];;PI[18:1]	0.002	0.007	3.86	Glycerophospholipid metabolism
PC(22:2/P-18:1);PC(O-18:0/22:4);PC(o-20:0/20:4);PC(P-18:1/22:2);PC(P-20:0/20:3);;PC(20:0/P-18:0);PC(22:0/P-16:0);PC(O-16:0/22:1);PC(o-16:1/22:0);PC(O-18:0/20:1);PC(o-18:1/20:0);PC(O-20:0/18:1);PC(P-16:0/22:0);PC(P-18:0/20:0);PC(P-20:0/18:0);PE(P-20:0/21:0)	0.004	0.007	3.89	Glycerophospholipid metabolism
PC(22:4/P-18:0);PC(O-18:0/22:5);PC(O-	0.004	0.007	3.90	Glycerophospholipi

20:0/20:5);PC(o-20:1/20:4);PC(P-18:0/22:4);PC(P-20:0/20:4);;PC(20:0/P-18:1);PC(20:1/P-18:0);PC(22:1/P-16:0);PC(O-16:0/22:2);PC(O-18:0/20:2);PC(o-18:1/20:1);PC(o-18:2/20:0);PC(O-20:0/18:2);PC(P-16:0/22:1);PC(P-18:0/20:1);PC(P-18:1/20:0);PC(P-20:0/18:1)				d metabolism
LysoPC[24:0]	0.004	0.007	4.11	Glycerophospholipid metabolism
PC(o-22:2/22:3);;PC(24:0/P-18:1);PC(24:1/P-18:0);PC(o-18:2/24:0);PC(O-20:0/22:2);PC(P-18:0/24:1);PC(P-18:1/24:0);PC(P-20:0/22:1)	0.004	0.007	4.42	Glycerophospholipid metabolism
PC(14:0/P-18:1);PC(14:1/P-18:0);PC(16:1/P-16:0);PC(O-14:0/18:2);PC(o-16:1/16:1);PC(P-14:0/18:1);PC(P-16:0/16:1);PC(P-18:0/14:1);PC(P-18:1/14:0);PE(O-18:0/17:2);PE(P-16:0/19:1);PE(P-18:0/17:1);PE(P-20:0/15:1);PA(P-20:0/17:2)	0.004	0.007	4.53	Glycerophospholipid metabolism
PC(o-22:1/20:4);PC(P-20:0/22:4);;PC(22:0/P-18:1);PC(22:1/P-18:0);PC(24:1/P-16:0);PC(O-18:0/22:2);PC(o-18:2/22:0);PC(O-20:0/20:2);PC(P-16:0/24:1);PC(P-18:0/22:1);PC(P-18:1/22:0);PC(P-20:0/20:1)	0.004	0.007	5.29	Glycerophospholipid metabolism
PI[16:0]	0.002	0.007	5.33	Glycerophospholipid metabolism
PS(O-18:0/19:1);PS(O-20:0/17:1);PS(P-16:0/21:0);PS(P-18:0/19:0);PS(P 20:0/17:0);PC(16:0/P-18:1);PC(16:1/P-18:0);PC(18:1/P-16:0);PC(O-16:0/18:2);PC(P-16:0/18:1);PC(P-18:0/16:1);PC(P-18:1/16:0);PC(P-20:0/14:1);PE(O-20:0/17:2);PE(P-18:0/19:1);PE(P-20:0/17:1);PnC[34:1];;PC(18:3/P-18:1);PC(18:4/dm18:0);PC(18:4/P-18:0);PC(20:4/P-16:0);PC(O-16:0/20:5);PC(o-16:1/20:4);PC(P-16:0/20:4);PC(P-18:0/18:4);PC(P-18:1/18:3)	0.004	0.009	5.43	Glycerophospholipid metabolism
PC(20:0/P-18:1);PC(20:1/P-18:0);PC(22:1/P-16:0);PC(O-16:0/22:2);PC(O-18:0/20:2);PC(o-18:1/20:1);PC(o-18:2/20:0);PC(O-20:0/18:2);PC(P-16:0/22:1);PC(P-18:0/20:1);PC(P-18:1/20:0);PC(P-20:0/18:1)	0.004	0.007	5.45	Glycerophospholipid metabolism

PC(14:0/P-18:0);PC(16:0/P-16:0);PC(O-14:0/18:1);PC(o-16:0/16:1);PC(O-18:0/14:1);PC(P-16:0/16:0);PC(P-18:0/14:0);PC(P-20:0/12:0);PE(O-16:0/19:1);PE(O-18:0/17:1);PE(O-20:0/15:1);PE(P-16:0/19:0);PE(P-18:0/17:0);PE(P-20:0/15:0);;PA(O-20:0/17:2);PA(P-18:0/19:1);PA(P-20:0/17:1)	0.004	0.007	5.46	Glycerophospholipid metabolism
PC(15:0/P-18:1);PC(O-16:0/17:2);PC(P-16:0/17:1);PC(P-18:0/15:1);PC(P-18:1/15:0);PE(18:0/P-18:1);PE(18:1/P-18:0);PE(20:1/P-16:0);PE(dm18:0/18:1);PE(O-16:0/20:2);PE(O-18:0/18:2);PE(P-16:0/20:1);PE(P-18:0/18:1);PE(P-18:1/18:0);PE(P-20:0/16:1)	0.004	0.009	5.52	Glycerophospholipid metabolism
PC[34:1];PE[37:1]	0.004	0.009	5.86	Glycerophospholipid metabolism
PC(O-14:0/22:0);PC(o-16:0/20:0);PC(o-18:0/18:0);PC(O-20:0/16:0);PE(O-18:0/21:0);PE(O-20:0/19:0)	0.004	0.007	5.99	Glycerophospholipid metabolism
PC(14:0/P-16:0);PC(o-14:0/16:1);PC(o-16:0/14:1);PC(P-16:0/14:0);PC(P-18:0/12:0);PE(15:0/P-18:0);PE(O-16:0/17:1);PE(O-18:0/15:1);PE(P-16:0/17:0);PE(P-18:0/15:0);PE(P-20:0/13:0)	0.004	0.007	6.34	Glycerophospholipid metabolism
PI[18:2]	0.002	0.007	6.79	Glycerophospholipid metabolism
PC(22:1/P-18:1);PC(22:2/P-18:0);PC(O-20:0/20:3);PC(o-22:0/18:3);PC(P-18:0/22:2);PC(P-18:1/22:1);PC(P-20:0/20:2);;PC(o-16:0/22:0);PC(o-18:0/20:0);PC(O-20:0/18:0);PE(O-20:0/21:0)	0.004	0.007	6.87	Glycerophospholipid metabolism
PE(P-16:0e/0:0)	0.002	0.006	6.90	Glycerophospholipid metabolism
PS[22:4];;PS[20:1];;PG[22:6];PC[17:2]	0.002	0.006	8.44	Glycerophospholipid metabolism
PC(O-16:0/O-18:1);PC(O-18:1/O-16:0)	0.004	0.007	8.49	Glycerophospholipid metabolism
PS[18:2];;PG[18:4]	0.002	0.006	9.61	Glycerophospholipid metabolism
PS(O-20:0/17:2);PS(P-18:0/19:1);PS(P-20:0/17:1);PC(16:0/O-16:0);PC(O-14:0/18:0);PC(O-16:0/16:0);PC(O-18:0/14:0);PC(O-20:0/12:0);PE(O-	0.004	0.009	10.49	Glycerophospholipid metabolism

16:0/19:0);PE(O-18:0/17:0);PE(O-20:0/15:0);;PC(16:1/P-18:1);PC(18:2/P-16:0);PC(O-16:0/18:3);PC(o-16:1/18:2);PC(P-16:0/18:2);PC(P-18:1/16:1);PE(P-20:0/17:2)				
PA[17:2]	0.002	0.007	11.42	Glycerophospholipid metabolism
PG[20:4];;PG[18:1];PA[20:1];PA(20:4e/2:0);PA[22:4]	0.002	0.007	11.90	Glycerophospholipid metabolism
PI[16:1]	0.002	0.007	11.97	Glycerophospholipid metabolism
PG[18:2];PA[20:2]	0.002	0.007	12.50	Glycerophospholipid metabolism
PS(O-16:0/19:1);PS(O-18:0/17:1);PS(O-20:0/15:1);PS(P-16:0/19:0);PS(P-18:0/17:0);PS(P-20:0/15:0);;PG(P-18:0/17:2);PC(14:0/P-18:1);PC(14:1/P-18:0);PC(16:1/P-16:0);PC(O-14:0/18:2);PC(o-16:1/16:1);PC(P-14:0/18:1);PC(P-16:0/16:1);PC(P-18:0/14:1);PC(P-18:1/14:0);PE(O-18:0/17:2);PE(P-16:0/19:1);PE(P-18:0/17:1);PE(P-20:0/15:1)	0.004	0.009	13.62	Glycerophospholipid metabolism
PS[32:1];;PG[32:3];;PC[29:2];PE[32:2]	0.004	0.009	14.11	Glycerophospholipid metabolism
PS(P-20:0/21:0);PC(20:0/P-18:1);PC(20:1/P-18:0);PC(22:1/P-16:0);PC(O-16:0/22:2);PC(O-18:0/20:2);PC(o-18:1/20:1);PC(o-18:2/20:0);PC(O-20:0/18:2);PC(P-16:0/22:1);PC(P-18:0/20:1);PC(P-18:1/20:0);PC(P-20:0/18:1);;PC(22:4/P-18:0);PC(O-18:0/22:5);PC(O-20:0/20:5);PC(o-20:1/20:4);PC(P-18:0/22:4);PC(P-20:0/20:4)	0.004	0.009	14.38	Glycerophospholipid metabolism
PS(O-20:0/19:1);PS(P-18:0/21:0);PS(P-20:0/19:0);PC(18:0/P-18:1);PC(18:1/P-18:0);PC(20:1/P-16:0);PC(O-16:0/20:2);PC(o-18:0/18:2);PC(o-18:1/18:1);PC(P-16:0/20:1);PC(P-18:0/18:1);PC(P-18:1/18:0);PC(P-20:0/16:1);PE(P-20:0/19:1);;PC(20:3/P-18:1);PC(20:4/P-18:0);PC(22:4/P-16:0);PC(O-16:0/22:5);PC(O-18:0/20:5);PC(o-18:1/20:4);PC(P-16:0/22:4);PC(P-18:0/20:4);PC(P-18:1/20:3);PC(P-20:0/18:4)	0.004	0.009	14.41	Glycerophospholipid metabolism

PI[18:0]	0.002	0.006	15.72	Glycerophospholipid metabolism
PI[22:4];;PI[20:1]	0.002	0.007	16.31	Glycerophospholipid metabolism
LysoPC[22:4]	0.002	0.006	17.02	Glycerophospholipid metabolism
PC(O-18:1/O-1:0)	0.002	0.006	18.39	Glycerophospholipid metabolism
PS(O-16:0/17:0);PS(O-18:0/15:0);PS(O-20:0/13:0);;PG(O-16:0/17:2);PG(P-16:0/17:1);PG(P-18:0/15:1)	0.004	0.009	18.41	Glycerophospholipid metabolism
LysoPE[16:1]	0.002	0.007	20.21	Glycerophospholipid metabolism
PG(O-16:0/19:1);PG(O-18:0/17:1);PG(O-20:0/15:1);PG(P-16:0/19:0);PG(P-18:0/17:0);PG(P-20:0/15:0);;PC(16:0/O-16:0);PC(O-14:0/18:0);PC(O-16:0/16:0);PC(O-18:0/14:0);PC(O-20:0/12:0);PE(O-16:0/19:0);PE(O-18:0/17:0);PE(O-20:0/15:0)	0.004	0.009	22.18	Glycerophospholipid metabolism
PS[18:2];;PG[18:4];PC[13:0];;LysoPE[18:3]	0.002	0.007	22.41	Glycerophospholipid metabolism
PS[18:1];;PG[18:3]	0.002	0.006	24.41	Glycerophospholipid metabolism
PS[32:1];;PG[32:3]	0.004	0.007	25.69	Glycerophospholipid metabolism
PG[18:1];PA[20:1]	0.002	0.007	27.37	Glycerophospholipid metabolism
PS(O-16:0/19:0);PS(O-18:0/17:0);PS(O-20:0/15:0);;PG(O-18:0/17:2);PG(P-16:0/19:1);PG(P-18:0/17:1);PG(P-20:0/15:1);;PC(14:0/P-18:0);PC(16:0/P-16:0);PC(O-14:0/18:1);PC(o-16:0/16:1);PC(O-18:0/14:1);PC(P-16:0/16:0);PC(P-18:0/14:0);PC(P-20:0/12:0);PE(O-16:0/19:1);PE(O-18:0/17:1);PE(O-20:0/15:1);PE(P-16:0/19:0);PE(P-18:0/17:0);PE(P-20:0/15:0)	0.004	0.009	44.49	Glycerophospholipid metabolism
PI[20:3];;PI[18:0];;PG[20:0]	0.002	0.007	45.52	Glycerophospholipid metabolism
PI[18:1]	0.002	0.007	56.16	Glycerophospholipid metabolism
PC(O-17:0/20:4);PE(22:2/P-18:1);PE(O-18:0/22:4);PE(O-20:0/20:4);PE(P-	0.004	0.007	68.26	Glycerophospholipi

18:1/22:2);PE(P-20:0/20:3);;PC(O-16:0/19:1);PC(O-17:0/18:1);PC(O-18:0/17:1);PC(O-20:0/15:1);PC(P-16:0/19:0);PC(P-18:0/17:0);PC(P-20:0/15:0);PE(20:0/dm18:0);PE(22:0/P-16:0);PE(O-16:0/22:1);PE(O-18:0/20:1);PE(O-20:0/18:1);PE(P-16:0/22:0);PE(P-18:0/20:0);PE(P-20:0/18:0);;PA(P-20:0/22:4)				d metabolism
PC(18:0/P-18:1);PC(18:1/P-18:0);PC(20:1/P-16:0);PC(O-16:0/20:2);PC(o-18:0/18:2);PC(o-18:1/18:1);PC(P-16:0/20:1);PC(P-18:0/18:1);PC(P-18:1/18:0);PC(P-20:0/16:1);PE(P-20:0/19:1)	0.004	0.007	434.54	Glycerophospholipid metabolism
Phosphoserine	0.004	0.009	0.40	Glycine, serine and threonine metabolism AND cysteine and methionine metabolism
Allothreonine;Homoserine	0.004	0.007	2.63	Glycine, serine and threonine metabolism AND cysteine and methionine metabolism
Lipoyllysine	0.002	0.007	0.14	Glycolysis metabolism
Lactic acid;Dihydroxyacetone	0.002	0.007	1.94	Glycolysis metabolism
Glyceraldehyde 3-phosphate;Dihydroxyacetone phosphate	0.002	0.007	2.06	Glycolysis metabolism
Imidazoleacetic acid ribotide	0.004	0.009	0.31	Histidine metabolism
Histidinol	0.004	0.009	0.42	Histidine metabolism
3-Hydroxy-N6,N6,N6-trimethyl-L-lysine	0.004	0.007	2.70	Lysine metabolism
Ne,Ne dimethyllysine	0.002	0.006	3.00	Lysine metabolism
Nicotinamide riboside	0.004	0.009	1.56	Nicotine and nicotinate metabolism
3-Pyridinebutanoic acid	0.002	0.006	1.79	Nicotine and nicotinate

				metabolism
1-(beta-D-Ribofuranosyl)-1,4-dihydronicotinamide	0.002	0.006	1.82	Nicotine and nicotinate metabolism
2'-Hydroxynicotine;Nicotine-1'-N-oxide;Pseudooxynicotine	0.004	0.009	1.98	Nicotine and nicotinate metabolism
Niacinamide	0.002	0.006	2.15	Nicotine and nicotinate metabolism
(R) 2,3-Dihydroxy-3-methylvalerate;Glycerol 1-propanoate;Mevalonic acid	0.002	0.006	0.00	Other class
Furoic acid	0.002	0.007	0.07	Other class
3-Methylthiopropionic acid;Methyl 2-(methylthio)acetate	0.002	0.007	0.19	Other class
3-Oxoalanine	0.004	0.009	0.30	Other class
2-Isopropyl-3-oxosuccinate;3-oxopimelic acid;Shikimic acid;;2-Keto-6-aminocaproate;4-Acetamidobutanoic acid;5-Amino-3-oxohexanoate;Allysine;Isobutyrylglycine;N-Butyrylglycine	0.004	0.009	0.48	Other class
Phosphatidylinositol-3,4,5-trisphosphate	0.002	0.006	0.58	Other class
Methylamine	0.002	0.007	0.60	Other class
Malondialdehyde;Pyruvaldehyde	0.004	0.007	1.29	Other class
Cis-zeatin	0.002	0.006	1.31	Other class
5-Aminopentanoic acid;Betaine;Norvaline	0.004	0.007	1.33	Other class
Beta-Alanine;Alanine;Sarcosine	0.004	0.007	1.34	Other class
Salsoline;N-Methylsalsolinol	0.004	0.007	1.57	Other class
Biotinyl-5'-AMP	0.004	0.007	1.72	Other class
Serine;Pyruvic acid	0.004	0.007	1.75	Other class
S-Phenylmercapturic acid	0.004	0.007	1.83	Other class
Trimethylaminoacetone	0.004	0.007	2.22	Other class
Hexyl glucoside	0.002	0.006	3.49	Other class
Hexylamine;Triethylamine	0.004	0.007	10.86	Other class
Demethylphylloquinone	0.002	0.007	16.89	Other class

5-Aminopentanoic acid;Betaine;N-Methyl-a-aminoisobutyric acid;Norvaline	0.002	0.006	156.25	Other class
(+/-)-10-HDoHE;(+/-)-11-HDoHE;(+/-)-13-HDoHE;(+/-)-14-HDoHE;(+/-)-16-HDoHE;(+/-)-20-HDoHE;(+/-)-4-HDoHE;(+/-)-7-HDoHE;(+/-)-8-HDoHE;10(11)-EpDPE;13(14)-EpDPE;16(17)-EpDPE;17-HDoHE;19(20)-EpDPE;"1a,1b-dihomo-15-deoxy-delta-12,14-PGJ2";21-HDHA;7(8)-EpDPE;HDHA	0.002	0.007	0.08	Oxidised fatty acids including prostaglandins
Hydroxypentadecanedioic acid;Oxotetradecanoic acid	0.004	0.009	0.22	Oxidised fatty acids including prostaglandins
1(3)-glyceryl-6-keto-PGF1alpha;2-glyceryl-6-keto-PGF1alpha	0.002	0.006	0.29	Oxidised fatty acids including prostaglandins
11,18-di-HEPE";12-epi-12-J2-IsoP;12-J2-IsoP;12-Keto-leukotriene B4;"12,18-di-HEPE";"14,15-dehydro-LTB4";15-A2c-IsoP;15-A2t-IsoP;"15-deoxy-delta-12,14-PGD2";15-epi-15-A2c-IsoP;15-epi-15-A2t-IsoP;15-epi-15-J2-IsoP;15-J2-IsoP;"15-Keto-13,14-dihydroprostaglandin A2";5-epi-5-J2-IsoP;5-J2-IsoP;5-Oxo-6-trans-leukotriene B4;5-trans-PGA2;"5,6-EpHETE";8-epi-15-A2c-IsoP;8-epi-15-J2-IsoP;8-epi-8-J2-IsoP;8-J2-IsoP;"8,15-diepi-15-A2c-IsoP";"8,15-diepi-15-J2c-IsoP";"8,15-diepi-15-J2t-IsoP";"8,18-di-HEPE";8alpha-8-Hydroxy-12-oxo-13-abieten-18-oic acid;Dehydropinifolic acid;Delta-12-Prostaglandin J2;ent-12-epi-12-J2-IsoP;ent-12-J2-IsoP;ent-15-epi-15-J2-IsoP;ent-15-J2-IsoP;ent-5-epi-5-J2-IsoP;ent-5-J2-IsoP;ent-8-epi-8-J2-IsoP;ent-8-J2-IsoP;HpEPE;Leukotriene B5;PGA2;PGB2;PGC2;"PGE2 1,15-lactone";"PGF3alpha-1,15-lactone";Prostaglandin A2;Prostaglandin B2;Prostaglandin J2;Resolvin E2;Resolvin E3	0.002	0.006	0.50	Oxidised fatty acids including prostaglandins
keto-pentadecanoic acid	0.002	0.007	0.53	Oxidised fatty acids including prostaglandins
Hydroxy-decadienediynoic acid	0.004	0.009	0.53	Oxidised fatty acids including prostaglandins
8-Hydroxy-5,6-octadienoic acid	0.002	0.007	1.35	Oxidised fatty acids

				including prostaglandins
11beta,20-Dihydroxy-3-oxopregn-4-en-21-oic acid";17-oxo-Resolvin D1;8-oxo-Resolvin D1	0.002	0.007	1.43	Oxidised fatty acids including prostaglandins
Hydroxydecanedioic acid	0.002	0.007	1.46	Oxidised fatty acids including prostaglandins
12-Oxo-20-trihydroxy-leukotriene B4	0.002	0.006	1.49	Oxidised fatty acids including prostaglandins
PGF2alpha-11-acetate methyl ester;;"11-deoxy-16,16-dimethyl-PGE2";"16,16-dimethyl-PGA1	0.002	0.007	1.54	Oxidised fatty acids including prostaglandins
11-dehydro-TXB3;"12-oxo-10,11-dihydro-20-COOH-LTB4";12-oxo-20-dihydroxy-leukotriene B4;19-hydroxy-Resolvin E1;20-Carboxy-leukotriene B4;20-hydroxy-Resolvin E1	0.002	0.006	1.54	Oxidised fatty acids including prostaglandins
12-Oxo-20-carboxy-leukotriene B4;11-Hydroxy-9,15,16-trioxooctadecanoic acid";"2,3-Dinor-6-keto-prostaglandin F1 a";"2,3-Dinor-TXB2";"2,3-dinor, 6-keto-PGF1alpha	0.002	0.006	1.77	Oxidised fatty acids including prostaglandins
Hydroxyoctanoic acid	0.004	0.007	1.99	Oxidised fatty acids including prostaglandins
Omega-Carboxy-trinor-leukotriene B4	0.002	0.006	2.01	Oxidised fatty acids including prostaglandins
3-Hydroxydodecanedioic acid	0.002	0.007	2.39	Oxidised fatty acids including prostaglandins
Lipoxin C4	0.004	0.007	2.79	Oxidised fatty acids including prostaglandins
Hydroxy-octadecadienoic acid;Oxo-octadecenoic acid	0.002	0.006	3.92	Oxidised fatty acids including prostaglandins
10-HOME(8);10-Oxooctadecanoic acid;11-HOME(9);11-Oxooctadecanoic acid;12-HOME;13-HpOME;17-HOME;19-HOME;3-Oxooctadecanoic acid;4-keto stearic acid;5-Hexyltetrahydro-2-furanoctanoic acid;5-HOME(2);5-	0.002	0.007	4.56	Oxidised fatty acids including prostaglandins

Oxooctadecanoic acid;6-keto stearic acid;7-keto-stearic acid;8-HOME(9);9-HOME;9-HOME(12);9-Oxooctadecanoic acid;epoxy-stearic acid;HOME;hydroxy-oleic acid				
hydroxy-bromooctadecatrienoic acid	0.002	0.007	4.77	Oxidised fatty acids including prostaglandins
Hydroxytetradecanedioic acid	0.002	0.007	5.05	Oxidised fatty acids including prostaglandins
10,11-dihydro-20-dihydroxy-LTB4;20-Hydroxy-PGF2a;20-hydroxy-PGF2alpha;6-Keto-prostaglandin F1a;hydroxy-PGE1;hydroxy-PGF2alpha;Prostaglandin G1;Thromboxane B2	0.002	0.006	8.41	Oxidised fatty acids including prostaglandins
(-)-11-Hydroxy-9,15,16-trioxooctadecanoic acid";"2,3-Dinor-6-keto-prostaglandin F1 a";"2,3-Dinor-TXB2";"2,3-dinor, 6-keto-PGF1alpha	0.002	0.007	8.80	Oxidised fatty acids including prostaglandins
Hydroxyhexadecanoic acid	0.002	0.006	9.13	Oxidised fatty acids including prostaglandins
3,11-dihydroxy myristoic acid	0.002	0.006	9.40	Oxidised fatty acids including prostaglandins
10,11-dihydro-12-oxo-LTB4";11-deoxy-PGE2;11-HpETE;"11,12-DiHETE";"11H-14,15-EETA";11HPETE;12HPETE;12Leukotriene B4;"14,15-DiHETE";"14,15-Dihydroxy-8(17),13(16)-labdadien-19-oic acid";"14,15-HxA3 ";"14,15-HxB3 ";15-epi-PGA1;"15H-11,12-EETA";15HPETE;"17,18-DiHETE";5-HPETE;"5,12-DiHETE";"5,15-DiHETE";5Hydroperoxyeicosatetraenoic acid;6-trans-12-epi-Leukotriene B4;6-trans-Leukotriene B4;"6,7-dihydro-5-oxo-12-epi-LTB4";8-iso-PGA1;"8,15-DiHETE";"8,9-DiHETE";8HPETE;9-HpETE;HpETE;Leukotriene B4;PGC1;"PGF2alpha-1,11-lactone";"PGF2alpha-1,15-lactone";"PGF2alpha-1,9-lactone";Prostaglandin A1;Prostaglandin B1	0.002	0.006	10.82	Oxidised fatty acids including prostaglandins
Hydroxydodecanoic acid	0.002	0.007	10.95	Oxidised fatty acids including prostaglandins

trihydroxy-stearic acid	0.002	0.006	11.16	Oxidised fatty acids including prostaglandins
HETE di-endoperoxide	0.002	0.006	11.44	Oxidised fatty acids including prostaglandins
Hydroxy-tetradecanoic acid	0.002	0.007	11.63	Oxidised fatty acids including prostaglandins
5,6-Dihydroxyprostaglandin F1a;;"2,3-Dinor-TXB1	0.002	0.006	13.14	Oxidised fatty acids including prostaglandins
2,15-dihydroxy-pentadecylic acid	0.002	0.006	13.48	Oxidised fatty acids including prostaglandins
11-dehydro-2,3-dinor-TXB2	0.002	0.007	14.24	Oxidised fatty acids including prostaglandins
dihydroxy-palmitic acid	0.002	0.007	14.75	Oxidised fatty acids including prostaglandins
13,14-dihydrohydroxyPGE1;"8,8a-Deoxyoleandolide";hydroxy-PGF1alpha;TXB1	0.002	0.007	15.39	Oxidised fatty acids including prostaglandins
10,11-dihydro-20-trihydroxy-leukotriene B4	0.002	0.007	17.58	Oxidised fatty acids including prostaglandins
11-Dehydro-thromboxane B2;11-dehydro-TXB2;19-Hydroxy-PGE2;"20-COOH-10,11-dihydro-LTB4";20-dihydroxyleukotriene B4;20-hydroxy-PGD2;20-Hydroxy-PGE2;"5,12-diHPETE";"5,15-diHPETE";5(6)-Epoxy Prostaglandin E1;6-Ketoprostaglandin E1;"6,15-Diketo,13,14-dihydro-PGF1a";"8,15-diHPETE;"D17, 6-keto PGF1a";diHPETE;hydroperoxy-PGD2;hydroperoxy-PGE2;Prostaglandin G2;TXB3	0.002	0.006	19.15	Oxidised fatty acids including prostaglandins
10,11-dihydro-12-oxo-Resolvin E1";12-Oxo-20-hydroxy-leukotriene B4;15-dehydro-prostaglandin I2;15-Epi-lipoxin B5;15-keto-PGD2;15-Keto-prostaglandin E2;15-Oxo-lipoxin A4;15-oxo-LXA4;19-hydroxy-PGA2;19-hydroxy-PGB2;20-oxo-leukotriene B4;"5,6,18-triHEPE";"6-trans-5,12,18-triHEPE";8-iso-15-keto-	0.002	0.006	19.50	Oxidised fatty acids including prostaglandins

PGE2;HpHEPE;hydroxy-PGA2;hydroxy-PGB2;PGH3;PGI3;PGK2;Prostaglandin D3;Prostaglandin E3;Resolvin E1;TXA3				
Dihydroxyoctadecanoic acid	0.002	0.006	21.65	Oxidised fatty acids including prostaglandins
10,11-dihydro-leukotriene B4;11-deoxy-PGE1;11-deoxy-PGF2a;11-deoxy-PGF2beta;"11,12-DiHETrE";12-Keto-tetrahydro-leukotriene B4;"14,15-DiHETrE";"5,6-DHET";"6,7-dihydro-12-epi-LTB4";"8,9-DiHETrE";cis-epoxyhydroxy-eicosa-dienoic acid	0.002	0.006	25.04	Oxidised fatty acids including prostaglandins
20-HETE-d6;;"10,11-dihydro-20-dihydroxy-LTB4";20-Hydroxy-PGF2a;20-hydroxy-PGF2alpha;6-Keto-prostaglandin F1a;hydroxy-PGE1;hydroxy-PGF2alpha;Prostaglandin G1;Thromboxane B2	0.002	0.007	30.54	Oxidised fatty acids including prostaglandins
(+/-)-11-HEPE;(+/-)-15-HEPE;(+/-)-18-HEPE;(+/-)-8-HEPE;(+/-)-9-HEPE;(ent-15beta)-15-Hydroxy-19-trachylobanoic acid;11(12)-EpETE;12-HEPE;12-KETE;"14,15-EpETE";"14,15-LTA4";15-HEPE;15-KETE;15d-PGA1;"17,18-EpETE";4,5-LTA4";5-HEPE;5-KETE;8(9)-EpETE	0.002	0.006	36.12	Oxidised fatty acids including prostaglandins
Hydroxydecanoic acid	0.002	0.007	315.27	Oxidised fatty acids including prostaglandins
17-phenyl-trinor-PGF2alpha amide	0.004	0.009	549.92	Oxidised fatty acids including prostaglandins
Pantothenol	0.004	0.009	0.22	Pantothenate and CoA biosynthesis
Hydroxyprolyl-Isoleucine;Hydroxyprolyl-Leucine	0.004	0.007	0.00	Peptide
Glutamyl-Glycine;Glycyl-Glutamate	0.004	0.009	0.00	Peptide
Glutaminyl-Lysine;Lysyl-Gamma-glutamate	0.002	0.007	0.02	Peptide
Dityrosine;Arginyl-Gamma-glutamate;Arginyl-Glutamine	0.002	0.007	0.09	Peptide
5-L-Glutamyl-taurine	0.002	0.007	0.20	Peptide
Lysyl-Phenylalanine	0.002	0.007	0.27	Peptide

Arginyl-Proline;Histidinyl-Valine	0.002	0.006	0.31	Peptide
Ornithino-L-alanine	0.004	0.007	0.43	Peptide
aspartyl-glutamic acid	0.004	0.009	0.54	Peptide
prolyl-proline	0.004	0.009	0.54	Peptide
Hydroxyprolyl-Tryptophan	0.002	0.007	1.72	Peptide
Aspartyl-Aspartate	0.004	0.009	1.91	Peptide
Phenylalanyl-Serine;Tyrosyl-Alanine;;Methionyl-Proline	0.004	0.007	2.02	Peptide
Cysteinyl-Isoleucine;Cysteinyl-Leucine	0.002	0.007	2.02	Peptide
Aspartyl-Phenylalanine	0.004	0.009	2.13	Peptide
Isoleucyl-Methionine;Leucyl-Methionine	0.004	0.007	2.33	Peptide
Histidinyl-Phenylalanine;Isoleucyl-Isoleucine	0.002	0.007	3.10	Peptide
Prolylhydroxyproline	0.004	0.007	3.81	Peptide
Isoleucyl-Phenylalanine;Leucyl-phenylalanine	0.004	0.007	4.12	Peptide
Histidinyl-Tryptophan	0.004	0.009	4.80	Peptide
Glutamyl-Valine	0.002	0.006	5.09	Peptide
Aspartyl-Histidine;Histidinyl-Aspartate;Prolylhydroxyproline	0.002	0.006	5.34	Peptide
Asparaginyl-Hydroxyproline	0.002	0.007	5.60	Peptide
Valyl-Asparagine	0.004	0.009	5.74	Peptide
Alanyl-Serine;Glycyl-Threonine	0.004	0.009	6.95	Peptide
Prolyl-Valine	0.004	0.007	7.80	Peptide
Tryptophyl-Tyrosine	0.004	0.009	8.08	Peptide
Alanyl-Glutamate;Glutamyl-Alanine	0.004	0.009	8.18	Peptide
Histidinyl-Glycine	0.004	0.009	8.53	Peptide
Tryptophyl-Valine	0.002	0.006	9.70	Peptide
Lysyl-Glutamate	0.002	0.007	9.77	Peptide
Glutaminyl-Tryptophan	0.002	0.006	11.53	Peptide
Valyl-Hydroxyproline	0.002	0.006	13.79	Peptide
Histidinyl-Isoleucine;Histidinyl-Leucine	0.004	0.007	28.42	Peptide
Gamma-Aminobutyryl-lysine	0.002	0.006	29.71	Peptide

Aspartyl-Isoleucine;Aspartyl-Leucine	0.004	0.009	39.38	Peptide
Tyrosyl-Valine;Threoninyl-Histidine	0.002	0.006	49.41	Peptide
Anserine;Homocarnosine	0.002	0.007	50.16	Peptide
Cysteinyl-Cysteine	0.002	0.007	131.14	Peptide
phenylalanyl-proline	0.004	0.007	167.22	Peptide
AICAR	0.004	0.009	0.01	Purine and pyrimidine metabolism
6,8-Dihydroxypurine;Oxypurinol;Xanthine	0.004	0.009	0.36	Purine and pyrimidine metabolism
Guanosine diphosphate	0.002	0.007	0.48	Purine and pyrimidine metabolism
5-Aminoimidazole	0.002	0.006	0.49	Purine and pyrimidine metabolism
Pyrimidine	0.002	0.007	0.53	Purine and pyrimidine metabolism
Adenosine 3',5'-diphosphate;ADP;dGDP	0.002	0.007	0.55	Purine and pyrimidine metabolism
Uridine 5'-diphosphate	0.002	0.007	0.65	Purine and pyrimidine metabolism
5'-Methylthioadenosine	0.004	0.007	0.77	Purine and pyrimidine metabolism
Cytosine	0.004	0.007	1.32	Purine and pyrimidine metabolism
Dihydrothymine	0.004	0.007	1.60	Purine and pyrimidine metabolism
Orotidine	0.004	0.009	1.92	Purine and pyrimidine metabolism
5-Methyldeoxycytidine	0.004	0.009	2.49	Purine and pyrimidine metabolism

Cytidine	0.004	0.007	3.53	Purine and pyrimidine metabolism
Thymidine	0.004	0.009	8.60	Purine and pyrimidine metabolism
1-Methylguanosine;2-Methylguanosine;5'-Deoxyadenosine;Deoxyadenosine	0.004	0.007	10.61	Purine and pyrimidine metabolism
Methylguanosine	0.004	0.009	18.81	Purine and pyrimidine metabolism
3-Methyluridine;Imidazoleacetic acid riboside;Ribothymidine	0.004	0.009	21.78	Purine and pyrimidine metabolism
SAICAR	0.002	0.007	22.08	Purine and pyrimidine metabolism
7-Methylguanosine	0.002	0.007	26.87	Purine and pyrimidine metabolism
Pseudouridine;Uridine	0.004	0.009	58.04	Purine and pyrimidine metabolism
4-Oxoretinal	0.002	0.006	1.29	Retinoic acid metabolism
cis-Aconitic acid;Dehydroascorbic acid;trans-Aconitic acid	0.002	0.006	0.00	Short chain organic acid
2-Hydroxy-3-methylpentanoic acid;Hydroxyhexanoic acid	0.004	0.007	0.00	Short chain organic acid
3-Methyladipic acid;Dimethylglutaric acid";Pimelic acid	0.004	0.007	0.00	Short chain organic acid
Butyric acid;Isobutyric acid	0.002	0.006	0.03	Short chain organic acid
Citric acid;Isocitric acid	0.002	0.006	0.03	Short chain organic acid
Oxoglutaric acid	0.004	0.009	0.28	Short chain organic acid
2-Amino-5-hydroxypentanoic acid;2-Methylacetoacetic acid;2-Oxovaleric acid	0.004	0.007	0.28	Short chain organic acid
3-Hydroxysuberic acid	0.004	0.009	0.75	Short chain organic acid

Butenoic acid	0.004	0.007	1.25	Short chain organic acid
2-Hydroxy-3-methylbutanoic acid;Sebacic acid	0.002	0.006	1.39	Short chain organic acid
D-2,3-Dihydroxypropanoic acid;Glyceric acid	0.004	0.009	1.41	Short chain organic acid
Oxoadipic acid;Oxoadipic acid;5-Amino-2-oxopentanoic acid;5-Aminolevulinic acid	0.004	0.009	1.42	Short chain organic acid
Hydroxypyruvic acid;Malonic acid	0.004	0.009	1.43	Short chain organic acid
Dimethylmalonic acid;Ethylmalonic acid;Glutaric acid;Methylsuccinic acid	0.002	0.007	1.49	Short chain organic acid
Fumaric acid;Maleic acid	0.002	0.007	1.51	Short chain organic acid
2-Hydroxybutyric acid;3-Hydroxybutyric acid;4-Hydroxybutyric acid	0.002	0.007	1.59	Short chain organic acid
4-Hydroxy-2-oxobutanoic acid;Methylmalonic acid;Succinic acid	0.002	0.007	1.74	Short chain organic acid
Malic acid	0.002	0.007	1.76	Short chain organic acid
Oxalacetic acid	0.002	0.006	1.85	Short chain organic acid
2-Methylacetoacetic acid;2-Oxovaleric acid	0.002	0.007	2.71	Short chain organic acid
Methyl-oxovaleric acid;Oxohexanoic acid	0.002	0.007	2.87	Short chain organic acid
2-Hydroxyadipic acid;3-Hydroxyadipic acid;3-hydroxymethyl-glutaric acid	0.004	0.007	37.07	Short chain organic acid
1alpha,23,25,26-tetrahydroxycholecalciferol;" 6,19-epidioxy-1alpha,24-di hydroxy-6,19-dihydrocholecalciferol";" 6,19-epidioxy-1alpha,25-dihydroxy-6,19-dihydrocholecalciferol";"1alpha,26-Tetrahydroxyvitamin D3";2-deoxyecdysone;25-deoxyecdysone;"3alpha,7alpha,12alpha-trihydroxy-5alpha-cholesten-26-oic acid";"3alpha,7alpha,12alpha-Trihydroxy-5beta-cholesten-26-oic acid	0.004	0.007	0.12	Sterol and steroid metabolism
1alpha,25-dihydroxy-2alpha-(3-hydroxypropoxy)-19-norcholecalciferol;" 1alpha,25-dihydroxy-2beta-(3-	0.002	0.006	0.15	Sterol and steroid metabolism

hydroxypropoxy)-19-norcholecalciferol";;" 1alpha,25-dihydroxy-26,27-dimethyl-22,22,23,23-tetradehydro-24a-homo-20-epicholecalciferol";" 1alpha,25-dihydroxy-26,27-dimethyl-22,22,23,23-tetradehydro-24a-homocholecalciferol";" 1alpha,25-dihydroxy-26,27-dimethyl-22,23,24,24a-tetradehydro-24a-homocholecalciferol";"1alpha,24-Dihydroxy-22-ene-24-cyclopropylvitamin D3";"3beta-3-Hydroxy-11-oxolanosta-8,24-dien-26-al				
16a-Hydroxyestrone;16b-Hydroxyestrone;2-Hydroxyestrone;4-Hydroxyestrone	0.002	0.006	0.24	Sterol and steroid metabolism
Dehydroepiandrosterone 3-glucuronide;Dehydroisoandrosterone 3-glucuronide;Testosterone glucuronide	0.002	0.006	0.32	Sterol and steroid metabolism
18-Oxocortisol	0.002	0.007	0.35	Sterol and steroid metabolism
25-hydroxy-16,17,23,23,24,24-hexadehydrocholecalciferol	0.002	0.006	0.37	Sterol and steroid metabolism
25-Azacholesterol	0.004	0.007	0.39	Sterol and steroid metabolism
24-Hydroxy-19-norgeminivitamin D3	0.004	0.007	0.39	Sterol and steroid metabolism
3-Deoxy-3-azido-25-hydroxyvitamin D3	0.002	0.006	0.46	Sterol and steroid metabolism
pregn-4-en-3-one	0.002	0.007	0.56	Sterol and steroid metabolism
1alpha,25-dihydroxy-22-oxacholecalciferol 3-hemiglutarate;;7-oxotyphasterol	0.004	0.007	0.63	Sterol and steroid metabolism
Chenodeoxycholic acid 3-sulfate;Chenodeoxycholic acid sulfate;Ursodeoxycholic acid 3-sulfate	0.004	0.007	0.72	Sterol and steroid metabolism
Estradiol	0.002	0.006	1.32	Sterol and steroid metabolism
12-Ketodeoxycholic acid;7-Hydroxy-3-oxocholanoic acid;7a-Hydroxy-3-oxo-5b-cholanoic acid;9'-Carboxy-alpha-chromanol;Nutriacholic acid;;" 1alpha-hydroxy-24,25,26,27-tetranorcholecalciferol";" 22-hydroxy-24,25,26,27-tetranorcholecalciferol";" 23-hydroxy-24,25,26,27-tetranorcholecalciferol";1alpha-Hydroxy-2-	0.004	0.007	1.42	Sterol and steroid metabolism

methylene-19-norbishomopregnacalciferol;"22-hydroxy-23,24,25,26,27-pentanorcholecalciferol";"22-hydroxy-24,25,26,27-tetranorcholecalciferol				
Norepinephrine sulfate	0.002	0.007	1.74	Sterol and steroid metabolism
Campesteryl ester[18:2];Campesteryl linoleate;episteryl oleate;fecosteryl oleate;lanosteryl palmitoleate	0.004	0.007	1.86	Sterol and steroid metabolism
6-dehydrotestosterone;Androstenedione;Dehydrotestosterone	0.002	0.006	2.44	Sterol and steroid metabolism
1alpha,23,25,26-tetrahydroxycholecalciferol";" 6,19-epidioxy-1alpha,24-di hydroxy-6,19-dihydrocholecalciferol";" 6,19-epidioxy-1alpha,25-dihydroxy-6,19-dihydrocholecalciferol";"1alpha,26-Tetrahydroxyvitamin D3";"3alpha,7alpha,12alpha-trihydroxy-5alpha-cholesten-26-oic acid";"3alpha,7alpha,12alpha-Trihydroxy-5beta-cholesten-26-oic acid	0.002	0.007	2.68	Sterol and steroid metabolism
1alpha,21-dihydroxy-20-oxo-22,23,24,25,26,27-hexanorcholecalciferol;11-deoxycortisol;19-Hydroxydeoxycorticosterone;21-Deoxycortisol;21-Hydroxy-5b-pregnane-3,11,20-trione";7'-Carboxy-alpha-tocotrienol;Corticosterone	0.002	0.006	4.33	Sterol and steroid metabolism
12-Methoxy-8,11,13-abietatrien-20,11-olide;"9,11alpha-epoxypregn-4-ene-3,20-dione	0.004	0.007	4.54	Sterol and steroid metabolism
3a,12b-Dihydroxy-5b-cholanoic acid";"3a,7a-Dihydroxycholanoic acid";"3alpha,15alpha-dihydroxy-5beta-cholan-24-oic acid";"3b,12a-Dihydroxy-5a-cholanoic acid";"3b,12a-Dihydroxy-5b-cholanoic acid";"3b,12b-Dihydroxy-5b-cholanoic acid";"3b,7a-Dihydroxy-5b-cholanoic acid";"7a,12b-dihydroxy-5b-Cholan-24-oic acid";"7b,12a-Dihydroxycholanoic acid";Allochenodeoxycholic acid;Allodeoxycholic acid;Avideoxycholic acid;Chenodeoxycholic acid;Deoxycholic acid;Hyodeoxycholic acid;Isodeoxycholic	0.002	0.007	6.62	Sterol and steroid metabolism

acid;Isohyodeoxycholic acid;Isoursodeoxycholic acid;Murocholic acid;Ursodeoxycholic acid				
beta-tocotrienol;epsilon-Tocopherol;Gamma-Tocotrienol	0.002	0.006	10.73	Sterol and steroid metabolism
5a-Dihydrotestosterone sulfate;Androsterone sulfate	0.002	0.007	12.32	Sterol and steroid metabolism
Estrone sulfate	0.002	0.007	14.89	Sterol and steroid metabolism
1alpha-hydroxy-24-(dimethylphosphoryl)-25,26,27-trinorcholecalciferol	0.002	0.007	15.46	Sterol and steroid metabolism
Dehydroepiandrosterone sulfate;Epitestosterone sulfate;Testosterone sulfate	0.002	0.007	19.93	Sterol and steroid metabolism
1alpha,25-dihydroxy-11alpha-phenylcholecalciferol";" 1alpha,25-dihydroxy-11beta-phenylcholecalciferol	0.002	0.006	23.84	Sterol and steroid metabolism
pregnenolone sulfate	0.002	0.007	30.09	Sterol and steroid metabolism
1alpha,25-dihydroxy-24a-homo-26,27-dimethyl-22-thiacholecalciferol;" 1alpha,25-dihydroxy-24a,24b,24c-trihomo-22-thia-20-epicholecalciferol";" 1alpha,25-dihydroxy-24a,24b,24c-trihomo-22-thiacholecalciferol";" 1alpha,25-dihydroxy-26,27-dimethyl-24a-homo-22-thia-20-epicholecalciferol	0.002	0.007	44.49	Sterol and steroid metabolism
26,27-Dihomo-1alpha-hydroxy-24-epivitamin D2";"26,27-Dihomo-1alpha-hydroxyvitamin D2	0.002	0.007	48.88	Sterol and steroid metabolism
17-Hydroxypregnenolone sulfate	0.002	0.007	114.50	Sterol and steroid metabolism
1alpha,25-dihydroxy-26,27-dimethyl-22,22,23,23-tetradehydro-24a-homo-20-epicholecalciferol;" 1alpha,25-dihydroxy-26,27-dimethyl-22,22,23,23-tetradehydro-24a-homocholecalciferol";" 1alpha,25-dihydroxy-26,27-dimethyl-22,23,24,24a-tetradehydro-24a-homocholecalciferol";"1alpha,24-Dihydroxy-22-ene-24-cyclopropylvitamin D3	0.002	0.007	117.25	Sterol and steroid metabolism
25-hydroxycholecalciferol 6,19-sulfur dioxide adduct";7-Dehydrocholesterol-3-	0.004	0.009	8389.90	Sterol and steroid metabolism

sulfate ester;Vitamin D3 sulfoconjugate				
2-Methoxy-1,4-benzoquinone	0.002	0.007	0.52	Ubiquinone metabolism
3-Hexaprenyl-4,5-Dihydroxybenzoic acid	0.004	0.007	3.73	Ubiquinone metabolism
1,2-Benzoquinone;Quinone	0.004	0.007	39.59	Ubiquinone metabolism
Pyridoxine	0.002	0.006	2.28	Vitamin B metabolism
Isopyridoxal;Pyridoxal	0.004	0.007	2.98	Vitamin B metabolism

Supplementary Table 6B

**Significantly altered (q<0.01) metabolites in cell media (KD siRNA vs SCR siRNA)**	**p value**	**q value**	**Fold change (KD siRNA to SCR siRNA)**	**Metabolite Class**
3-Methylcrotonylglycine;Tiglylglycine	0.002	0.006	0.52	Acyl amino acid
Butyrylcarnitine;Isobutyrylcarnitine	0.002	0.006	0.70	Acyl carnitine
Creatine	0.002	0.006	0.58	Arginine and proline metabolism
Arginine	0.002	0.006	0.76	Arginine and proline metabolism
Proline	0.002	0.006	0.76	Arginine and proline metabolism
Homo-arginine;Targinine	0.002	0.006	3.73	Arginine and proline metabolism
3-Methoxy-4,5-methylenedioxybenzoic acid	0.002	0.006	0.04	Aromatic metabolites and associated

				metabolism
Acetyl-N-formyl-5-methoxykynurenamine;Alpha-N-Phenylacetyl-L-glutamine	0.002	0.006	0.06	Aromatic metabolites and associated metabolism
Tryptamine	0.002	0.006	0.54	Aromatic metabolites and associated metabolism
Trihydroxybenzene	0.002	0.006	0.64	Aromatic metabolites and associated metabolism
Tryptophan	0.002	0.006	0.70	Aromatic metabolites and associated metabolism
Tyramine	0.002	0.006	0.70	Aromatic metabolites and associated metabolism
3-Methyldioxyindole;4-(3-Pyridyl)-3-butenoic acid	0.002	0.006	0.74	Aromatic metabolites and associated metabolism
4-Hydroxybenzaldehyde	0.002	0.006	0.77	Aromatic metabolites and associated metabolism
3-Methoxytyrosine;Methyldopa	0.002	0.006	0.78	Aromatic metabolites and associated metabolism
Beta-Leucine;Leucine;isoleucine;Norleucine	0.002	0.006	0.73	BCAA metabolism
4-O-Methyl-myo-inositol	0.002	0.006	0.48	Carbohydrate
Allose;Glucose;Galactose;Fructose;Mannose;Tagatose;Sorbose;Myoinositol	0.002	0.006	0.76	Carbohydrate
Deoxyerythronic acid;Deoxythreonic acid	0.002	0.006	0.76	Carbohydrate
Glucose;Galactose;Fructose;Mannose;Tagatose;Sorbose;Myoinositol	0.002	0.006	0.76	Carbohydrate
Neuraminic acid;;Glutamyl-Valine;N-Acetylglucosamine;N-Acetyl-galactosamine	0.002	0.006	1.87	Carbohydrate
N-Acetyl-galactosamine;N-Acetyl-glucosamine	0.002	0.006	2.46	Carbohydrate
N-Acetyl-9-O-lactoylneuraminic acid	0.002	0.006	101.09	Carbohydrate
Lactose;Maltose;Epimelibiose;Galabiose;Galactinol;Isomaltose;Lactulose;Mannobiose;Neotrehalose;Trehalose;Turanose	0.002	0.006	107.52	Carbohydrate

Cysteine-S-sulfate	0.002	0.006	0.31	Cysteine and methionine metabolism
Cystine	0.002	0.006	0.48	Cysteine and methionine metabolism
Dodecanoic acid	0.002	0.006	0.17	Fatty acid
Tetradecadienoic acid	0.002	0.006	0.29	Fatty acid
Undecanedioic acid	0.002	0.006	0.55	Fatty acid
Tetradecanedioic acid	0.002	0.006	1.55	Fatty acid
Dodecenoic acid	0.002	0.006	1.84	Fatty acid
Butanol;Isobutanol	0.002	0.006	0.66	Fatty alcohol
5-Methyltetrahydrofolic acid	0.002	0.006	0.16	Folate metabolism
Lactoyltetrahydropterin;Dihydrobiopterin	0.002	0.006	0.61	Folate metabolism
Glutamine	0.002	0.006	0.79	Glutamine and glutamate metabolism
Glycine	0.002	0.006	0.42	Glycine, serine and threonine metabolism
Phosphoserine	0.002	0.006	0.53	Glycine, serine and threonine metabolism
Allothreonine;Homoserine	0.002	0.006	0.20	Glycine, serine and threonine metabolism AND cysteine and methionine metabolism
1-Methylhistidine;3-Methylhistidine	0.002	0.006	0.46	Histidine metabolism
Histamine	0.002	0.006	0.68	Histidine metabolism
Quinolinic acid	0.002	0.006	0.20	Nicotine and nicotinate metabolism
Nicotinamide N-oxide	0.002	0.006	0.67	Nicotine and nicotinate metabolism
3-Pyridinebutanoic acid	0.002	0.006	0.78	Nicotine and nicotinate metabolism
Malondialdehyde;Pyruvaldehyde	0.002	0.006	0.76	Other class
(3b,6b,8a,12a)-8,12-Epoxy-7(11)-eremophilene-6,8,12-trimethoxy-3-ol";"2,3-dinor-11b-PGF2alpha";"2,3-Dinor-8-iso-PGF2alpha";"2,3-dinor-PGE1";"9K,12,13-diHODE";Dinor-PGF2alpha	0.002	0.006	0.44	Oxidised fatty acids including prostaglandins

Methionyl-Serine	0.002	0.006	0.22	Peptide
Threoninyl-Tryptophan	0.002	0.006	0.37	Peptide
Asparaginyl-Methionine	0.002	0.006	0.43	Peptide
Allocystathionine;Cysteinyl-Threonine;Cystathionine	0.002	0.006	0.54	Peptide
Isoleucyl-Phenylalanine;Leucyl-phenylalanine	0.002	0.006	0.58	Peptide
Valyl-Tryptophan	0.002	0.006	0.62	Peptide
Asparaginyl-Histidine;Serinyl-Lysine	0.002	0.006	0.63	Peptide
Glutaminyl-Threonine;Threoninyl-Gamma-glutamate	0.002	0.006	0.63	Peptide
Glutamyl-Proline	0.002	0.006	0.74	Peptide
Cysteinyl-Gamma-glutamate;Cysteinyl-Glutamine	0.002	0.006	0.79	Peptide
Histidinyl-Glycine	0.002	0.006	3.33	Peptide
Lysyl-Asparagine	0.002	0.006	14.68	Peptide
Arginyl-Glutamate;Glutamyl-Arginine;;Arginyl-Asparagine	0.002	0.006	17.32	Peptide
Deoxyuridine	0.002	0.006	0.15	Purine and pyrimidine metabolism
Hypoxanthine	0.002	0.006	0.17	Purine and pyrimidine metabolism
Deoxycytidine	0.002	0.006	0.30	Purine and pyrimidine metabolism
2-Hydroxyglutaric acid	0.002	0.006	0.37	Short chain organic acid
2-Hydroxyadipic acid;3-Hydroxyadipic acid;3-hydroxymethyl-glutaric acid	0.002	0.006	0.74	Short chain organic acid
(R) 2,3-Dihydroxy-3-methylvalerate;Mevalonic acid	0.002	0.006	0.77	Short chain organic acid
11b-Hydroxyandrost-4-ene-3,17-dione";"16a-Hydroxyandrost-4-ene-3,17-dione";"19-Hydroxyandrost-4-ene-3,17-dione";19-Oxotestosterone;2-Hydroxyestradiol-3-methyl ether;2-Methoxyestradiol;4-Methoxy-17beta-estradiol;"7a-Hydroxyandrost-4-ene-3,17-dione	0.002	0.006	0.38	Sterol and steroid metabolism
Norepinephrine sulfate	0.002	0.006	0.53	Sterol and steroid

				metabolism
16-Glutaryloxy-1alpha,25-dihydroxy-20-epivitamin D3;"16-Glutaryloxy-1alpha,25-dihydroxyvitamin D5	0.002	0.006	0.72	Sterol and steroid metabolism
Dehydrotestosterone	0.002	0.006	0.77	Sterol and steroid metabolism
25-hydroxycholecalciferol 6,19-sulfur dioxide adduct";7-Dehydrocholesterol-3-sulfate ester;Vitamin D3 sulfoconjugate	0.002	0.006	40.23	Sterol and steroid metabolism
25,26,27-trinorcholecalciferol 24-carboxylic acid	0.002	0.006	45.72	Sterol and steroid metabolism
16-Dehydroprogesterone	0.002	0.006	175.97	Sterol and steroid metabolism
Dihydroneopterin phosphate;;Threoneopterin;Neopterin	0.002	0.006	0.17	Vitamin B metabolism

Supplementary Table 6C

**Significantly altered (p<0.05) intracellular metabolites KD shRNA vs SCR shRNA**	**p value**	**q value**	**Fold change**	**Metabolite class**
N-Decanoylglycine	0.041	0.506	0.56	Acyl amino acids
Butyrylcarnitine;Isobutyrylcarnitine	0.015	0.373	1.22	Acyl carnitine
Docosapentaenoyl carnitine	0.004	0.068	1.27	Acyl carnitine
Cervonyl carnitine	0.015	0.482	1.41	Acyl carnitine
2,6 Dimethylheptanoyl carnitine;Nonanoylcarnitine	0.009	0.291	2.35	Acyl carnitine
Hydroxy-hexadecenoylcarnitine	0.026	0.586	11.57	Acyl carnitine
Creatine	0.015	0.373	1.21	Arginine and proline metabolism
Proline	0.009	0.291	1.22	Arginine and proline metabolism
Homo-L-arginine	0.002	0.149	1.55	Arginine and proline metabolism
Spermine	0.002	0.149	2.15	Arginine and proline metabolism and

				glutathione metabolism
Glutamine	0.009	0.380	1.21	Arginine/proline metabolism OR Glyoxylate and dicarboxylate metabolism OR purine/pyrimidine metabolism
Dimethoxyphenylethylamine	0.009	0.380	0.40	Aromatic amino acid and related metabolism
Dimethoxybenzoic acid";3-(3-Hydroxyphenyl)-3-hydroxypropanoic acid;3-Methoxy-4-hydroxyphenylglycolaldehyde;Homovanillic acid;Hydroxyphenyllactic acid;Isohomovanillic acid	0.015	0.482	0.40	Aromatic amino acid and related metabolism
N'-Formylkynurenine;6-Hydroxy-1H-indole-3-acetamide	0.002	0.149	1.24	Aromatic amino acid and related metabolism
5-Hydroxy-L-tryptophan	0.002	0.195	1.30	Aromatic amino acid and related metabolism
5-Hydroxyindoleacetic acid;1H-Indole-3-carboxaldehyde	0.002	0.195	1.34	Aromatic amino acid and related metabolism
Sinapic acid;4-Methoxycinnamic acid	0.030	0.220	1.37	Aromatic amino acid and related metabolism
3,4-Methylenedioxybenzoic acid;Benzoquinoneacetic acid;Terephthalic acid	0.041	0.506	1.44	Aromatic amino acid and related metabolism
Formyl-5-hydroxykynurenamine;Kynurenine	0.002	0.149	1.45	Aromatic amino acid and related metabolism
Serotinose	0.030	0.220	1.22	Carbohydrate metabolism
Glycero-D-galacto-heptitol	0.017	0.178	1.67	Carbohydrate metabolism
Lactose;Maltose;Epimelibiose;Galabiose;Galactinol;Inulobiose;Isomaltose;Lactulose;Mannobiose;Neotrehalose;Trehalose;Turanose	0.009	0.157	1.92	Carbohydrate metabolism
PE-Cer(d14:1/21:0);PE-	0.017	0.150	1.41	Ceramides and

Cer(d15:1/20:0);PE-Cer(d16:1/19:0);SM(d16:1/16:0);SM(d18:1/14:0)				sphingolipids
3-Dehydrosphinganine;"5-hydroxy-sphingosine";(d18:1) sphingosine;Sphingosine	0.004	0.213	1.47	Ceramides and sphingolipids
PE-Cer(d14:2/25:0);PE-Cer(d15:2/24:0);PE-Cer(d16:2/23:0);SM(d16:1/20:1);SM(d18:0/18:2);SM(d18:1/18:1);SM(d18:2/18:0);SM(d19:1/17:1)	0.004	0.068	1.51	Ceramides and sphingolipids
SM(d18:1/22:1);SM(d18:2/22:0)	0.004	0.068	1.57	Ceramides and sphingolipids
SM(d16:1/22:1);SM(d18:1/20:1);SM(d18:2/20:0)	0.009	0.095	1.97	Ceramides and sphingolipids
Homoserine	0.004	0.153	1.20	Cysteine and methionine metabolism
Glutathione	0.041	0.648	1.25	Cysteine and methionine metabolism
Tuberonic acid	0.017	0.178	0.76	Fatty acid
Methylheptanoic acid;Caprylic acid	0.002	0.149	1.36	Fatty acid
Decenedioic acid	0.026	0.465	1.31	Fatty acid
Linolenyl stearate;Linoleyl oleate;Oleyl linoleate;Stearyl linolenate	0.009	0.095	1.23	Fatty acid esters
Behenyl linolenate;Linolenyl behenate	0.030	0.209	1.34	Fatty acid esters
Arachidyl myristate;Behenyl laurate;Lauryl behenate;Myristyl arachidate;Palmityl stearate;Stearyl palmitate	0.017	0.150	1.41	Fatty acid esters
Arachidyl linoleate;Linoleyl arachidate	0.004	0.068	1.43	Fatty acid esters
Nonadienol	0.015	0.373	0.49	Fatty alcohol
Dodecadienol	0.015	0.373	1.47	Fatty alcohol
Pentenol	0.002	0.149	2.19	Fatty alcohol
PA[42:10];;PA[40:7];;PG(O-16:0/18:3);PG(P-16:0/18:2);;PA(O-16:0/20:3);PA(O-18:0/18:3);PA(P-16:0/20:2);PA(P-18:0/18:2)	0.030	0.209	0.39	Glycerophospholipids
PG[39:3];;PG(O-18:0/22:6);PG(P-20:0/20:5);;PA[41:3];;PA(O-20:0/22:6)	0.030	0.220	0.68	Glycerophospholipids

PC[34:5];PE[37:5];;PC[32:2];PE[35:2];;PA[39:6]	0.004	0.068	0.73	Glycerophospholipids
PG[34:1];PA[36:1]	0.004	0.153	0.73	Glycerophospholipids
PC[36:6];PE[39:6];;PC[34:3];PE[37:3];;PA[41:7];;PC[32:0];PE(18:0(10(R)Me)/16:0);PE[35:0]	0.004	0.068	0.79	Glycerophospholipids
PG(P-18:0/22:6);;PG[37:1];;PG(O-16:0/22:4);PG(O-18:0/20:4);PG(O-20:0/18:4);PG(P-18:0/20:3);PG(P-20:0/18:3);;PA(P-20:0/22:6);;PA[39:1];;PA(O-18:0/22:4);PA(O-20:0/20:4);PA(P-20:0/20:3)	0.004	0.153	0.80	Glycerophospholipids
PC[34:1];PE[37:1];;PA[39:2];;PE-NMe2(O-16:0/O-16:0)	0.004	0.068	1.20	Glycerophospholipids
PS[33:1];;PG[33:3];PC[30:2];PE[33:2]	0.030	0.220	1.20	Glycerophospholipids
PC[39:5];PE[42:5];;PC[37:2];PE[40:2];;PA[44:6];;PC(O-16:0/O-18:1);PC(O-18:1/O-16:0)	0.009	0.095	1.21	Glycerophospholipids
PS[35:0];PG[35:2];;PC[32:1];PE-NMe[34:1];PE[35:1]	0.004	0.153	1.21	Glycerophospholipids
PS[33:0];PG[33:2];PC[30:1];PE[33:1]	0.009	0.157	1.21	Glycerophospholipids
PC[28:0];PE[31:0];;PA[33:1]	0.004	0.068	1.21	Glycerophospholipids
PC[37:5];PE[40:5];;PC[35:2];PE-NMe2[36:2];PE[38:2];;PA[42:6]	0.004	0.068	1.21	Glycerophospholipids
PC[42:5];;PC[40:2];PE[43:2]	0.004	0.068	1.21	Glycerophospholipids
PC[31:1];PE[34:1];PA[36:2];;PC(O-14:0/O-14:0)	0.009	0.095	1.21	Glycerophospholipids
PE(20:4/P-18:1);PE(20:5/P-18:0);PE(22:5/P-16:0);PE(O-16:0/22:6);PE(P-16:0/22:5);PE(P-18:0/20:5);PE(P-18:1/20:4);;PC(P-16:0/17:2);PE(18:1/P-18:1);PE(18:2/P-18:0);PE(20:2/P-16:0);PE(O-16:0/20:3);PE(O-18:0/18:3);PE(P-16:0/20:2);PE(P-18:0/18:2);PE(P-18:1/18:1);;PA(P-18:0/22:6)	0.004	0.068	1.22	Glycerophospholipids
PE(20:3/P-18:1);PE(20:4/P-18:0);PE(22:4/P-16:0);PE(O-16:0/22:5);PE(O-18:0/20:5);PE(O-18:1/20:4);PE(P-16:0/22:4);PE(P-18:0/20:4);PE(P-18:1/20:3);PE(P-20:0/18:4);;PC(15:0/P-18:1);PC(O-16:0/17:2);PC(P-16:0/17:1);PC(P-	0.004	0.068	1.22	Glycerophospholipids

18:0/15:1);PC(P-18:1/15:0);PE(18:0/P-18:1);PE(18:1/P-18:0);PE(20:1/P-16:0);PE(dm18:0/18:1);PE(O-16:0/20:2);PE(O-18:0/18:2);PE(P-16:0/20:1);PE(P-18:0/18:1);PE(P-18:1/18:0);PE(P-20:0/16:1);;PA(O-18:0/22:6);PA(P-20:0/20:5)				
PC[29:1];PE[32:1];;PA[34:2]	0.004	0.153	1.22	Glycerophospholipids
PC(O-15:0/20:4);PE(20:2/P-18:1);PE(20:3/P-18:0);PE(O-16:0/22:4);PE(O-18:0/20:4);PE(O-20:0/18:4);PE(P-18:0/20:3);PE(P-18:1/20:2);PE(P-20:0/18:3);;PC(15:0/P-18:0);PC(O-16:0/17:1);PC(O-18:0/15:1);PC(P-16:0/17:0);PC(P-18:0/15:0);PC(P-20:0/13:0);PE(18:0/P-18:0);PE(20:0/P-16:0);PE(dm18:0/18:0);PE(O-16:0/20:1);PE(O-18:0/18:1);PE(O-20:0/16:1);PE(P-16:0/20:0);PE(P-20:0/16:0);;PA(O-20:0/20:5);PA(P-18:0/22:4);PA(P-20:0/20:4)	0.004	0.153	1.23	Glycerophospholipids
PC(14:0/P-18:0);PC(16:0/P-16:0);PC(O-14:0/18:1);PC(o-16:0/16:1);PC(O-18:0/14:1);PC(P-16:0/16:0);PC(P-18:0/14:0);PC(P-20:0/12:0);PE(O-16:0/19:1);PE(O-18:0/17:1);PE(O-20:0/15:1);PE(P-16:0/19:0);PE(P-18:0/17:0);PE(P-20:0/15:0);;PA(O-20:0/17:2);PA(P-18:0/19:1);PA(P-20:0/17:1)	0.004	0.068	1.23	Glycerophospholipids
PC[32:1];PE-NMe[34:1];PE[35:1];;PA[37:2];PE(O-16:0/O-16:0)	0.004	0.068	1.24	Glycerophospholipids
PC[37:4];PE[40:4];;PC[35:1];PE[38:1];;PA[42:5];PC(O-16:0/O-16:0)	0.004	0.068	1.24	Glycerophospholipids
PE(O-18:0/O-18:0);PE(O-20:0/O-16:0)	0.009	0.095	1.25	Glycerophospholipids
PC[36:1];PE[39:1];;PA[41:2];;PE(O-18:0/O-18:0);PE(O-20:0/O-16:0)	0.004	0.068	1.25	Glycerophospholipids
PS[38:4];;PS[36:1];;DGCC[36:5];;PG[38:6];;PC[33:2];PE[36:2]	0.017	0.150	1.27	Glycerophospholipids
PC(o-14:0/16:0);PC(O-16:0/14:0);PC(O-18:0/12:0);PE(O-16:0/17:0);PE(O-18:0/15:0);PE(O-20:0/13:0);;PA(O-16:0/19:1);PA(O-18:0/17:1);PA(O-20:0/15:1);PA(P-16:0/19:0);PA(P-	0.004	0.068	1.27	Glycerophospholipids

18:0/17:0);PA(P-20:0/15:0)				
PE(22:4/P-18:1);PE(22:5/dm18:0);PE(22:5/P-18:0);PE(O-18:0/22:6);PE(P-18:0/22:5);PE(P-18:1/22:4);PE(P-20:0/20:5);;PC(P-18:0/17:2);PE(20:1/P-18:1);PE(20:2/P-18:0);PE(22:2/P-16:0);PE(O-18:0/20:3);PE(O-20:0/18:3);PE(P-16:0/22:2);PE(P-18:0/20:2);PE(P-18:1/20:1);PE(P-20:0/18:2);;PA(P-20:0/22:6);;PC(O-16:0/17:0);PC(O-18:0/15:0);PC(O-20:0/13:0);PE(O-16:0/20:0);PE(O-18:0/18:0);PE(O-20:0/16:0)	0.004	0.068	1.27	Glycerophospholipids
PC[44:12];;PI[36:3]	0.017	0.150	1.29	Glycerophospholipids
PE(22:4/P-18:0);PE(O-20:0/20:5);PE(P-18:0/22:4);PE(P-20:0/20:4);;PC(O-18:0/17:2);PC(P-16:0/19:1);PC(P-18:0/17:1);PC(P-20:0/15:1);PE(20:0/P-18:1);PE(20:1/P-18:0);PE(22:1/P-16:0);PE(O-16:0/22:2);PE(O-18:0/20:2);PE(O-20:0/18:2);PE(P-16:0/22:1);PE(P-18:0/20:1);PE(P-18:1/20:0);PE(P-20:0/18:1);;PA(O-20:0/22:6)	0.004	0.068	1.29	Glycerophospholipids
PS[38:3];;PS[36:0];;PG[38:5];PC[33:1];PE-NMe2[34:1];PE[36:1]	0.030	0.209	1.29	Glycerophospholipids
PC(15:0/P-18:0);PC(O-16:0/17:1);PC(O-18:0/15:1);PC(P-16:0/17:0);PC(P-18:0/15:;PC(P-20:0/13:0);PE(18:0/P-18:0);PE(20:0/P-16:0);PE(dm18:0/18:0);PE(O-16:0/20:1);PE(O-18:0/18:1);PE(O-20:0/16:1);PE(P-16:0/20:0);PE(P-20:0/16:0);;	0.004	0.068	1.30	Glycerophospholipids
PC[30:2];PE[33:2];;PA[35:3]	0.004	0.068	1.30	Glycerophospholipids
PS[40:4];;PS[38:1];;PG[40:6];;PC[35:2];PE-NMe2[36:2];PE[38:2];;PC[37:5];PE[40:5]	0.030	0.220	1.31	Glycerophospholipids
PI[36:4]	0.004	0.068	1.32	Glycerophospholipids
PS[36:1];PG[36:3]	0.030	0.209	1.33	Glycerophospholipids
PI[38:4]	0.009	0.095	1.35	Glycerophospholipids
LysoPC[15:0];LysoPE[18:0];PC(14:0/O-1:0);PC(7:0/O-8:0)	0.004	0.213	1.35	Glycerophospholipids
PS[40:5];;PS[38:2];;PG[40:7];;PC[35:3];PE[38:3];;PC[33:0];PE[36:0];PC[37:6];PE(P-	0.009	0.157	1.35	Glycerophospholipids

18:0/22:6(14OH));PE[40:6]				
PI[34:1]	0.030	0.220	1.36	Glycerophospholipids
PS[21:0];;LysoPC[18:1];PC(O-16:1/2:0);PC(P-16:0/2:0)	0.002	0.195	1.36	Glycerophospholipids
PS[34:1];;PG[34:3]	0.004	0.068	1.36	Glycerophospholipids
PS[40:2];;PC[38:2];PE[41:2];;PC[37:3];PE[40:3]	0.004	0.068	1.37	Glycerophospholipids
PS[40:4];;PS[38:1];;PG[40:6];;PC[35:2];PE-NMe2[36:2];PE[38:2]	0.004	0.068	1.37	Glycerophospholipids
PS[38:2]	0.004	0.068	1.38	Glycerophospholipids
PG[17:1];LysoPC[14:0];PC(O-12:0/2:0)	0.041	0.648	1.39	Glycerophospholipids
PI[40:5]	0.004	0.068	1.40	Glycerophospholipids
PS[40:2];;PE(P-20:0/22:4);;PC[37:3];PE[40:3];;PC[35:0];PE-NMe2[36:0];PE[38:0]	0.030	0.220	1.41	Glycerophospholipids
PC[28:1];PE[31:1];PA[33:2]	0.004	0.068	1.43	Glycerophospholipids
LysoPE[18:1];PC[15:1]	0.026	0.465	1.44	Glycerophospholipids
PC[44:9]	0.004	0.068	1.47	Glycerophospholipids
PI[40:3];PI[40:4]	0.004	0.068	1.47	Glycerophospholipids
PI[38:3]	0.004	0.068	1.47	Glycerophospholipids
LysoPC[16:1];PC[16:1];PE[19:1]	0.002	0.149	1.49	Glycerophospholipids
PI(O-18:0/20:5);PI(P-16:0/22:4);PI(P-18:0/20:4);PI(P-20:0/18:4);;PI(O-16:0/20:2);PI(O-18:0/18:2);PI(P-16:0/20:1);PI(P-18:0/18:1);PI(P-20:0/16:1);;PG[38:2];PG[40:5]	0.030	0.220	1.53	Glycerophospholipids
LysoPC[18:1];PC(O-16:1/2:0);PC(P-16:0/2:0)	0.004	0.068	1.60	Glycerophospholipids
LysoPE[16:0];PC[13:0]	0.002	0.149	1.62	Glycerophospholipids
PI[32:1]	0.004	0.153	1.81	Glycerophospholipids
PS[38:5];;PS[36:2];;PG[38:7];;PC[33:3];PE[36:3];;PC[31:0];PE-NMe2[32:0];PE[34:0];;PC[35:6];PE(P-16:0/22:6(14OH));PE[38:6]	0.017	0.178	3.94	Glycerophospholipids
Phosphohomoserine;Phosphothreonine	0.026	0.586	1.54	Glycine, serine and threonine metabolism
1,26-Hexacosanediol diferulate	0.026	0.465	0.37	Other class

Hydroxydopamine;Norepinephrine;Pyridoxine;2-(Hydroxymethyl)benzoic acid;Methoxybenzoic acid;3,4-Dihydroxyphenylacetaldehyde";4-Hydroxy-3-methylbenzoic acid;6-Methylsalicylic acid;Mandelic acid;Methyl furfuracrylate;Methylparaben;Ortho-Hydroxyphenylacetic acid;p-Anisic acid;Phenoxyacetic acid	0.041	0.648	0.64	Other class
Imidazoleacetic acid ribotide	0.017	0.178	0.78	Other class
4-Hydroxyglutamate semialdehyde;Glutamic acid;Acetylserine	0.026	0.586	1.22	Other class
Dihydrothymine	0.009	0.095	1.23	Other class
Heptanoylcholine	0.002	0.195	1.24	Other class
Serine;Pyruvic acid	0.017	0.150	1.26	Other class
Acetylhomoserine;Aminoadipic acid;Methylglutaconic acid	0.017	0.178	1.27	Other class
Spermine dialdehyde	0.030	0.209	1.28	Other class
5-Aminopentanoic acid;Betaine;N-Methyl-a-aminoisobutyric acid;Norvaline;;Pentenoic acid	0.017	0.178	1.32	Other class
Triethanolamine	0.009	0.157	1.39	Other class
Cotinine glucuronide	0.002	0.195	1.55	Other class
Methyluridine;Ribothymidine;;S-aminomethyldihydrolipoamide	0.004	0.153	6.38	Other class
ADP;dGDP	0.026	0.586	1.22	Oxidative phosphorylation and purine metabolism
L-prolyl-L-proline	0.030	0.220	0.61	Peptide
Prolyl-Glutamate	0.030	0.209	0.70	Peptide
Glutamyl-aspartic acid	0.030	0.220	0.74	Peptide
Glutamyl-Glycine	0.009	0.157	0.76	Peptide
Cysteinyl-Proline;Prolyl-Cysteine	0.009	0.291	1.29	Peptide
Norophthalmic acid	0.030	0.220	1.29	Peptide
Prolyl-Valine	0.004	0.068	1.40	Peptide
Tryptophyl-Tyrosine	0.009	0.157	1.91	Peptide
Glutamyl-Valine	0.004	0.270	2.14	Peptide
N5-Hexanoylspermidine	0.041	0.506	0.65	Polyamine

				metabolism
Guanosine diphosphate	0.004	0.270	1.22	Purine and pyrimidine metabolism
Uridine 5'-diphosphate	0.002	0.195	1.40	Purine and pyrimidine metabolism
Thymidine	0.030	0.220	1.41	Purine and pyrimidine metabolism
Orotidylic acid	0.004	0.270	1.49	Purine and pyrimidine metabolism
Adenosine;Deoxyguanosine	0.002	0.149	2.14	Purine and pyrimidine metabolism
12-Ketodeoxycholic acid;7-Hydroxy-3-oxocholanoic acid;7a-Hydroxy-3-oxo-5b-cholanoic acid;9'-Carboxy-alpha-chromanol;Nutriacholic acid	0.026	0.465	1.23	Sterol and steroid metabolism
6-dehydrotestosterone;tetramethyl-pentadecahexaenoic acid'';Androstenedione;Dehydrotestosterone	0.009	0.291	1.37	Sterol and steroid metabolism
1alpha,25-dihydroxy-23-azacholecalciferol	0.009	0.095	1.55	Sterol and steroid metabolism
Norepinephrine sulfate	0.004	0.153	2.20	Sterol and steroid metabolism

Supplementary Table 6D

**Significantly altered (p<0.05) metabolites in cell media (KD shRNA vs SCR shRNA)**	**p value**	**q value**	**Fold change (KD shRNA vs SCR shRNA)**	**Metabolite class**
N-stearoyl glutamic acid	0.002	0.084	0.60	Acyl amino acids
N-Acetylhistamine	0.002	0.044	1.23	Acyl amino acids

N-oleoyl alanine;N-palmitoyl proline	0.009	0.139	0.28	Acyl amino acids
N-palmitoyl alanine	0.015	0.169	0.46	Acyl amino acids
N-stearoyl valine	0.026	0.177	0.77	Acyl amino acids
N-Acetylaspartylglutamic acid	0.026	0.186	1.51	Acyl amino acids
N-Acetyl-L-methionine	0.041	0.186	1.31	Acyl amino acids
Tetradecenoylcarnitine	0.002	0.084	0.78	Acyl carnitine
Hydroxypropionylcarnitine	0.004	0.138	1.59	Acyl carnitine
4,8 Dimethylnonanoyl carnitine;Undecanoylcarnitine	0.009	0.139	0.35	Acyl carnitine
Hydroxyhexanoycarnitine	0.026	0.186	1.31	Acyl carnitine
DG(34:1)	0.041	0.278	1.20	Acyl glycerides
Norspermidine	0.041	0.177	0.57	Arginine and proline metabolism
Dimethoxyphenylethylamine	0.002	0.044	0.76	Aromatic amino acid and related metabolism
Formyl-5-hydroxykynurenamine;Kynurenine;;5-Hydroxyindoleacetic acid	0.002	0.055	1.51	Aromatic amino acid and related metabolism
N'-Formylkynurenine;6-Hydroxy-1H-indole-3-acetamide	0.002	0.094	1.54	Aromatic amino acid and related metabolism
5-Hydroxyindoleacetic acid;;1H-Indole-3-carboxaldehyde	0.002	0.094	1.55	Aromatic amino acid and related metabolism
5-Hydroxyindoleacetic acid;;Formyl-5-hydroxykynurenamine;Kynurenine	0.002	0.094	1.59	Aromatic amino acid and related metabolism
Phenylbutyric acid;Benzenebutanoic acid	0.002	0.094	1.81	Aromatic amino acid and related metabolism
5-Hydroxyindoleacetic acid;1H-Indole-3-carboxaldehyde	0.004	0.072	1.36	Aromatic amino acid and related metabolism
Aniline	0.009	0.107	2.03	Aromatic amino acid and related metabolism
3,4-Dihydroxybenzylamine	0.015	0.169	0.75	Aromatic amino acid and related metabolism

Caftaric acid;Cis-Caffeoyl tartaric acid;;Formyl-5-hydroxykynurenamine;Kynurenine	0.041	0.186	0.75	Aromatic amino acid and related metabolism
2(N)-Methyl-norsalsolinol	0.041	0.186	0.79	Aromatic amino acid and related metabolism
2-(Methylamino)benzoic acid;Phenylglycine	0.041	0.278	1.42	Aromatic amino acid and related metabolism
5-Hydroxy-L-tryptophan	0.041	0.186	1.51	Aromatic amino acid and related metabolism
2-Amino-4-hydroxy-3-methylpentanoic acid	0.026	0.180	1.76	Branched chain amino acid metabolism
Methyluric acid	0.026	0.177	0.76	Caffeine metabolism
Lactose;Maltose;Epimelibiose;Galabiose;Galactinol;Inulobiose;Isomaltose;Lactulose;Mannobiose;Neotrehalose;Trehalose;Turanose	0.015	0.150	0.01	Carbohydrate metabolism
Serotinose	0.015	0.169	2.50	Carbohydrate metabolism
2-Phosphoglyceric acid;3-Phosphoglyceric acid	0.026	0.186	0.72	Carbohydrate metabolism
3-ketosphingosine;(d18:2) sphingosine;Sphingadiene	0.002	0.084	0.06	Ceramides and sphingolipids
(d18:3)sphingosine	0.009	0.175	0.26	Ceramides and sphingolipids
Cer(d15:1/20:0);Cer(d18:1/17:0)	0.026	0.177	0.77	Ceramides and sphingolipids
L-threo-sphinganine);Sphinganine	0.041	0.177	0.57	Ceramides and sphingolipids
N-Formyl-L-methionine	0.002	0.094	1.41	Cysteine and methionine metabolism
Cysteine-S-sulfate	0.015	0.150	0.74	Cysteine and methionine metabolism
3-Sulfinylpyruvic acid	0.015	0.186	0.78	Cysteine and methionine metabolism
Thiocysteine	0.026	0.186	1.35	Cysteine and methionine

				metabolism
Cystathionine ketimine	0.041	0.177	0.48	Cysteine and methionine metabolism
5-Methylthioribose	0.041	0.186	1.26	Cysteine and methionine metabolism
Methyl-hexadecadienoic acid;Linoelaidic acid;Linoleic acid;Octadecadienoic acid	0.002	0.084	1.35	Fatty acid
Tetradecadienoic acid	0.002	0.055	1.42	Fatty acid
Methyl-hexadecadienoate	0.002	0.084	1.58	Fatty acid
Methyl-tetradecadienoate	0.004	0.102	1.44	Fatty acid
Pentadecenoic acid	0.015	0.150	0.40	Fatty acid
Traumatic acid;Methyl-decadienoate	0.015	0.150	0.71	Fatty acid
heneicosenoic acid	0.026	0.186	0.29	Fatty acid
tetrabromo-stearic acid	0.026	0.186	0.78	Fatty acid
Tetradecanedioic acid	0.026	0.186	1.26	Fatty acid
Decenedioic acid	0.015	0.150	3.56	Fatty acid
Heptadecynol	0.002	0.084	0.74	Fatty alcohol
Octadienol	0.041	0.186	0.55	Fatty alcohol
Hexylglycerol;Octanol	0.041	0.177	0.63	Fatty alcohol
Undecanol;2,4-Dimethyl-4-nonanol	0.041	0.224	0.75	Fatty alcohol
Elaidamide;Oleamide	0.026	0.177	0.45	Fatty amide
Octadecanamide	0.041	0.177	0.51	Fatty amide
LysoPC[16:1]	0.026	0.186	1.88	Glycerophospholipids
LysoPC[14:0]	0.041	0.177	1.57	Glycerophospholipids
Dihydroxyfumaric acid	0.015	0.169	0.75	Glyoxylate and dicarboxylate metabolism
Urocanic acid	0.002	0.044	0.40	Histidine metabolism
Methylhistidine	0.026	0.180	0.76	Histidine metabolism
Diaminohexanoate;Lysine	0.002	0.084	2.07	Lysine metabolism
N-Methylnicotinamide	0.002	0.044	0.48	Nicotinate and nicotinamide metabolis

1-(beta-D-Ribofuranosyl)-1,4-dihydronicotinamide	0.002	0.044	1.29	Nicotinate and nicotinamide metabolis
2-Dodecylbenzenesulfonic acid	0.002	0.094	0.71	Other class
N-Undecylbenzenesulfonic acid	0.002	0.094	0.71	Other class
Dihydrozeatin-7-N-dihydrozeatin;Dihydrozeatin-9-N-glucoside;Dihydrozeatin-O-glucoside	0.002	0.055	0.80	Other class
Heptanoylcholine	0.004	0.072	1.52	Other class
Methyl-5-acetoxyhexanoate;Azelaic acid;Nonate	0.009	0.122	0.66	Other class
Diaminosalicylic acid;Cinnamic acid;Hydroxy-methylpentanoic acid;Hydroxyhexanoic acid	0.015	0.169	0.73	Other class
Norepinephrine sulfate;;N-Acetyl-S-(N-methylcarbamoyl)cysteine	0.015	0.150	0.77	Other class
Isovalerylglucuronide	0.015	0.150	1.29	Other class
Kinetin-7-N-glucoside;Kinetin-9-N-glucoside	0.015	0.169	1.33	Other class
alhpa-tocopheronolactone;Alpha-CEHC	0.015	0.150	1.94	Other class
Glutamylphenylalanine;Hydroxyprolyl-Tyrosine;Glutamyl-L-phenylalanine;4'-Phosphopantothenate;Isoleucyl-Threonine;Leucyl-Threonine	0.015	0.186	2.27	Other class
Phytosphingosine;Hydroxyoctadecanoic acid	0.026	0.177	0.78	Other class
Arachidyl alcohol;Dihydrophytol;Phytanol	0.041	0.177	0.38	Other class
Ethyl 4-(methylthio)butyrate	0.041	0.186	0.42	Other class
3-isopropenyl-6-oxoheptanoic acid	0.041	0.186	0.69	Other class
10-Nitrolinoleic acid	0.041	0.278	0.76	Other class
Prolylhydroxyproline;Methoxytyrosine;Met hyldopa	0.041	0.186	1.46	Other class
Valproic acid glucuronide	0.041	0.177	7.66	Other class
Ubiquinone-1	0.002	0.094	0.70	Oxidative phosphorylation
FAPy-adenine	0.026	0.180	0.70	Oxidised DNA base
17-phenyl-trinor-PGF2alpha amide	0.002	0.084	1.38	Oxidised fatty acids including

				prostaglandins
Oxohexadecanoic acid	0.004	0.072	1.69	Oxidised fatty acids including prostaglandins
10,11-dihydro-12-oxo-LTB4";11-deoxy-PGE2;11-HpETE;"11,12-DiHETE";"11H-14,15-EETA";11HPETE;12HPETE;12Leukotriene B4;"14,15-DiHETE";"14,15-Dihydroxy-8(17),13(16)-labdadien-19-oic acid";"14,15-HxA3 ";"14,15-HxB3 ";15-epi-PGA1;"15H-11,12-EETA";15HPETE;"17,18-DiHETE";5-HPETE;"5,12-DiHETE";"5,15-DiHETE";5Hydroperoxyeicosatetraenoic acid;6-trans-12-epi-Leukotriene B4;6-trans-Leukotriene B4;"6,7-dihydro-5-oxo-12-epi-LTB4";8-iso-PGA1;"8,15-DiHETE";"8,9-DiHETE";8HPETE;9-HpETE;DiHETE;"ent-1(10)-Halimene-15,19-dioic acid";Hepoxilin A3;Hepoxilin B3;HpETE;Leukotriene B4;PGC1;"PGF2alpha-1,11-lactone";"PGF2alpha-1,15-lactone";"PGF2alpha-1,9-lactone";Prostaglandin A1;Prostaglandin B1;;	0.009	0.107	0.65	Oxidised fatty acids including prostaglandins
Hydroxydodecanedioic acid	0.015	0.186	0.61	Oxidised fatty acids including prostaglandins
keto-pentadecanoic acid	0.015	0.169	0.70	Oxidised fatty acids including prostaglandins
Dihydroxy-pentadecylic acid	0.015	0.169	0.71	Oxidised fatty acids including prostaglandins
5-iPF2alpha-VI;11-beta-PGE1;11-epi-8-F2c-IsoP;11-epi-8-F2t-IsoP;11-Epi-PGF2a;"11,12,15-THETA";"11,12,15-TriHETRE";"11,14,15-THETA";11b-PGF2a;"11beta-13,14-dihydro-15-keto PGF2alpha";12-epi-12-F2c-IsoP;12-epi-12-F2t-IsoP;12-F2c-IsoP;12-F2t-IsoP;"13,14-Dihydro- lipoxin A4";"13,14-Dihydro-15-keto PGF2a";"13,14-dihydro-15-keto-PGE1";15-epi-15-F2c-IsoP;15-epi-15-F2t-IsoP;15-F2c-IsoP;5-epi-12-F2c-IsoP;5-epi-12-F2t-IsoP;5-epi-5-F2c-IsoP;5-epi-5-F2t-IsoP;5-F2c-IsoP;5-F2t-IsoP;"5,12-diepi-12-F2c-IsoP";"5,12-diepi-12-F2t-IsoP";6alpha-	0.026	0.180	1.35	Oxidised fatty acids including prostaglandins

PGI1;6beta-PGI1;8-epi-15-F2c-IsoP;8-epi-5-F2c-IsoP;8-epi-5-F2t-IsoP;8-epi-8-F2c-IsoP;8-epi-8-F2t-IsoP;8-F2c-IsoP;8-F2t-IsoP;"8-iso-13,14-dihydro-15-keto-PGF2a";8-Isoprostaglandin E1;8-Isoprostaglandin F2a;8-isoprostaglandin PGF2b;"8,11-diepi-8-F2c-IsoP";"8,11-diepi-8-F2t-IsoP";"8,15-diepi-15-F2c-IsoP";"8,5-diepi-5-F2c-IsoP";"8,5-diepi-5-F2t-IsoP";ent-11-epi-8-F2c-IsoP;ent-11-epi-8-F2t-IsoP;ent-12-epi-12-F2c-IsoP;ent-12-epi-12-F2t-IsoP;ent-12-F2c-IsoP;ent-12-F2t-IsoP;ent-15-epi-15-F2c-IsoP;ent-15-epi-15-F2t-IsoP;ent-15-F2c-IsoP;ent-15-F2t-IsoP;ent-5-epi-12-F2c-IsoP;ent-5-epi-12-F2t-IsoP;ent-5-epi-5-F2c-IsoP;ent-5-epi-5-F2t-IsoP;ent-5-F2c-IsoP;ent-5-F2t-IsoP;"ent-5,12-diepi-12-F2c-IsoP";"ent-5,12-diepi-12-F2t-IsoP";ent-8-epi-15-F2c-IsoP;ent-8-epi-5-F2c-IsoP;ent-8-epi-5-F2t-IsoP;ent-8-epi-8-F2c-IsoP;ent-8-epi-8-F2t-IsoP;ent-8-F2c-IsoP;ent-8-F2t-IsoP;"ent-8,11-diepi-8-F2c-IsoP";"ent-8,11-diepi-8-F2t-IsoP";"ent-8,15-diepi-15-F2c-IsoP";"ent-8,15-diepi-15-F2t-IsoP";"ent-8,5-diepi-5-F2c-IsoP";"ent-8,5-diepi-5-F2t-IsoP";ent-PGF2alpha;PGE1;PGF2alpha;PGH1;Prostaglandin D1;Prostaglandin E1;Prostaglandin F2a;Prostaglandin F2b;Prostaglandin H1;Sterebin G;Trioxilin A3;Troxilin B3				
Hydroxynonanoic acid	0.026	0.180	1.39	Oxidised fatty acids including prostaglandins
hydroxy-bromooctadecatrienoic acid	0.026	0.180	3.03	Oxidised fatty acids including prostaglandins
Hydroxyisoheptanoic acid	0.041	0.278	0.52	Oxidised fatty acids including prostaglandins
Hydroxydodecanoic acid	0.041	0.224	0.75	Oxidised fatty acids including prostaglandins
HETE di-endoperoxide	0.041	0.177	0.80	Oxidised fatty acids including prostaglandins
Asparaginyl-Methionine	0.002	0.055	0.65	Peptide
Glycyl-Histidine	0.002	0.055	1.29	Peptide

Glutamylalanine;Hydroxyprolyl-Serine	0.004	0.068	1.34	Peptide
Histidinyl-Valine;Homoanserine	0.009	0.175	1.72	Peptide
Methionyl-Serine	0.015	0.150	0.64	Peptide
Alanyl-Methionine;Cysteinyl-Valine	0.026	0.177	0.27	Peptide
Methionyl-Tyrosine	0.026	0.180	0.79	Peptide
L-gamma-glutamyl-L-isoleucine;L-gamma-glutamyl-L-leucine;Prolyl-Valine	0.041	0.186	1.28	Peptide
Serinyl-Tryptophan	0.041	0.186	1.31	Peptide
Aspartyl-Isoleucine;Aspartyl-Leucine;gamma-glutamyl-L-valine	0.041	0.224	1.33	Peptide
Arginyl-Proline	0.041	0.177	1.43	Peptide
Cysteinyl-Proline;Prolyl-Cysteine	0.041	0.177	1.70	Peptide
Glutaminyl-Glutamine	0.041	0.224	2.91	Peptide
Propane-1,2-diol	0.004	0.102	0.63	Propanoate metabolism
Hypoxanthine	0.002	0.055	0.42	Purine and pyrimidine metabolism
Cytosine	0.002	0.044	0.78	Purine and pyrimidine metabolism
Methylguanosine	0.002	0.044	1.24	Purine and pyrimidine metabolism
5-Aminoimidazole-4-carboxamide	0.002	0.044	1.40	Purine and pyrimidine metabolism
Glycineamideribotide	0.004	0.102	0.59	Purine and pyrimidine metabolism
Dimethylguanosine	0.004	0.068	1.28	Purine and pyrimidine metabolism
Deoxyuridine	0.004	0.072	5.07	Purine and pyrimidine metabolism
N4-Acetylcytidine	0.009	0.107	1.25	Purine and pyrimidine metabolism

Uracil	0.009	0.107	1.27	Purine and pyrimidine metabolism
Orotidine	0.015	0.169	1.29	Purine and pyrimidine metabolism
8-Hydroxyguanosine;Threoneopterin;Neopterin	0.041	0.186	1.40	Purine and pyrimidine metabolism
2-Hydroxy-2-methylbutyric acid;2-hydroxy-3-methyl-butyric acid;2-Hydroxyvaleric acid;3-Hydroxyvaleric acid;4-hydroxy-valeric acid;4-Hydroxyisovaleric acid;5-hydroxy valeric acid	0.002	0.094	1.25	Short chain organic acid
4-Hydroxy-2-oxobutanoic acid;Methylmalonic acid;Succinic acid	0.026	0.180	0.73	Short chain organic acid
2-Methyl-3-ketovaleric acid;3-Methyl-2-oxovaleric acid;Oxohexanoic acid	0.041	0.224	0.39	Short chain organic acid
1alpha,17alpha,21-trihydroxy-20-oxo-22,23,24,25,26,27-hexanorcholecalciferol;"17a,21-Dihydroxy-5b-pregnane-3,11,20-trione";18-Hydroxycorticosterone;"4,5alpha-Dihydrocortisone";Cortisol	0.002	0.084	1.41	Sterol and steroid metabolism
1alpha,21-dihydroxy-20-oxo-22,23,24,25,26,27-hexanorcholecalciferol";11-deoxycortisol;19-Hydroxydeoxycorticoste	0.002	0.094	1.44	Sterol and steroid metabolism
6-dehydrotestosterone;tetramethyl-pentadecahexaenoic acid";Androstenedione;Dehydrotestosterone	0.002	0.084	1.49	Sterol and steroid metabolism
11beta-Hydroxytestosterone;16-Oxoandrostenediol;16a-Hydroxydehydroisoandrosterone;19-Hydroxytestosterone;2beta-Hydroxytestosterone;"3a,16-Dihydroxyandrostenone";"3a,16a-Dihydroxyandrostenone";"3a,16b-Dihydroxyandrostenone";4-Hydroxytestosterone;6beta-Hydroxytestosterone;7a-Hydroxydehydroepiandrosterone;7alpha-Hydroxytestosterone;7b-Hydroxydehydroepiandrosterone	0.002	0.084	1.52	Sterol and steroid metabolism
1alpha,21-dihydroxy-20-oxo-22,23,24,25,26,27-	0.002	0.044	1.65	Sterol and steroid metabolism

hexanorcholecalciferol;11-deoxycortisol;19-Hydroxydeoxycorticosterone;21-Deoxycortisol;"21-Hydroxy-5b-pregnane-3,11,20-trione";7'-Carboxy-alpha-tocotrienol;Corticosterone				
Dehydroepiandrosterone sulfate;Epitestosterone sulfate;Testosterone sulfate	0.002	0.094	1.76	Sterol and steroid metabolism
deoxycorticosterone;Aldosterone;Cortisone	0.002	0.094	1.82	Sterol and steroid metabolism
11b-Hydroxyprogesterone;6-EpHEPE;7'-Carboxy-gamma-tocotrienol;PGA3;PGB3;"PGE3 1,15-lactone";PGJ3;6-dehydrotestosterone;Androstenedione;Dehydrotestosterone	0.002	0.094	2.33	Sterol and steroid metabolism
9'-Carboxy-gamma-tocotrienol	0.004	0.102	2.19	Sterol and steroid metabolism
5a-Dihydrotestosterone sulfate;Androsterone sulfate	0.004	0.072	2.61	Sterol and steroid metabolism
1alpha,24-dihydroxy-22-oxa-20-epicholecalciferol;" 1alpha,24-dihydroxy-22-oxacholecalciferol";" 1alpha,25-dihydroxy-22-oxa-20-epicholecalciferol";" 1alpha,25-dihydroxy-22-oxacholecalciferol";" 1alpha,25-dihydroxy-23-oxacholecalciferol";" 1beta,25-dihydroxy-22-oxacholecalciferol";11'-Carboxy-alpha-chromanol;"1alpha,20,25-trihydroxy-24-norcholecalciferol	0.009	0.139	0.77	Sterol and steroid metabolism
1beta,3alpha,7alpha-trihydroxy-5beta-cholestan-26-oic acid;"3a,7a,12a-Trihydroxy-5b-cholestanoic acid";"3alpha,12alpha,Trihydroxy-5β-cholestan-26-oic acid";"3alpha,7alpha,12alpha-trihydroxy-5alpha-cholestan-26-oic acid";"3alpha,7alpha,12alpha-trihydroxy-5beta-cholestan-26-oic acid";"3alpha,7alpha,12alpha,24-tetrahydroxy-5beta-cholestan-26-al";"3alpha,7alpha,15alpha-Trihydroxy-5beta-cholestan-26-oic acid";"3alpha,7alpha,16alpha-Trihydroxy-5beta-cholestan-26-oic acid";"3alpha,7alpha,24-trihydroxy-5alpha-cholestan-26-oic acid";"3alpha,7beta,12alpha-Trihydroxy-	0.026	0.216	1.28	Sterol and steroid metabolism

5beta-cholestan-26-oic acid";Coprocholic acid;;" 1,25-dihydroxy-2-nor-1,2-secocholecalciferol";" 1alpha,25-dihydroxy-19-norcholecalciferol";"2-Nor-1,3-seco-1alpha,25-dihydroxyvitamin D3				
pregnenolone sulfate	0.026	0.180	1.36	Sterol and steroid metabolism
Isopyridoxal;Pyridoxal	0.002	0.084	1.23	Vitamin B6 metabolism

Supplementary Table 7. Pathway analysis of intracellular metabolome comparing KD siRNA to SCR siRNA (6A) and KD shRNA to SCR shRNA cells (6B). *$p<0.05$ was used as the threshold of significance. Significant metabolites within each pathway are highlighted in bold font.*

Supplementary Table 7A

**Pathways enriched comparing KD siRNA to SCR siRNA cells**	**p value**	**q value**	**Metabolites in pathway (significant metabolites in bold)**
Citrate cycle (TCA cycle)	2.67E-05	0.002	**Succinic acid**; **Oxoglutaric acid**; Enzyme N6-(dihydrolipoyl)lysine; Thiamine pyrophosphate; Enzyme N6-(lipoyl)lysine; 3-carboxy-1-hydroxypropylthiamine diphosphate; Succinyl-CoA; Oxalosuccinic acid; **Isocitric acid**; **Oxalacetic acid**; Acetyl-CoA; **L-Malic acid**; **cis-Aconitic acid**; **Citric acid**; Pyruvic acid; 2-(a-Hydroxyethyl)thiamine diphosphate; **Fumaric acid**; [Dihydrolipoyllysine-residue succinyltransferase] S-succinyldihydrolipoyllysine; S-Acetyldihydrolipoamide-E; Phosphoenolpyruvic acid
Alanine, aspartate and glutamate metabolism	0.0001	0.004	**N-Acetyl-L-aspartic acid**; 2-Oxosuccinamate; **L-Aspartic acid**; L-Asparagine; D-Aspartic acid; Argininosuccinic acid; Adenylsuccinic acid; L-Alanine; Pyruvic acid; Ureidosuccinic acid; Succinic acid semialdehyde; **Oxoglutaric acid**; **L-Glutamine**; **L-Glutamic acid**; Gamma-Aminobutyric acid; Ammonia; 2-Keto-glutaramic acid; (S)-1-Pyrroline-5-carboxylate; **Oxalacetic acid**; **Fumaric acid**; **Succinic acid**; Carbamoylphosphate; Glucosamine 6-phosphate; 5-Phosphoribosylamine

Arginine and proline metabolism	0.0002	0.004	L-Glutamic-gamma-semialdehyde; Pyrroline hydroxycarboxylic acid; **L-Glutamine**; Ammonia; Carbamoylphosphate; Ornithine; **L-Aspartic acid**; Citrulline; Argininosuccinic acid; **L-Arginine**; **L-Glutamic acid**; **N-Acetyl-L-alanine**; N-Acetyl-L-glutamyl 5-phosphate; N-Acetyl-L-glutamate 5-semialdehyde; N-Acetylornithine; **L-Proline**; Peptide; 4-Oxoproline; D-Proline; 1-Pyrroline-2-carboxylic acid; Hydroxyproline; L-4-Hydroxyglutamate semialdehyde; L-erythro-4-Hydroxyglutamate; D-4-Hydroxy-2-oxoglutarate; Nopaline; N-(o)-Hydroxyarginine; Guanidoacetic acid; **Creatine**; N-Carbamoylsarcosine; Phosphocreatine; N-Methylhydantoin; **Creatinine**; **5-Guanidino-2-oxopentanoate**; 4-Guanidinobutanal; 4-Guanidinobutanoic acid; Gamma-Aminobutyric acid; 4-Guanidinobutanamide; Agmatine; N-Carbamoylputrescine; N2-Succinyl-L-arginine; N2-Succinyl-L-ornithine; N2-Succinyl-L-glutamic acid 5-semialdehyde; N-Succinyl-L-glutamate; L-Glutamic acid 5-phosphate; (S)-1-Pyrroline-5-carboxylate; Putrescine; Gamma-glutamyl-L-putrescine; gamma-Glutamyl-gamma-aminobutyraldehyde; 4-(Glutamylamino) butanoate; 4-Aminobutyraldehyde; S-Adenosylmethioninamine; S-Adenosylmethionine; **Spermidine**; **N-Acetylputrescine**; N4-Acetylaminobutanal; 4-Acetamidobutanoic acid; Urea; Urea-1-carboxylate; cis-4-Hydroxy-D-proline; 1-Pyrroline-4-hydroxy-2-carboxylate; **Fumaric acid**; **5-Amino-2-oxopentanoic acid**; 5-Aminopentanoic acid; Pyruvic acid; Glyoxylic acid; N2-(D-1-Carboxyethyl)-L-arginine; **L-Arginine phosphate**; Nitric oxide; Sarcosine; **Spermine**; Carbon dioxide; Homocarnosine; Phosphoguanidinoacetate; 2,5-Dioxopentanoate; Pyrrole-2-carboxylic acid; 2-Oxo-4-hydroxy-5-aminovalerate; Linatine
Pyrimidine metabolism	0.0006	0.012	**Uridine 5'-diphosphate**; Thioredoxin; 3-Oxo-3-ureidopropanoate; Uridine 5'-monophosphate; dCTP; dUMP; **L-Glutamine**; Carbamoylphosphate; 4,5-Dihydroorotic acid; **Orotidylic acid**; RNA; Uridine triphosphate; Cytidine triphosphate; **Uridine**; Dihydrouracil; Ureidopropionic acid; CDP; Cytidine monophosphate; **Cytidine**; **Cytosine**; Uracil; Barbiturate; Thioredoxin disulfide; dCDP; dCMP;

			Deoxycytidine; Deoxyuridine triphosphate; dUDP; Deoxyuridine; Thymidine 5'-triphosphate; dTDP; 5-Thymidylic acid; **Thymidine**; **5-Methylcytosine**; Thymine; **Dihydrothymine**; 5-Methylbarbiturate; Ureidoisobutyric acid; **Pseudouridine**; Uridine diphosphate glucose; 3'-UMP; 2',3'-Cyclic UMP; 3'-CMP; 2',3'-Cyclic CMP; Trimetaphosphate; P1,P4-Bis(5'-uridyl) tetraphosphate; 5-Hydroxymethyldeoxycytidylate; 2'-Deoxy-5-hydroxymethylcytidine-5'-diphosphate; **Malonic acid**; Urea; Ureidosuccinic acid; Orotic acid; Phosphoribosyl pyrophosphate; Beta-Alanine; DNA; Deoxyribose 1-phosphate; **Methylmalonic acid**; 3-Aminoisobutanoic acid; Pseudouridine 5'-phosphate; 2'-Deoxy-5-hydroxymethylcytidine-5'-triphosphate
Glyoxylate and dicarboxylate metabolism	0.0017	0.027	**Hydroxypyruvic acid**; **cis-Aconitic acid**; Glyoxylic acid; **Oxoglutaric acid**; Pentanoyl-CoA; Butanoyl-CoA; 3-Oxalomalate; 4-Hydroxy-2-oxoglutaric acid; **Isocitric acid**; **N-Formyl-L-glutamic acid**; N,N-Dimethylformamide; Formamide; N-Formyl-L-methionine; N-Formyl-L-methionylaminoacyl-tRNA; N10-Formyl-THF; L-Formylkynurenine; N-Formyl-L-aspartate; 5,10-Methenyltetrahydrofolic acid; Formic acid; Oxalyl-CoA; Glycolic acid; Oxalic acid; Formyl-CoA; H+; Hydrogen; **Citric acid**; Glycolaldehyde; Ethylene glycol; **Glyceric acid**; **L-Malic acid**; Malyl-CoA; Dihydroxyfumaric acid; trans-2,3-Epoxysuccinate; meso-Tartaric acid; Tartaric acid; D-Ribulose 1,5-bisphosphate; Phosphoglycolic acid; 2-Hydroxy-3-oxosuccinate; Tartronate semialdehyde; 2-Hydroxy-3-oxoadipate; 3-Propylmalate; 3-Ethylmalate; **Oxalacetic acid**; Pyruvic acid; **Succinic acid**; 5,10-Methylene-THF; Formyl phosphate; Carbon dioxide; Acetyl-CoA; 3-Phospho-D-glycerate
Butanoate metabolism	0.005	0.066	3-Butyn-1-ol; 3-Butyn-1-al; 3-Butynoate; **(R)-3-Hydroxybutyric acid**; (R)-3-((R)-3-Hydroxybutanoyloxy)butanoate; Acetoacetic acid; 3-Hydroxy-3-methylglutaryl-CoA; Acetyl-CoA; Acetoacetyl-CoA; (S)-3-Hydroxybutanoyl-CoA; 3-Hydroxybutyryl-CoA; Poly-beta-hydroxybutyrate; Crotonoyl-CoA; Vinylacetyl-CoA; **4-Hydroxybutyric acid**; Gamma-Aminobutyric acid; **L-Glutamic acid**; Pyruvic acid; Butanoyl-CoA; Butanal; Succinic acid

			semialdehyde; **Butyric acid**; (R)-Malate; **Maleic acid**; **Succinic acid**; Thiamine pyrophosphate; 2-(a-Hydroxyethyl)thiamine diphosphate; 2-Acetolactate ; (S)-Acetoin; (R)-Acetoin; 2-Hydroxyglutaryl-CoA; 2-Hydroxyglutarate; Glutaconyl-1-CoA; **Oxoglutaric acid**; Butanoylphosphate; 1-Butanol; **Fumaric acid**; (R,R)-Butane-2,3-diol; (S,S)-Butane-2,3-diol; Diacetyl
Histidine metabolism	0.009	0.103	4-Imidazolone-5-propionic acid; Formiminoglutamic acid; **N-Formyl-L-glutamic acid**; **L-Glutamic acid**; Urocanic acid; L-Histidine; Anserine; Carnosine; N-Formimino-L-aspartate; Imidazoleacetic acid; Imidazole-4-acetaldehyde; 1-Methylhistamine; Methylimidazole acetaldehyde; Histamine; Phosphoribosyl-ATP; L-Histidinal; **L-Histidinol**; L-Histidinol phosphate; D-Erythro-imidazole-glycerol-phosphate; PhosphoribosylformiminoAICAR-phosphate; Phosphoribosyl-AMP; Phosphoribulosylformimino-AICAR-P; 1-Methylhistidine; N-Formyl-L-aspartate; 4-Imidazolone-5-acetate; Hercynine; Ergothioneine; **Hydantoin-5-propionic acid**; Formylisoglutamine; 4-Oxoglutaramate; Imidazol-5-yl-pyruvate; Imidazoleacetic acid ribotide; N-Carbamyl-L-glutamate; Methylimidazoleacetic acid; Phosphoribosyl pyrophosphate; Imidazole acetol-phosphate; **AICAR**; **L-Aspartic acid**; 4-(beta-Acetylaminoethyl)imidazole; Thiourocanic acid; Isoglutamine; **Oxoglutaric acid**; Imidazole lactate; **Imidazoleacetic acid riboside**
D-Arginine and D-ornithine metabolism	0.014	0.134	2R,4S)-2,4-Diaminopentanoate; D-Ornithine; D-Arginine; **L-Arginine**; 2-Amino-4-oxopentanoic acid; **5-Amino-2-oxopentanoic acid**; Ornithine; **5-Guanidino-2-oxopentanoate**
Glycine, serine and threonine metabolism	0.015	0.134	Betaine aldehyde; L-Serine; Ectoine; Choline; N-gamma-Acetyldiaminobutyrate; L-2,4-Diaminobutanoate; L-Aspartate-semialdehyde; 3-Phospho-D-glycerate; **Glyceric acid**; Betaine; Guanidoacetic acid; Dimethylglycine; L-Cystathionine; Glycine; **L-Aspartic acid**; **Phosphoserine**; Sarcosine; 5,10-Methylene-THF; L-Threonine; O-Phosphohomoserine; L-Aspartyl-4-phosphate; **L-Homoserine**; Lipoylprotein; D-Serine; Aminoacetone; Pyruvaldehyde;

			Tetrahydrofolic acid; S-Aminomethyldihydrolipoylprotein; D-Lombricine; Dihydrolipoylprotein; **Creatine**; 5-Hydroxyectoine; **Hydroxypyruvic acid**; Phosphohydroxypyruvic acid; L-Cysteine; L-Allothreonine; 2-Ketobutyric acid; Glyoxylic acid; L-2-Amino-3-oxobutanoic acid; Pyruvic acid; Carbon dioxide; **5-Aminolevulinic acid**; Hydroxyacetone; (R)-1-Aminopropan-2-ol; Ammonia; N-Phospho-D-lombricine; PS(16:0/16:0); **L-Tryptophan**
Nicotinate and nicotinamide metabolism	0.029	0.213	(R,S)-Nicotine; (2R,3S)-2,3-Dimethylmalate; 2,3-Dimethylmaleate; Methylitaconate; 2-Methyleneglutarate; 2-Formylglutarate; 6-Oxo-1,4,5,6-tetrahydronicotinate; **6-Hydroxynicotinic acid**; 2,6-Dihydroxypyridine; 2,6-Dihydroxypseudooxynicotine; 6-Hydroxypseudooxynicotine; (S)-6-Hydroxynicotine; (R)-6-Hydroxynicotine; **L-Aspartic acid**; NADP; Nicotinic acid adenine dinucleotide; Iminoaspartic acid; **Dihydroxyacetone phosphate**; Quinolinic acid; **Maleic acid**; Maleamate; 2,5-Dihydroxypyridine; Nicotinic acid; Nicotinic acid mononucleotide; Nicotinate D-ribonucleoside; Nicotinamide ribotide; **Niacinamide**; NAD; **Nicotinamide riboside**; 1-Methylnicotinamide; 1-Methylpyrrolinium; (S)-2-(Hydroxymethyl)glutarate; 2,3,6-Trihydroxypyridine; Pyruvic acid; Propionic acid; 2,6-Dihydroxynicotinate; 4-Methylaminobutyrate; **Fumaric acid**; Trigonelline; N1-Methyl-4-pyridone-3-carboxamide; N1-Methyl-2-pyridone-5-carboxamide; Nicotine imine; Blue pigment; 2,6-Dihydroxy-N-methylmyosmine
Aminoacyl-tRNA biosynthesis	0.031	0.213	tRNA(Asn); L-Asparagine; tRNA(His); L-Histidine; tRNA(Phe); L-Phenylalanine; **L-Arginine**; tRNA(Arg); tRNA(Gln); **L-Glutamine**; tRNA(Cys); L-Cysteine; tRNA(Gly); Glycine; tRNA(Asp); **L-Aspartic acid**; tRNA(Ser); L-Serine; L-Methionine; tRNA(Met); L-Valine; tRNA(Val); tRNA(Ala); L-Alanine; tRNA(Lys); L-Lysine; tRNA(Ile); **L-Isoleucine**; tRNA(Leu); **L-Leucine**; L-Threonine; tRNA(Thr); tRNA(Trp); **L-Tryptophan**; L-Methionyl-tRNA; N10-Formyl-THF; **L-Tyrosine**; tRNA(Tyr); **L-Proline**; tRNA(Pro); tRNA(Glu); **L-Glutamic acid**;

			Glutaminyl-tRNA; L-Asparaginyl-tRNA(Asn); O-Phosphoseryl-tRNA(Cys); **Phosphoserine**; tRNA(Sec); L-Seryl-tRNA(Sec); O-Phosphoseryl-tRNA(Sec); L-Pyrrolysine; tRNA(Pyl); L-Histidyl-tRNA(His); L-Phenylalanyl-tRNA(Phe); L-Arginyl-tRNA(Arg); L-Cysteinyl-tRNA(Cys); Glycyl-tRNA(Gly); L-Aspartyl-tRNA(Asp); L-Seryl-tRNA(Ser); L-Valyl-tRNA(Val); L-Alanyl-tRNA; L-Lysyl-tRNA; L-Isoleucyl-tRNA(Ile); L-Leucyl-tRNA; L-Threonyl-tRNA(Thr); L-Tryptophanyl-tRNA(Trp); Tetrahydrofolic acid; N-Formylmethionyl-tRNA; L-Tyrosyl-tRNA(Tyr); L-Prolyl-tRNA(Pro); L-Glutamyl-tRNA(Glu); L-Glutamyl-tRNA(Gln); L-Aspartyl-tRNA(Asn); L-Selenocysteinyl-tRNA(Sec); L-Pyrrolysyl-tRNA(Pyl); L-Lysyl-tRNA(Pyl)
Phenylalanine metabolism	0.032	0.213	4-Hydroxy-2-oxopentanoate; L-Phenylalanine; Phenylacetaldehyde; Phenylacetic acid; Phenylacetyl-CoA; 2-Hydroxy-2,4-pentadienoate; 2-Hydroxy-6-oxonona-2,4-diene-1,9-dioate; 2-Hydroxy-6-ketononatrienedioate; 3-(2,3-Dihydroxyphenyl)propanoate; trans-2,3-Dihydroxycinnamate; m-Coumaric acid; **Phenylethylamine**; **Phenylpyruvic acid**; Phenyllactate; D-Phenylalanine; Phenylethyl alcohol; 2-Phenylacetamide; trans-Cinnamic acid; cis-3-(3-Carboxyethenyl)-3,5-cyclohexadiene-1,2-diol; 3-(2-Hydroxyphenyl)propanoate; 3-(3-Hydroxyphenyl)propanoic acid; cis-3-(Carboxy-ethyl)-3,5-cyclo-hexadiene-1,2-diol; Hydrocinnamic acid; Benzoic acid; Hippuric acid; trans-2-Hydroxycinnamate; **4-Hydroxycinnamic acid**; Phenylglyoxylic acid; Phenylglyoxylyl-CoA; Pyruvic acid; Acetaldehyde; Alpha-N-Phenylacetyl-L-glutamine; Phenylacetylglycine; **Succinic acid**; **Fumaric acid**; Ortho-Hydroxyphenylacetic acid; **Enol-phenylpyruvate**; N-Acetyl-D-phenylalanine; N-Acetyl-L-phenylalanine; Benzoyl-CoA; 4-Hydroxybenzoic acid; p-Hydroxyphenylacetic acid; Salicylic acid; **L-Tyrosine**; 3-Hydroxyphenylacetic acid
D-Glutamine and D-glutamate metabolism	0.035	0.217	D-Glutamyl-peptide; D-Glutamine; UDP-N-acetylmuraminate; D-Glutamic acid; UDP-N-acetylmuramoyl-L-alanine; **L-Glutamic acid**; **L-Glutamine**; 5-D-Glutamyl-D-glutamyl-peptide; UDP-N-acetylmuramoyl-L-alanyl-D-glutamate; Pyrrolidonecarboxylic acid; **Oxoglutaric acid**

Glutathione metabolism	0.043	0.245	Gamma-Glutamylcysteine; R-S-Cysteinylglycine; R-S-Glutathione; **Glutathione**; **Oxidized glutathione**; NADP; NADPH; Glycine; L-Cysteine; **L-Glutamic acid**; Cysteinylglycine; **Pyroglutamic acid**; L-Amino acid; 5-L-Glutamyl-L-alanine; S-Substituted L-cysteine; Acetyl-CoA; RX; **Spermidine**; Glutathionylspermidine; Trypanothione; Dehydroascorbate; Tryparedoxin disulfide; Ornithine; Putrescine; **Spermine**; Glutathionylspermine; Cadaverine; Aminopropylcadaverine; Glutathionylaminopropylcadaverine; Homotrypanothione; Ascorbic acid; Trypanothione disulfide; Tryparedoxin; Bis(glutathionyl)spermine disulfide; Homotrypanothione disulfide; Bis-gamma-glutamylcystine; S-Substituted N-acetyl-L-cysteine; Bis(glutathionyl)spermine
Nitrogen metabolism	0.048	0.256	L-Phenylalanine; **L-Tyrosine**; Nitrite; Formamide; Ammonia; Carbamic acid; Cyanate; Carbon dioxide; Hydroxylamine; **L-Tryptophan**; L-threo-3-Methylaspartate; alpha-Amino acid; **Taurine**; Nitrate; Ethylnitronate; Nitroethane; Nitrogen; Nitric oxide; Nitrous oxide; **L-Aspartic acid**; L-Asparagine; **L-Glutamic acid**; **L-Glutamine**; 2-Aminobenzoic acid; L-Cystathionine; L-Homocysteine; Allocystathionine; Amine; Amide; Cyclic amidines; Amidines; Nitrile; L-Histidine; Carbamoylphosphate; Glycine; Formic acid; Carbonic acid; Adenosine monophosphate; NH4OH

Supplementary Table 7B

Pathway	p value	q value	Metabolites in pathway (significant metabolites in bold)
Purine metabolism	0.0006	0.049	**Guanosine diphosphate**; Xanthine; Ureidoglycine; Allantoic acid; (S)-Ureidoglycolic acid; Carbamoylphosphate; D-Ribulose 5-phosphate; Phosphoribosyl pyrophosphate; **L-Glutamine**; 5-Phosphoribosylamine; Glycineamideribotide; Phosphoribosylformylglycineamidine; AICAR; SAICAR; 5-amino-1-(5-phospho-D-ribosyl)imidazole-4-carboxylate; Phosphoribosyl formamidocarboxamide; RNA; Cyclic AMP; Adenosine triphosphate; dATP; **ADP**; dADP; Adenosine monophosphate; Adenylsuccinic acid; Inosinic acid; **Adenosine**; Deoxyadenosine monophosphate; Deoxyadenosine; Deoxyinosine; Xanthosine; Inosine; Adenine; IDP; Guanosine monophosphate; Xanthylic acid; Hypoxanthine; Guanine; **Deoxyguanosine**; (S)(+)-Allantoin; Uric acid; Urate-3-ribonucleoside; Urea; Adenosine phosphosulfate; 5-Hydroxyisourate; Guanosine 3',5'-bis(diphosphate); Guanosine 3'-diphosphate 5'-triphosphate; Guanosine triphosphate; 2'-Deoxyguanosine 5'-monophosphate; **dGDP**; Guanosine; dGTP; Cyclic GMP; 5-Aminoimidazole; N-Formiminoglycine; Diadenosine tetraphosphate; Sulfate; Phosphoadenosine phosphosulfate; 5'-Phosphoribosyl-N-formylglycinamide; Inosine triphosphate; Inosine 5'-tetraphosphate; Xanthosine 5-triphosphate; 3'-AMP; Guanosine 3'-phosphate; Guanosine 2',3'-cyclic phosphate ; P1,P4-Bis(5'-xanthosyl) tetraphosphate; Adenosine 2',3'-cyclic phosphate; Adenosine diphosphate ribose; Adenosine tetraphosphate; 5-Ureido-4-imidazole carboxylate; 5-Amino-4-imidazole carboxylate; Imidazolone; dIDP; 2'-Deoxyinosine triphosphate; Diadenosine triphosphate; 5'-Butyrylphosphoinosine; Acetyl adenylate; 5'-Benzoylphosphoadenosine; 5-Hydroxy-2-oxo-4-ureido-2,5-dihydro-1H-imidazole-5-carboxylate; 5-Aminoimidazole ribonucleotide; 5-Carboxyamino-1-(5-phospho-D-ribosyl)imidazole; Oxalureate; DNA; Ammonia; Glyoxylic acid; Carbon dioxide; (R)(-)-Allantoin; Glycine; Adenosine 3',5'-diphosphate; Diguanosine

			tetraphosphate; alpha-D-Ribose 1-phosphate; dIMP; 5-Amino-4-imidazolecarboxyamide
Pyrimidine metabolism	0.005	0.208	**Uridine 5'-diphosphate**; Thioredoxin; 3-Oxo-3-ureidopropanoate; Uridine 5'-monophosphate; dCTP; dUMP; **L-Glutamine**; Carbamoylphosphate; 4,5-Dihydroorotic acid; **Orotidylic acid**; RNA; Uridine triphosphate; Cytidine triphosphate; Uridine; Dihydrouracil; Ureidopropionic acid; CDP; Cytidine monophosphate; Cytidine; Cytosine; Uracil; Barbiturate; Thioredoxin disulfide; dCDP; dCMP; Deoxycytidine; Deoxyuridine triphosphate; dUDP; Deoxyuridine; Thymidine 5'-triphosphate; dTDP; 5-Thymidylic acid; **Thymidine**; 5-Methylcytosine; Thymine; Dihydrothymine; 5-Methylbarbiturate; Ureidoisobutyric acid; Pseudouridine; Uridine diphosphate glucose; 3'-UMP; 2',3'-Cyclic UMP; 3'-CMP; 2',3'-Cyclic CMP; Trimetaphosphate; P1,P4-Bis(5'-uridyl) tetraphosphate; 5-Hydroxymethyldeoxycytidylate; 2'-Deoxy-5-hydroxymethylcytidine-5'-diphosphate; Malonic acid; Urea; Ureidosuccinic acid; Orotic acid; Phosphoribosyl pyrophosphate; Beta-Alanine; DNA; Deoxyribose 1-phosphate; Methylmalonic acid; 3-Aminoisobutanoic acid; Pseudouridine 5'-phosphate; 2'-Deoxy-5-hydroxymethylcytidine-5'-triphosphate

Arginine and proline metabolism	0.012	0.334	L-Glutamic-gamma-semialdehyde; Pyrroline hydroxycarboxylic acid; **L-Glutamine**; Ammonia; Carbamoylphosphate; Ornithine; L-Aspartic acid; Citrulline; Argininosuccinic acid; L-Arginine; L-Glutamic acid; N-Acetyl-L-alanine; N-Acetyl-L-glutamyl 5-phosphate; N-Acetyl-L-glutamate 5-semialdehyde; N-Acetylornithine; **L-Proline**; Peptide; 4-Oxoproline; D-Proline; 1-Pyrroline-2-carboxylic acid; Hydroxyproline; L-4-Hydroxyglutamate semialdehyde; L-erythro-4-Hydroxyglutamate; D-4-Hydroxy-2-oxoglutarate; Nopaline; N-(o)-Hydroxyarginine; Guanidoacetic acid; **Creatine**; N-Carbamoylsarcosine; Phosphocreatine; N-Methylhydantoin; Creatinine; 5-Guanidino-2-oxopentanoate; 4-Guanidinobutanal; 4-Guanidinobutanoic acid; Gamma-Aminobutyric acid; 4-Guanidinobutanamide; Agmatine; N-Carbamoylputrescine; N2-Succinyl-L-arginine; N2-Succinyl-L-ornithine; N2-Succinyl-L-glutamic acid 5-semialdehyde; N-Succinyl-L-glutamate; L-Glutamic acid 5-phosphate; (S)-1-Pyrroline-5-carboxylate; Putrescine; Gamma-glutamyl-L-putrescine; gamma-Glutamyl-gamma-aminobutyraldehyde; 4-(Glutamylamino) butanoate; 4-Aminobutyraldehyde; S-Adenosylmethioninamine; S-Adenosylmethionine; Spermidine; N-Acetylputrescine; N4-Acetylaminobutanal; 4-Acetamidobutanoic acid; Urea; Urea-1-carboxylate; cis-4-Hydroxy-D-proline; 1-Pyrroline-4-hydroxy-2-carboxylate; Fumaric acid; 5-Amino-2-oxopentanoic acid; 5-Aminopentanoic acid; Pyruvic acid; Glyoxylic acid; N2-(D-1-Carboxyethyl)-L-arginine; L-Arginine phosphate; Nitric oxide; Sarcosine; **Spermine**; Carbon dioxide; Homocarnosine; Phosphoguanidinoacetate; 2,5-Dioxopentanoate; Pyrrole-2-carboxylic acid; 2-Oxo-4-hydroxy-5-aminovalerate; Linatine

References

1. Juhlen R, Idkowiak J, Taylor AE, Kind B, Arlt W, Huebner A, et al. Role of ALADIN in human adrenocortical cells for oxidative stress response and steroidogenesis. PLoS One. 2015;10(4):e0124582.

2. Lebbe M, Taylor AE, Visser JA, Kirkman-Brown J, Woodruff TK, Arlt W. The steroid metabolome in the isolated ovarian follicle and its response to androgen exposure and antagonism. Endocrinology. 2017.

3. Dobin A, Davis CA, Schlesinger F, Drenkow J, Zaleski C, Jha S, et al. STAR: ultrafast universal RNA-seq aligner. Bioinformatics. 2013;29(1):15-21.

4. Anders S, Pyl PT, Huber W. HTSeq--a Python framework to work with high-throughput sequencing data. Bioinformatics. 2015;31(2):166-9.

5. Love MI, Huber W, Anders S. Moderated estimation of fold change and dispersion for RNA-seq data with DESeq2. Genome Biol. 2014;15(12):550.

6. Strimmer K. fdrtool: a versatile R package for estimating local and tail area-based false discovery rates. Bioinformatics. 2008;24(12):1461-2.

7. Luo W, Friedman MS, Shedden K, Hankenson KD, Woolf PJ. GAGE: generally applicable gene set enrichment for pathway analysis. BMC Bioinformatics. 2009;10:161.

8. Meimaridou E, Goldsworthy M, Chortis V, Fragouli E, Foster PA, Arlt W, et al. NNT is a key regulator of adrenal redox homeostasis and steroidogenesis in male mice. J Endocrinol. 2017.

9. Smith CA, Want EJ, O'Maille G, Abagyan R, Siuzdak G. XCMS: processing mass spectrometry data for metabolite profiling using nonlinear peak alignment, matching, and identification. Anal Chem. 2006;78(3):779-87.

10. Dunn WB, Broadhurst D, Brown M, Baker PN, Redman CW, Kenny LC, et al. Metabolic profiling of serum using Ultra Performance Liquid Chromatography and the LTQ-Orbitrap mass spectrometry system. J Chromatogr B Analyt Technol Biomed Life Sci. 2008;871(2):288-98.

11. Brown M, Wedge DC, Goodacre R, Kell DB, Baker PN, Kenny LC, et al. Automated workflows for accurate mass-based putative metabolite identification in LC/MS-derived metabolomic datasets. Bioinformatics. 2011;27(8):1108-12.

12. Dunn WB, Broadhurst D, Begley P, Zelena E, Francis-McIntyre S, Anderson N, et al. Procedures for large-scale metabolic profiling of serum and plasma using gas chromatography and liquid chromatography coupled to mass spectrometry. Nat Protoc. 2011;6(7):1060-83.

13. Xia J, Sinelnikov IV, Han B, Wishart DS. MetaboAnalyst 3.0--making metabolomics more meaningful. Nucleic Acids Res. 2015;43(W1):W251-7.
